# Supplementary material for: hiPSC-derived bone marrow milieu identifies a clinically actionable driver of niche-mediated treatment resistance in leukemia
Source: Cell Rep Med. 2022 Aug 16;3(8):100717. doi: 10.1016/j.xcrm.2022.100717 (PMC9418860; doi:10.1016/j.xcrm.2022.100717)
Supplement: Document S1. Figures S1–S7 and Tables S1 and S2 [file mmc1.pdf]

Cell Reports Medicine, Volume *3*

Supplemental information

hiPSC-derived bone marrow milieu identifies a clinically actionable driver of niche-mediated treatment resistance in leukemia

Deepali Pal, Helen Blair, Jessica Parker, Sean Hockney, Melanie Beckett, Mankaran Singh, Ricky Tirtakusuma, Ryan Nelson, Hesta McNeill, Sharon H. Angel, Aaron Wilson, Salem Nizami, Sirintra Nakjang, Peixun Zhou, Claire Schwab, Paul Sinclair, Lisa J. Russell, Jonathan Coxhead, Christina Halsey, James M. Allan, Christine J. Harrison, Anthony V. Moorman, Olaf Heidenreich, and Josef Vormoor

Supplemental information:

hiPSC-derived bone marrow milieu identifies a clinically actionable driver of niche-mediated treatment resistance in leukemia

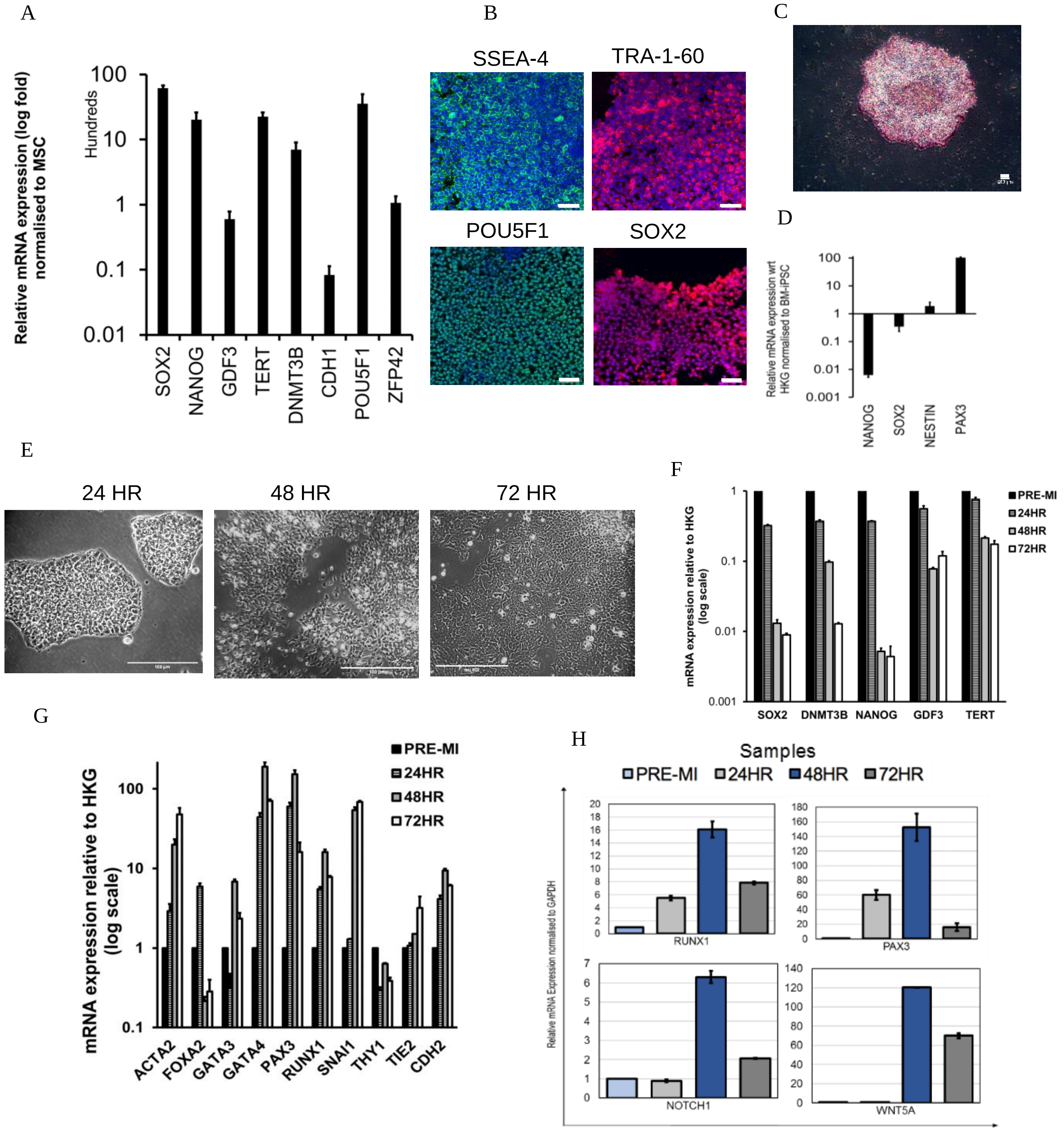

S1. Related to Fig.1. Development and characterization of MYC and virus free BM-iPSC. A. Relative mRNA expression of pluripotent transcripts in BM-iPSC wrt HKG (GAPDH) B. Cell surface marker SSEA4, TRA-1-60 staining and nuclear POU5F1 and SOX2, in feeder-free and xeno-free cultures of BM-iPSC. Scale bar = 100µM C. Image showing BM-iPSC stained for Alkaline phosphatase. Scale bar = 100µM D. mRNA expression wrt HKG (GAPDH) of in vitro differentiation of BM-iPSC derived embryoid bodies E. Photomicrograph of BM-iPSC differentiation into early mesoderm cells over 72 hours. Scale bar = 100µM F. mRNA expression of pluripotent transcripts (HKG = GAPDH) during mesodermal differentiation of BM-iPSC: pre mesoderm induction and at 24, 48 and 72 hours after mesoderm induction. G. mRNA expression (HKG = GAPDH) of mesodermal genes during mesodermal differentiation H. Relative mRNA expression of mesoderm genes RUNX1, PAX3 and NOTCH1 and WNT5A during mesodermal differentiation of BM-iPSC

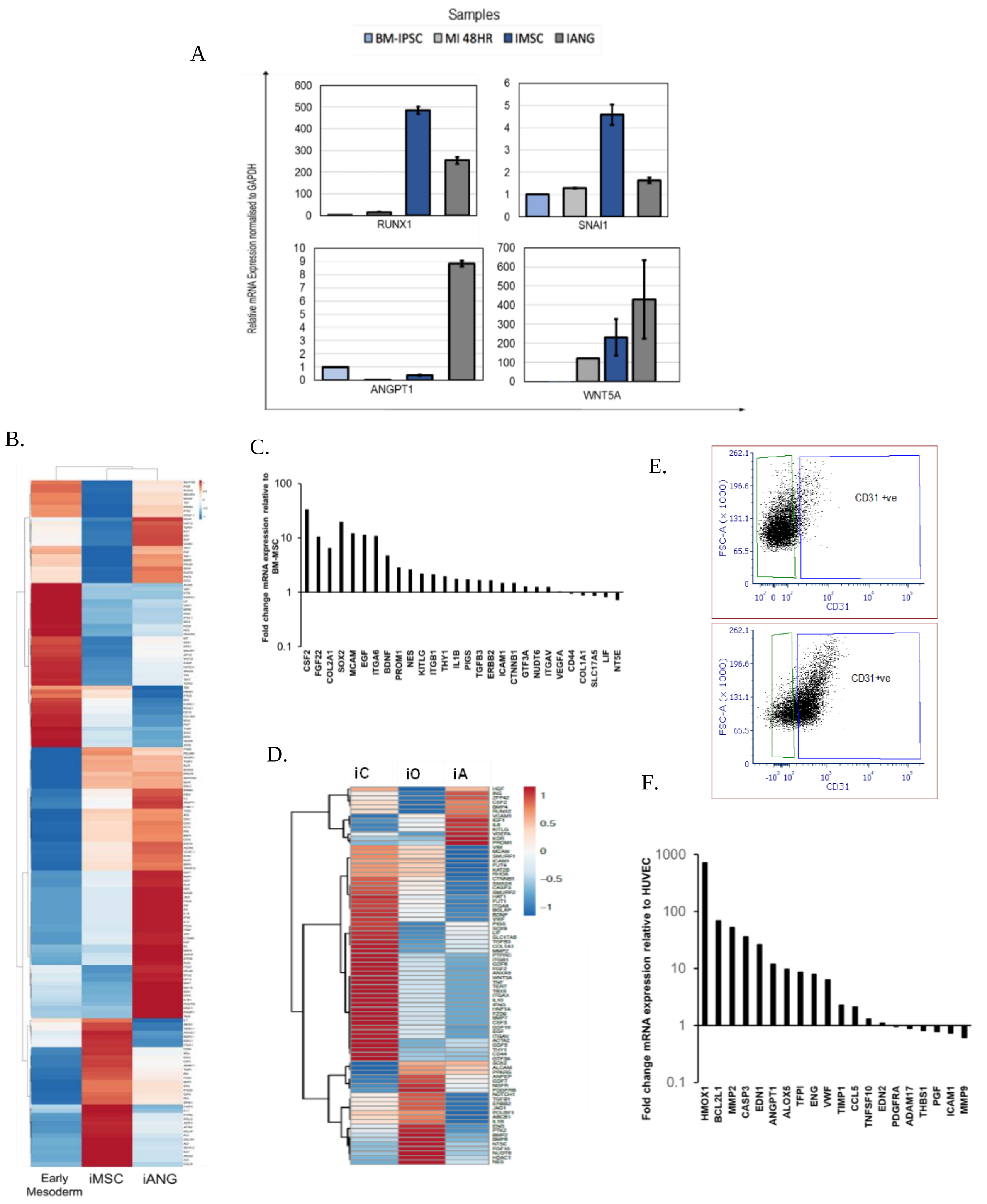

S2. Related to Fig.1. BM-iPSC generate mesenchymal (iMSC) and angiogenic (iANG) niche cells. A. Relative mRNA expression of messenchymal genes RUNX1, SNAI1 and ANGPT1 and WNT5A during in early mesoderm (mesoderm induction, MI at 48 hrs), iMSC and iANG B. BM-iPSC derived early mesoderm, mesenchymal [iMSC] and vascular [iANG] cells demonstrate distinct transcriptomic profiles as evaluated by high throughput qPCR gene expression arrays. C. mRNA expression in iMSC normalised to BM-MSC. D. iMSC differentiate into chondrocytes [iC], osteocytes [iO] and adipocytes [iA] with distinct gene expression profiles E. CD31 expression in iANG cells stained with CD31 antibody (bottom dot plot) versus when stained with isotype control (top dot plot). 54% cells are CD31+ve and 46% are CD31-ve in the stained sample versus 1% within the CD31+ve gate for the relevant isotype control. F. mRNA expression in iPSC-derived endothelia normalised to HUVEC cells.

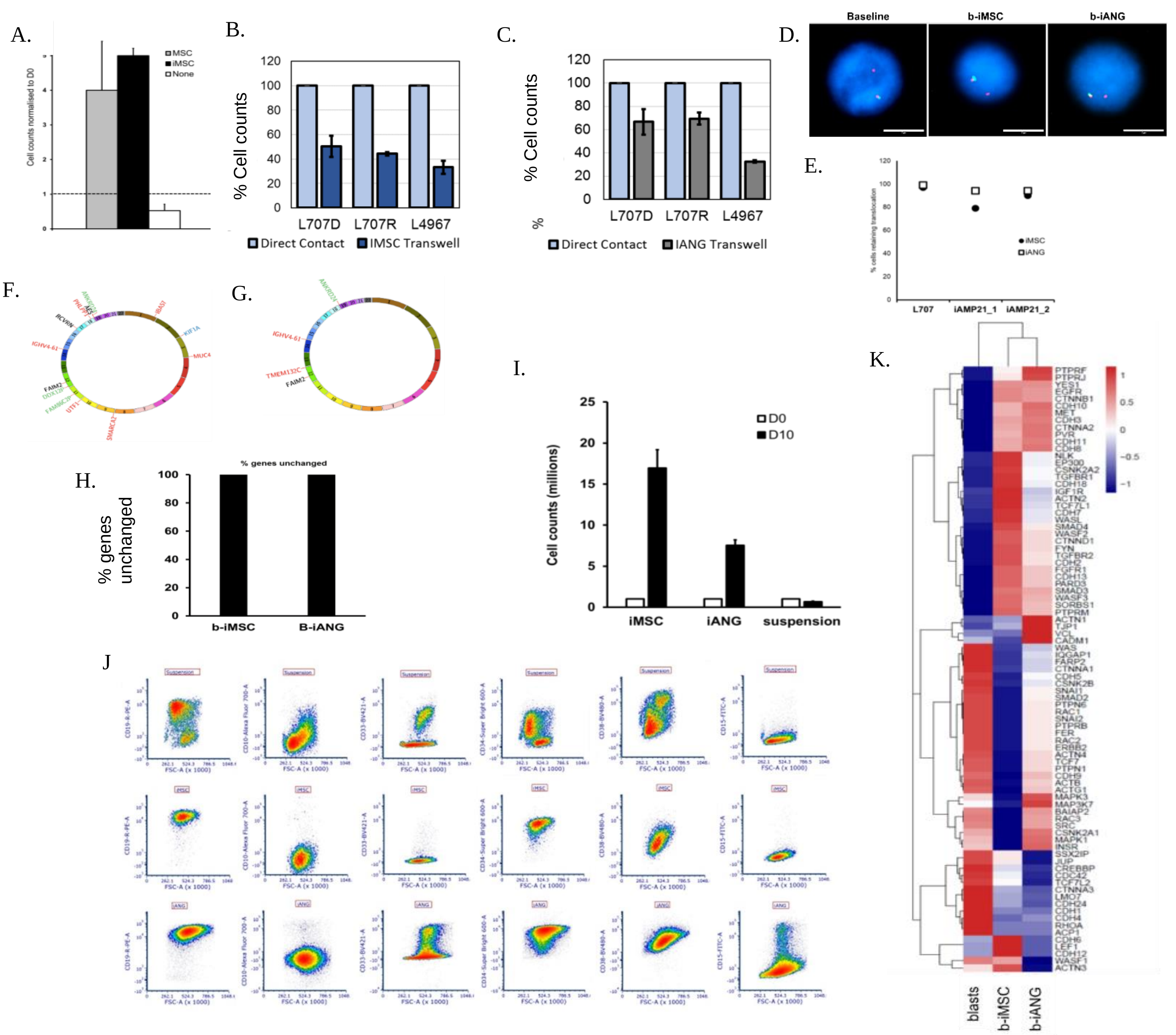

S3. Related to Fig.2. Characterisation of niche primed leukemia cells. A. Counts of patient ALL cells on primary MSC, iMSC and in feeder-free suspension(None) over 7 days. Dotted line = starting cell count. B-C. Cell counts of ALL cells on direct contact and transwell co-cultures with B. iMSC and C. iANG over 7 days with cell counts in direct contact cultures standardized to 100%. D. FISH images of E2A-HLF break-apart probe showing 1 breakpoint and 1 intact loci in baseline and retention of this staining in b-iMSC, b-iANG scale bar = 10 µm. E. Scoring data confirms retention of initial cytogenetic translocation in niche primed ALL. L707 = E2A breakpoint; iAMP21 = additional copies of RUNX1 which is a feature of iAMP21 (abnormal amplification of chromosome 21) samples. F-G. Circos plots showing whole exome changes in patient blasts following co-culture on F. iMSC and G. iANG over 4 weeks. Green=silent:synonymous_variant/non_coding_transcript_exon_variant, black= UTR_variant, red = missense variant, blue = inframe deletion H. % exomes that are unchanged in patient blasts following i-niche co-culture. I. Cell counts from a patient with biphenotypic MLLre leukaemia [MS40] on iMSC, iANG and in niche-free suspension cultures over 7 days. J. Immunophenotyping of MS40 blasts in niche-free suspension culture [top panel] and MS40 blasts primed by iMSC and iANG [middle, bottom panel] after 7 days.K. RNA Sequencing data showing adhesion molecules expression in patient ALL sample L707D blasts before co-culture are compared to blasts following a 7-day co-culture on iMSC (b-iMSC) and iANG (b-iANG).

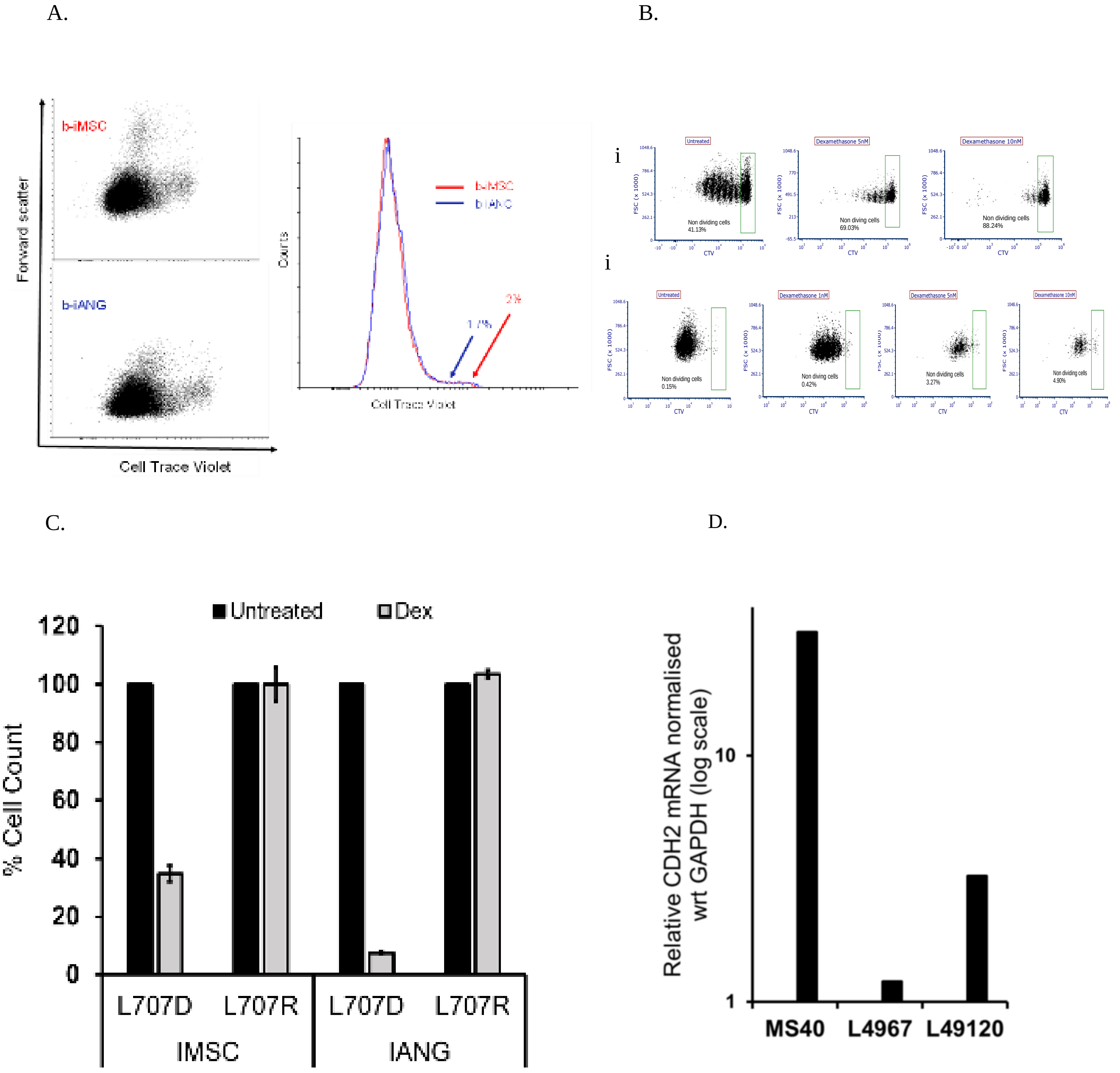

S4. Related to Fig.3. Under Dexamethasone treatment pressure CDH2 is upregulated by iMSC-primed cycling cells. A. Fast and slow dividing niche primed blasts from relapse sample (L707R) on iMSC [red] and iANG [blue] at Day 7. CTV = Cell trace violet dye B. Cell generational tracing dot plots showing slow cycling leukaemia cells when co-cultured on i. iANG and ii. iMSC without and with dexamethasone treatment over a seven day period. C. Cell counts following 10nM dexamethasone treatment on patient leukaemia cells at diagnosis [L707D] and relapse[L707R]. Counts taken at day 7. D. CDH2 mRNA levels in fast cycling iMSC-primed patient leukaemia cells (samples MS40, L4967, L49120) standardised against slow-cycling cells under dexamethasone pressure.

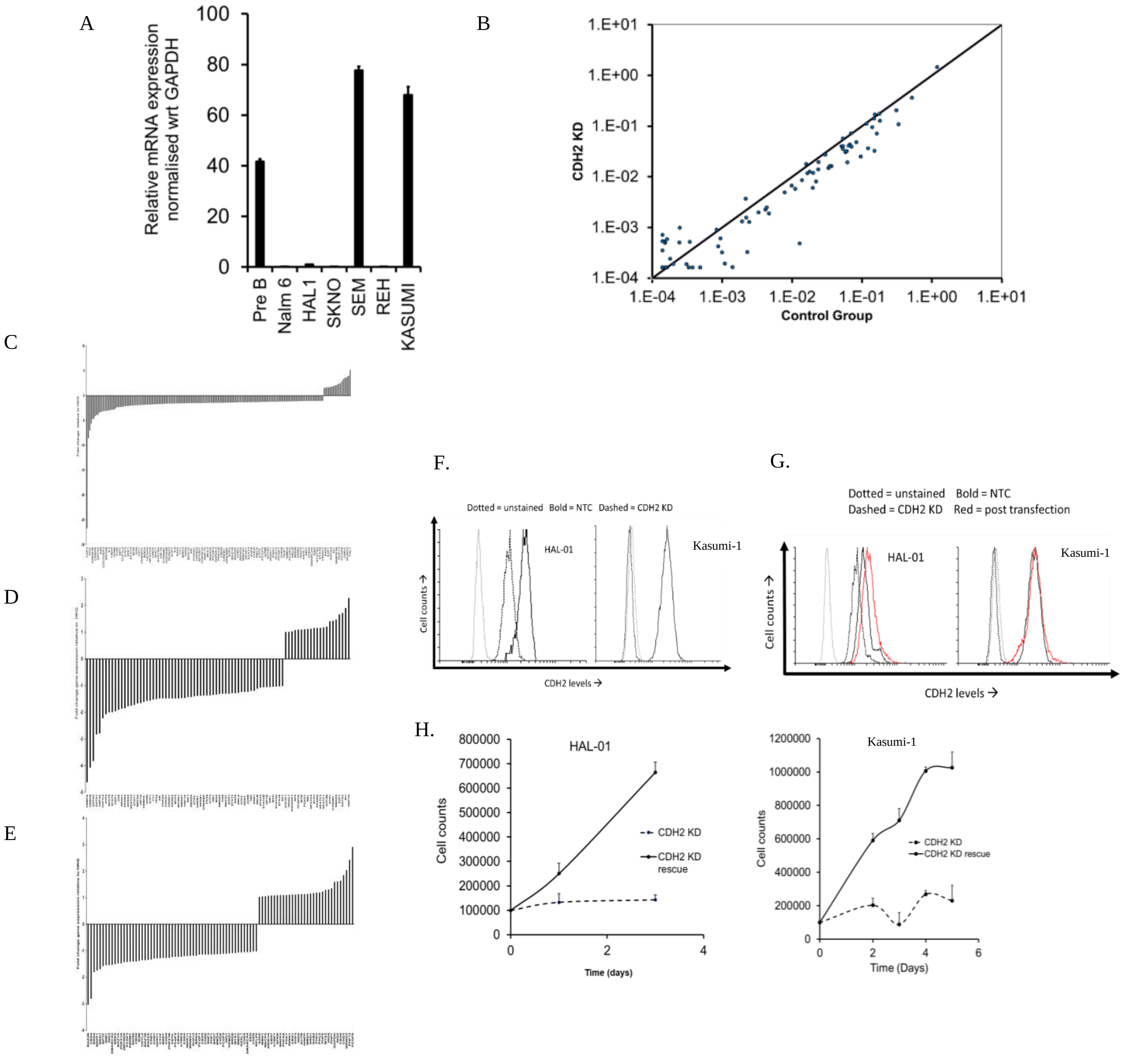

S5. Related to Fig.4. CDH2 drives leukemia proliferation and reduces sensitivity against Dexamethasone. A. CDH2 mRNA levels in cell lines B. Scatter plot showing gene expression in control and CDH2 knockdown in SEM leukaemia cells. C-E. Fold change in qPCR gene expression following CDH2 shRNA knockdown in leukaemic cell line SEM. Genes profiled include C. those that play a role in human leukaemogenesis. D. Transcription factor and E. Chromatin remodelling factors F. Flow cytometry data showing CDH2 expression in ALL (HAL-01) and AML (Kasumi-1) cells following CDH2 knockdown experiments. Bold line represents non targeting control, NTC and dashed line represents CDH2 knockdown cells. G. Rescue of CDH2 protein expression in knockdown cells following transfection with an exogenous optimised CDH2 sequence H. Cell counts in CDH2 shRNA knockdown cells versus CDH2 knockdown cells that have been transfected with the exogenous optimised CDH2 sequence. All experiments performed 48 hours following transfection

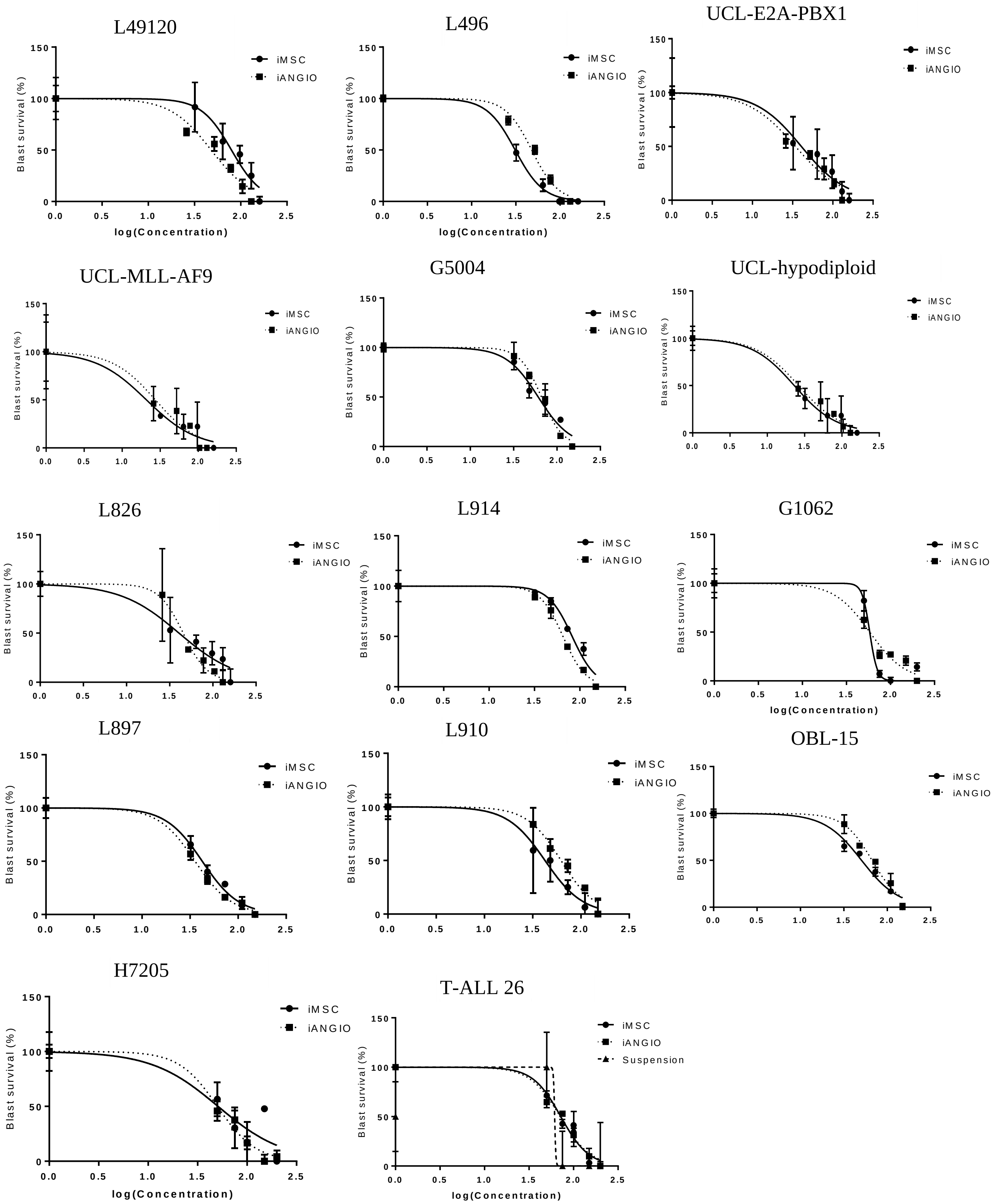

S6. Related to Fig.5. CDH2 antagonist ADH-1 a repurposed compound is identified to show high efficacy on a wide range of patient derived leukemia cells Drug dose response curves with ADH-1 on patient leukaemia samples. Data is from 2 technical repeats

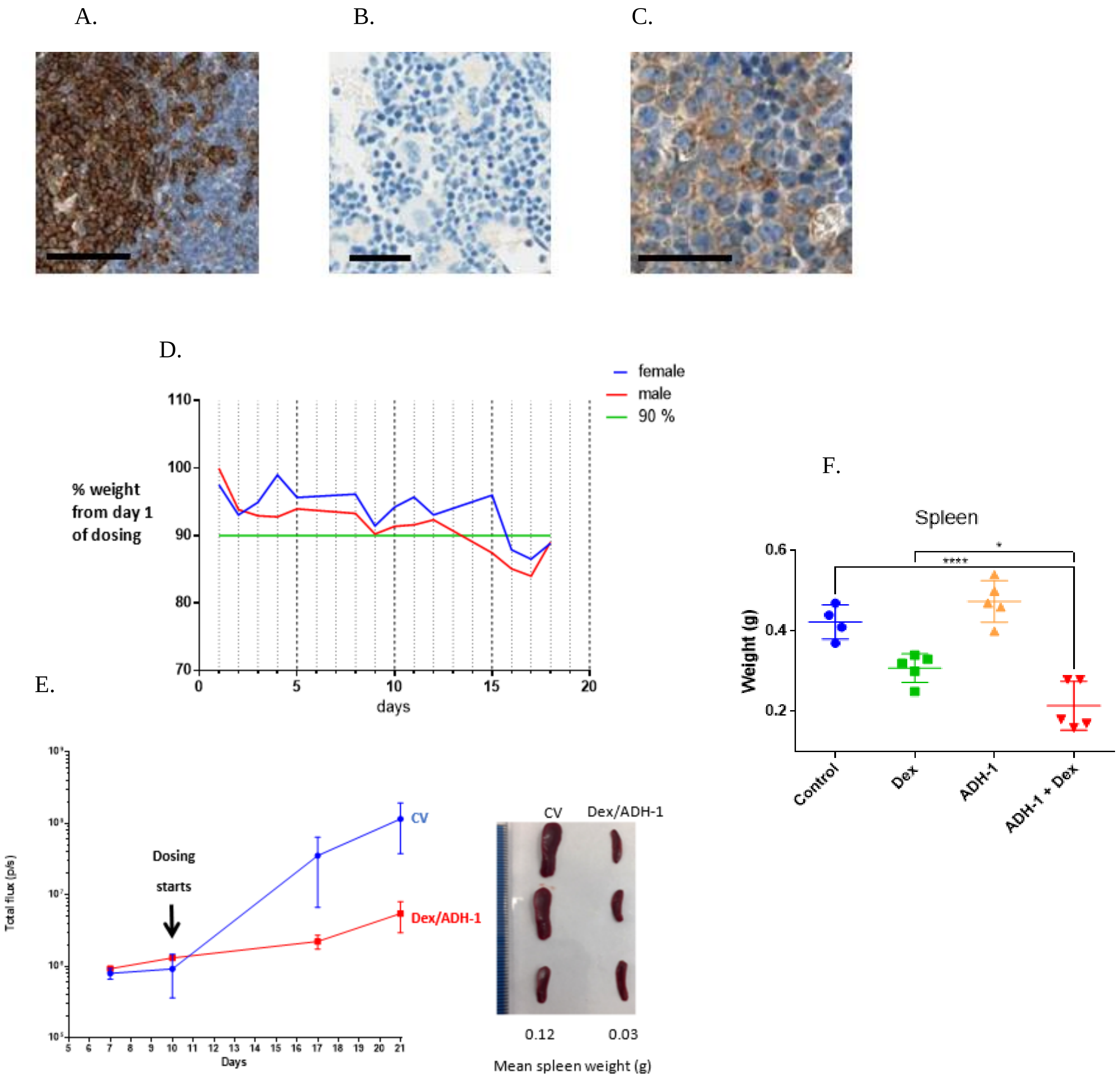

S7. Related to Fig.7. ADH-1 potentiates dexamethasone sensitivity in vivo. CD19 immunohistochemistry staining of sections from A. Human tonsil, positive control; B. Naïve, non-leukaemic mouse bone marrow, negative control, and C. Mouse bone marrow from a L707 PDX transplanted mouse at pre-treatment stage, with BLI total flux equivalent to efficacy study day6 pre-transplant. Scale bar = 50µm. D. Weights of mice (2mice/sex) administered with 3mg/kg Dexamethasone, 200mg/kg ADH-1 (Dex/ADH-1) via intraperitoneal injection, 1x daily, 5x weekly for 3 weeks. Mice appeared healthy with no clinical signs of ill health. Slight weight loss was observed consistent with single drug dexamethasone dosing. E. Dex/ADH-1 feasibility efficacy study. Mean bioluminescent imaging total flux (left) and spleens at 21 days (right) from L707D Luc+ ALL PDX mice, 3 mice/group treated with control vehicle (CV) or Dex/ADH-1 for 9 doses as for D. At 21 days the mean total flux is significantly different between CV and Dex/ADH-1 groups t-test, $p=0.002$.and spleen at 21 days are smaller. F. Spleen weights of mice treated as indicated. Lines indicate mean and SE, symbols are individual mice. 1 way ANOVA, * $p<0.05$, ****$p<0.00005$

Supplementary tables

DNA Marker	6255, BM-MSC	6255, BM-iPSC	6257, BM-MSC	6257, BM-iPSC
Amelogenin	X-X	X-X	X-X	X-X
D3S1358	16-16	16-16	14-15	14-15
THO1	9.3-9.3	9.3-9.3	07-08	07-08
D21S11	30-30	30-30	28-29	28-29
D18S51	16-16	16-16	16-18	16-18
PentaE	10-15	10-15	08-13	08-13
D5S818	12-12	12-12	11-13	11-13
D13S317	11-13	11-13	12-12	12-12
D7S820	09-11	09-11	10-10	10-10
D16S539	11-11	11-11	10-11	10-11
CSF1PO	10-10	10-10	10-12	10-12
PentaD	09-13	09-13	10-12	10-12
vWA	16-18	16-18	16-17	16-17
D8S1179	11-12	11-12	11-15	11-15
TPOX	08-11	08-11	08-11	08-11
FGA	19-20	19-20	19-25	19-25

Table S1. Related to Fig.1. Microsatellite profiling confirms the BM-iPSC are an identical match to the parental primary bone marrow mesenchymal stroma cells for the 16 microsatellites tested including amelogenin, a sex marker

Mouse number	from culture with	IVIS total flux (p/s)		Killed	spleen wt (g)	Liver wt (g)
		2 weeks	3 weeks			
1	iMSC	1.14E+06	2.26E+06	4.5 weeks	0.57	1.97
2	iMSC	1.17E+06	1.03E+06	4.5 weeks	0.52	2.18
3	iMSC	1.24E+06	1.82E+06	4.5 weeks	0.51	2.12
		Av	1.71E+06		0.53	2.09
4	iANG	1.33E+06	1.71E+06	4.5 weeks	0.35	1.81
5	iANG	6.95E+05	2.10E+06	4.5 weeks	0.32	1.86
6	iANG	1.01E+06	1.78E+06	4.5 weeks	0.37	2.09
		Av	1.86E+06		0.35	1.92

Table S2. Related to Fig. 3. B. Bioluminescent imaging total flux and organ weights of NSG mice transplanted intrafemorally with 300,000 L707D PDX cells following their culture with i-niche. Total body engraftment (measured by total flux) did not differ significantly between the two niche grown PDX however the spleens of mice transplanted with iANG grown cells were significantly smaller (measured by weight) suggesting a reduction in system engraftment of cells to the BM. The liver in this PDX model contains very few engrafted cells so is a control for mouse size.
