# Supplementary material for: hiPSC-derived bone marrow milieu identifies a clinically actionable driver of niche-mediated treatment resistance in leukemia
Source: Cell Rep Med. 2022 Aug 16;3(8):100717. doi: 10.1016/j.xcrm.2022.100717 (PMC9418860; doi:10.1016/j.xcrm.2022.100717)
Supplement: Document S2. Article plus supplemental information [file mmc2.pdf]

Cell Reports
Medicine

Article

# hiPSC-derived bone marrow milieu identifies a clinically actionable driver of niche-mediated treatment resistance in leukemia

## Graphical abstract

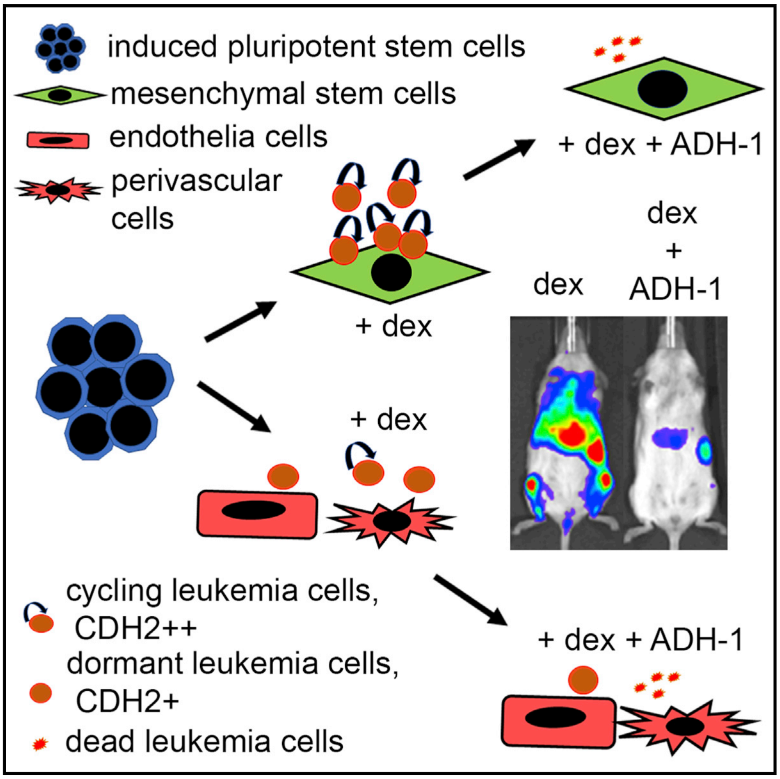


## Highlights

- Synthetic human bone marrow specifies proliferative and quiescent leukemia niches
- CDH2 mediates niche-dependent blast proliferation and treatment resistance
- Leukemia function through CDH2 is targetable via ADH-1
- ADH-1 shows high *in vivo* efficacy and low toxicity profile in aggressive leukemia

## Authors

Deepali Pal, Helen Blair, Jessica Parker, ..., Anthony V. Moorman, Olaf Heidenreich, Josef Vormoor

## Correspondence

deepali.pal@northumbria.ac.uk

## In brief

Pal et al. develop a synthetic human bone marrow milieu and identify that CDH2 mediates niche-dependent leukemia proliferation and treatment resistance. They show that CDH2 is targetable *in vivo* via ADH-1, which shows high efficacy and low toxicity in a very aggressive leukemia PDX model.

Pal et al., 2022, Cell Reports Medicine *3*, 100717
August 16, 2022 © 2022 The Author(s).
https://doi.org/10.1016/j.xcrm.2022.100717

CellPress

Cell Reports Medicine

CellPress
OPEN ACCESS

Article

# hiPSC-derived bone marrow milieu identifies a clinically actionable driver of niche-mediated treatment resistance in leukemia

Deepali Pal,[1,2,8,10,*] Helen Blair,[1,8] Jessica Parker,[2] Sean Hockney,[2] Melanie Beckett,[1] Mankaran Singh,[1] Ricky Tirtakusuma,[3] Ryan Nelson,[1] Hesta McNeill,[1] Sharon H. Angel,[1] Aaron Wilson,[1] Salem Nizami,[1] Sirintra Nakjang,[7] Peixun Zhou,[1] Claire Schwab,[1] Paul Sinclair,[1] Lisa J. Russell,[1] Jonathan Coxhead,[6] Christina Halsey,[4] James M. Allan,[1] Christine J. Harrison,[1] Anthony V. Moorman,[1] Olaf Heidenreich,[1,3,9] and Josef Vormoor[1,3,5,9]

[1]Wolfson Childhood Cancer Research Centre, Translational and Clinical Research Institute, Faculty of Medical Sciences, Newcastle University, Herschel Building Level 6, Brewery Lane, Newcastle upon Tyne, NE1 7RU UK
[2]Department of Applied Sciences, Northumbria University, Newcastle upon Tyne, NE1 8ST UK
[3]Princess Maxima Centrum for Pediatric Oncology, Heidelberglaan 25, 3584 CS Utrecht, The Netherlands
[4]Wolfson Wohl Cancer Research Centre, Institute of Cancer Sciences, College of Medical, Veterinary, and Life Sciences, University of Glasgow, Garscube Estate, Switchback Road, Bearsden, Glasgow, G61 1QH UK
[5]University Medical Center Utrecht, Heidelberglaan 100, 3584 CX Utrecht, The Netherlands
[6]Genomics Core Facility, Newcastle University, International Centre for Life, Central Parkway, Newcastle upon Tyne NE1 3BZ, UK
[7]Bioinformatics Support Unit, William Leech Building, The Medical School, Framlington Place, Newcastle upon Tyne NE2 4HH, UK
[8]These authors contributed equally
[9]Senior authors
[10]Lead contact
*Correspondence: deepali.pal@northumbria.ac.uk
https://doi.org/10.1016/j.xcrm.2022.100717

## SUMMARY

Leukemia cells re-program their microenvironment to augment blast proliferation and enhance treatment resistance. Means of clinically targeting such niche-driven treatment resistance remain ambiguous. We develop human induced pluripotent stem cell (hiPSC)-engineered niches to reveal druggable cancer-niche dependencies. We reveal that mesenchymal (iMSC) and vascular niche-like (iANG) hiPSC-derived cells support *ex vivo* proliferation of patient-derived leukemia cells, affect dormancy, and mediate treatment resistance. iMSCs protect dormant and cycling blasts against dexamethasone, while iANGs protect only dormant blasts. Leukemia proliferation and protection from dexamethasone-induced apoptosis is dependent on cancer-niche interactions mediated by CDH2. Consequently, we test CDH2 antagonist ADH-1 (previously in Phase I/II trials for solid tumors) in a very aggressive patient-derived xenograft leukemia mouse model. ADH-1 shows high *in vivo* efficacy; ADH-1/dexamethasone combination is superior to dexamethasone alone, with no ADH-1-conferred additional toxicity. These findings provide a proof-of-concept starting point to develop improved, potentially safer therapeutics targeting niche-mediated cancer dependencies in blood cancers.

## INTRODUCTION

Treatment resistance remains a major obstacle in cancer management. Emerging evidence suggests that in addition to cell-intrinsic mechanisms, factors such as the microenvironment are key in mediating cancer progression, stem cell self-renewal, and differentiation and escape from therapy.[1–7] Microenvironment conferred treatment resistance is a key impediment in treating blood cancers given that leukemic cells have a broad repertoire of tools to communicate with neighboring cells. These include direct cell-cell contact, tunneling nanotubes, exosomes and microvesicles, hormones, and other soluble messenger molecules.[8–10] In addition, leukemia cells evolve their surrounding microhabitat, and this dynamism not only enhances malignant propagation but also provides a safe haven against chemotherapy.[11,12] Leukemic cells hijack communication with bone marrow (BM) stroma and re-program their microenvironment to survive therapy.[2,12] This communication is driven by molecular programs such as IZKF1 deletions, which induce expression of adhesion molecules and mediate strong adhesion to niche cells including mesenchymal stem cells (MSC), integrin signaling, and subsequently therapy resistance.[13,14]

Means of directly drugging cell-cell contact-dependent treatment resistance with safe therapeutic agents are lacking. Key milestones in developing tractable *ex vivo* models for leukemia niche interaction show that direct contact of acute lymphoblastic leukemia (ALL) blasts with MSC in cell culture facilitates survival and modest proliferation *ex vivo*.[15–17] However, improved and


© 2022 The Author(s).
This is an open access article under the CC BY license (http://creativecommons.org/licenses/by/4.0/).

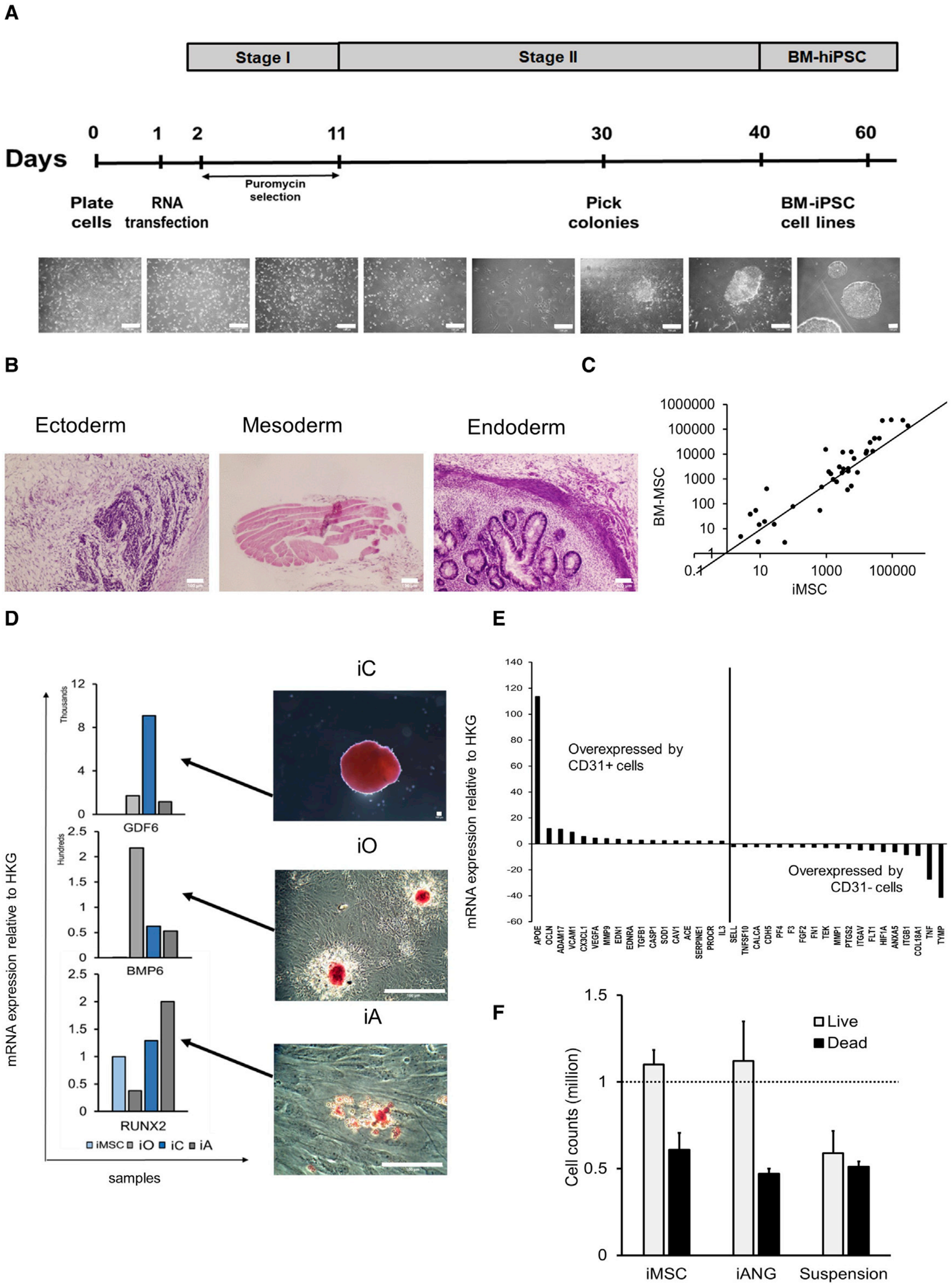


**Figure 1. BM-iPSC-derived bone marrow (BM) milieu supports human hematopoietic cells *ex vivo***

(A) Schema for synthetic RNA-based reprogramming using pluripotent transcripts POU5F1-SOX2-KLF4, GLIS1. Scale bar, 100 μM. Two BM-MSC samples (2 biological replicates) reprogrammed to form 13 BM-iPSC lines.

(B) H&E staining of BM-iPSC-derived teratomas (5 NSG mice per i-niche sample) representing the 3 embryonic lineages. Scale bar, 100 μM.

*(legend continued on next page)*

CellPress
OPEN ACCESS

experimentally accessible models are needed for in-depth scrutiny of this intricate, multicomponent, and continually evolving interaction. Using the complex BM as a paradigm, we micro-engineer human niche constituent cell types to define clinically exploitable cancer-niche interactions.

N-cadherin (CDH2) is a calcium-dependent transmembrane cell adhesion molecule known to regulate stem cell fate and proliferation.[18] The cytoplasmic domains of N-cadherin bind to β-catenin as a linker to the actin cytoskeleton, and association of N-cadherin with the cytoskeleton is necessary for the stabilization of cell-cell adhesion.[19] Cadherins play a crucial role and are a potential target in cell-cell contact of many tumor cells with their microenvironment. For chronic myeloid leukemia (CML), the N-cadherin/β-catenin complex is involved in mediating MSC-mediated resistance to tyrosine kinase inhibitors.[20] Indeed, cordycepin, an agent with limited stability *in vivo* prolongs survival in a CML cell line-derived mouse model, most likely via the suppression of CDH2.[21] Data on childhood ALL are limited, but expression of the oncogenic fusion protein E2A-PBX1 in ALL/t(1; 19) leads to the overexpression of Wnt16, which mediates the overexpression of N-cadherin and the induction of cell-cell adhesion via β-catenin.[22] The role of N-cadherin as a clinically actionable therapeutic target to disrupt malignant propagation and niche-mediated treatment response remains unexplored.

Here, we detect CDH2 as a druggable target in acute lymphoblastic and myeloid leukemia. Our study highlights the opportunity to clinically repurpose ADH-1 (Exherin), a well-tolerated drug that disrupts N-cadherin interaction. ADH-1 received orphan drug status in 2008 from the US Food and Drug Administration (FDA) and has previously been tested as an antiangiogenic agent in solid tumors in phase I/II trials.[23–25]

## RESULTS

### BM human induced pluripotent stem cell (BM-hiPSC)-derived BM milieu support human hematopoietic cells *ex vivo*

To model the human leukemia niche *ex vivo,* we re-program primary BM-MSC to pluripotency. This provides a replenishable and well-defined source of BM constituent cells that represent both the mesenchymal and vasculature niche-like cells. Sendai virus is a highly efficient approach most commonly used for pluripotent reprograming; however, there are limitations to this technique.[26] Most RNA-based approaches require repeat transfections due to reprogramming factor mRNA degradation.[27] In light of this, we adopt an RNA replicon reprogramming technology[26] that uses POU5F1, KLF4, and SOX2 in combination with GLIS1, thereby replacing MYC and consequently endorsing a reprogramming technology that is both virus-free and oncogene-free. Through standardized xeno-free protocols, we engineer 13 BM-iPSC lines (Figures 1A and S1). Microsatellite DNA fingerprinting against parental mesenchymal cells confirms authenticity (Table S1) while gene expression profiling shows upregulation of the embryonic stem cell genes SOX2, NANOG, GDF3, TERT, DNMT3B, CDH1, POU5F1, and ZFP42 (Figure S1A). BM-iPSCs exhibit a pluripotent stem cell morphology and express embryonic stem cell and pluripotency markers alkaline phosphatase, POU5F1, SOX2, SSEA4, and TRA-1-60 (Figures S1B and S1C). *In vitro* embryoid bodies (Figure S1D) and *in vivo* teratomas showing ectodermal, mesodermal, and endodermal germ layer differentiation (Figure 1B) confirm the pluripotent nature of BM-iPSC at a functional level.

Next, we derive mesenchymal (iMSC) and vascular niche-like (iANG) cells (together i-niche) from BM-iPSC through a mesoderm intermediate. We differentiate BM-iPSC into mesodermal cells through the use of Mesoderm Induction Medium. Within 72 h of initiating mesoderm induction, tightly packed pluripotent cells with a high nuclear to cytoplasmic ratio begin to alter their morphology to form cobblestone clusters comprising polygonal cells (Figure S1E). Gene expression profiling confirms the downregulation of pluripotent (Figure S1F) and the upregulation of mesodermal genes (Figure S1G), thus corroborating directed differentiation of BM-iPSC into mesodermal lineage. Furthermore, we observed the upregulation of WNT5A during this process (Figure S1H). WNT5A is observed in human embryonic stem cell-derived mesoderm,[28] and the upregulation of this gene confirms lineage-specific directed differentiation of BM-iPSC. We further differentiate these early mesoderm cells into iMSC and iANG, which show distinct transcriptomic patterns, with iMSC upregulating mesenchymal genes (Figures S2A and S2B.). We achieve iMSC differentiation by treating early mesodermal cells with mesenchymal specification media. We perform iANG differentiation experiments by treating the early mesodermal cells with vascular specification reagents such as VEGF-165 and SB431542. In addition, we show comparable gene expression profiles between iMSC and BM-MSC (Figures 1C and S2C). The ability to generate osteogenic, chondrogenic, and adipogenic cells is a gold standard test to functionally define MSCs.[29] Via differentiation of iMSC into osteogenic,

(C) Scatterplot showing comparable gene expression between primary BM mesenchymal stem cells (BM-MSC) and BM-iPSC derived MSC (iMSC). The genes profiled include MSC-specific genes IGF1, HGF, VIM, KITLG, PTPRC, PIGS, MMP2, ICAM1, COL1A1, VEGFA, TGFB3, SLC17A5, GTF3A, IL1B, NES, EGF, ITGB1, ANXA5, CSF2, CTNNB1, NUDT6, FUT1, BDNF, BGLAP, FGF22, LIF, ZFP42, SOX2, POU5F1, PROM1, CD44, MCAM, ITGA6, COL9A1, PDGFRB, NT5E, ITGAV, COL2A1, ERBB2, THY1, VCAM1, and ANPEP.

(D) GDF6, BMP6, and RUNX2 expression in i-MSC-derived cartilage/chondrocytes, bone/osteoblasts, and fat/adipocytes cells (iC, iO, and iA). Immunohistochemical staining (2 technical replicates) demonstrating safranin O, alizarin red and oil red O staining in iC, iO, and iA, respectively. Scale bar, 100 μM.

(E) mRNA expression relative to HKG (housekeeping genes: ACTB, B2M, GAPDH, HPRT1, and RPLP0) in iANG containing representative vascular cells such as $CD31^+$ endothelial cells and $CD31^-$ perivascular cells in known proportions (Figure S2).

(E) Gene expression has been normalized with respect to HKG (housekeeping genes: ACTB, B2M, GAPDH, HPRT1, and RPLP0) and the fold change expression between $CD31^+$ endothelial cells/$CD31^-$ perivascular cells has been plotted. $CD31^+$ cells express endothelial-relevant markers such as APOE, OCLN, ADAM17, and VCAM1, whereas $CD31^-$ cells express perivascular markers such as ANXA5, ITGB1, HIF1A, and COL18A1.

(F) Cell counts of $CD45^+$ hematopoietic cells (3 biological replicates) extracted from non-malignant human BM and co-cultured on iMSC, iANG versus in niche-free suspension cultures over 7 days.

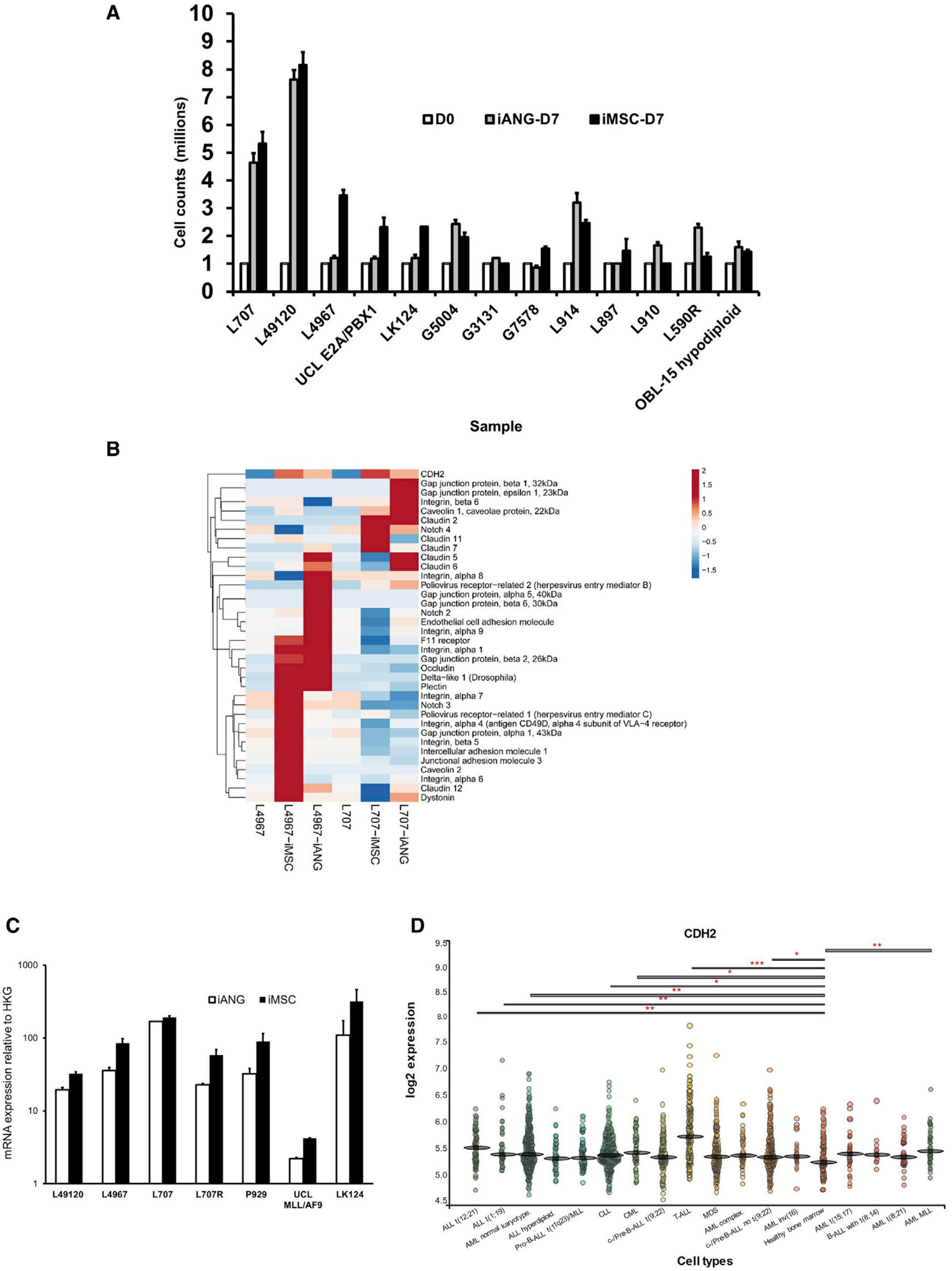


**Figure 2. Niche-primed leukemia cells upregulate CDH2**

(A) Cell counts of leukemia blasts from 13 patient-derived samples (13 biological replicates) on iMSC and iANG at diagnosis and relapse over a 7-day period.

(B) Heatmap demonstrating gene expression profiling of niche primed patient-leukemia samples (2 biological replicates: L707, L4967) shows consistent up-regulation of CDH2 following a 7-day co-culture with iMSC and iANG.

*(legend continued on next page)*

chondrogenic, and adipogenic cells (Figures 1D and S2D), we further validate their MSC potential.

iANG cells contain a population of $CD31^+$ endothelial-like cells and $CD31^-$ perivascular-like cells (Figure S2E). We show that $CD31^-$ cells express genes more closely associated with perivascular cells (Figure 1E) such as ANXA5,[30] ITGB1,[31] and HIF1A.[32] $CD31^+$ endothelial-like cells, however, upregulate the expression of genes (Figure 1E) such as APOE, which has been documented to be localized to endothelial cells *in vivo*,[33] VCAM1, an endothelial cell surface glycoprotein,[34,35] CX3CL1, known to be produced by endothelial cell membranes,[36,37] and OCLN, a functional marker of endothelial cells linked to their ability of tube formation.[38] We further show that iPSC-derived $CD31^+$ endothelial cells express key angiogenic markers and cell adhesion molecules, including HMOX1,[39] MMP2 and MMP9,[40] EDN1 and EDN2,[41,42] ANGPT1,[43] ENG,[44] VWF,[45] PDGFRA,[46] ADAM17,[47] THBS1,[48] PGF,[49] and ICAM1,[50] with ex pression levels consistent with primary human endothelia cells (Figure S2F). To specify the role of iMSC and iANG in sustaining hematopoiesis, we isolate $CD45^+$ cells from non-malignant human BM for co-culture on iMSC and iANG. Unlike microenvironment-free suspension cultures, both types of niche cells support the viability of human BM-derived hematopoietic cells (n = 3 different donors) (Figure 1F). Together, these data show that primary mesenchymal stroma stem cells reprogrammed into BM-iPSC via a virus and c-MYC-free RNA-based route are able to differentiate into MSCs and vascular BM niche-like cells. In addition, both i-niche cell types successfully support the *ex vivo* survival of human blood cells.

## Niche-primed leukemia cells upregulate CDH2

To further define the clinical relevance of i-niche cells in blood cancer, we evaluate and characterize their potential to re-create a microenvironment that would support survival, self-renewal, and the proliferation of malignant cells. We show that blasts from several patient-derived leukemia samples (n = 13 samples) proliferate on i-niche cells (Figure 2A; Key resources table). We confirm parity between iMSC and primary MSC in supporting ALL cells (Figure S3A). We show that direct niche contact is superior in supporting leukemia proliferation compared to feeder-conditioned media (Figures S3B and S3C). Using fluorescence *in situ* hybridization (FISH) analysis, we confirm that following iMSC and iANG co-culture, the leukemia cells retain their initial cytogenetic translocation (Figures S3D and S3E). We further perform whole-exome sequencing experiments to show that >99% exomes/genomic complexity remain unchanged in leukemia cells following co-culture on both iMSC and iANG (Figures S3F–S3H).

To study the effects of the different i-niche cells in supporting lymphoid and myeloid cell types we co-culture leukemia cells from a patient with infant ALL/t(4; 11) who initially presented with a $CD34^+CD19^+CD33^+CD15^-$ immunophenotype, but relapsed at 5 months with a myeloid $CD34^+CD19^-CD33^+CD15^+$ leukemia. Both iMSC and iANG support the maintenance of $CD34^+CD19^+$ lymphoid leukemic cells (lympho-permissive). In contrast, we find that some cells in suspension culture lose the expression of both CD34 and CD19. On iMSC, the leukemia blasts lose the expression of the myeloid marker $CD33^+$ (myelo-suppressive) while on iANG the blasts retain the expression of CD33 with the emergence of a population of $CD15^+$ cells (myelo-permissive) (Figures S3I and S3J; Key resources table). These data suggest that myeloid cells may be more selectively enriched on iANG compared to iMSC. However, analysis of leukemia cell proliferation and biology following co-culture with different niche types is further needed with additional biphenotypic leukemia samples.

In a previous study, we confirmed that cell-cell contact between primary BM-MSCs and leukemia cells plays a significant role in supporting the proliferation of leukemic blasts.[15] Based on the known role of adherens junctions in cell-cell contact and cancer cell-niche communication, we conduct gene expression profiling with a focus on adherens junction molecules using a combined approach of RNA sequencing, qPCR arrays, and real-time qPCR experiments on iMSC and iANG primed patient-derived blasts. Analysis of blasts following a 7-day priming (co-culture) on the i-niche cells shows the upregulation of several genes relating to adherens junction, WNT, and β-catenin pathway genes (Figure S3K). In line with our observation that niche-mediated leukemia survival and proliferation is regulated by direct cell contact, we also find the upregulation of several cell-cell junction and cell adhesion molecules on leukemia blasts co-cultured with i-niche cells over 7 days. We harvest i-niche primed blasts from co-cultures, and following cell separation through filtration, we subject the primed blasts to gene expression profiling experiments. We find consistent upregulation of cell adhesion molecule CDH2 in i-niche primed blasts across two patient leukemia samples (Figure 2B). We validate the upregulation of CDH2 on a total of six diagnostic samples and one relapse patient sample (Figure 2C). We further analyze CDH2 expression in BM from healthy individuals versus patients with leukemia using data from the MILE study[51] via the database BloodSpot (www.bloodspot.eu).[52] We find significant overexpression of CDH2 across 8 different leukemia types—ALL t(12; 21), ALL t(1; 19), AML normal karyotype, CLL, CML, T-ALL, c-/Pre-B-ALL no t(9; 22), and AML MLL (Figure 2D). In summary, these data show that BM-iPSC derived niche cell types support leukemia cells, and leukemia blasts primed by the i-niche cells upregulate CDH2 expression.

## Under dexamethasone treatment, CDH2 is upregulated by iMSC-primed cycling cells

We study the role of CDH2 in niche-mediated cancer cell quiescence and proliferation. DNA labeling dyes allow the isolation and tracking of dormant cells identified as the non-/slow-dividing and label-retaining population.[53] We perform cell generational tracing experiments to compare patterns of leukemia dormancy between the mesenchymal and vascular niche-like microenvironments. A patient with ALL/t(17; 19) (Key resources table)

(C) CDH2 upregulation confirmed by qRT-PCR on 7 leukemia samples (7 biological replicates, each biological replicate has 3 technical replicates) following a 7-day co-culture with iMSC and iANG.

(D) Gene expression profiles from BloodSpot database, MILE study showing CDH2 expression levels between healthy and leukemic BM.

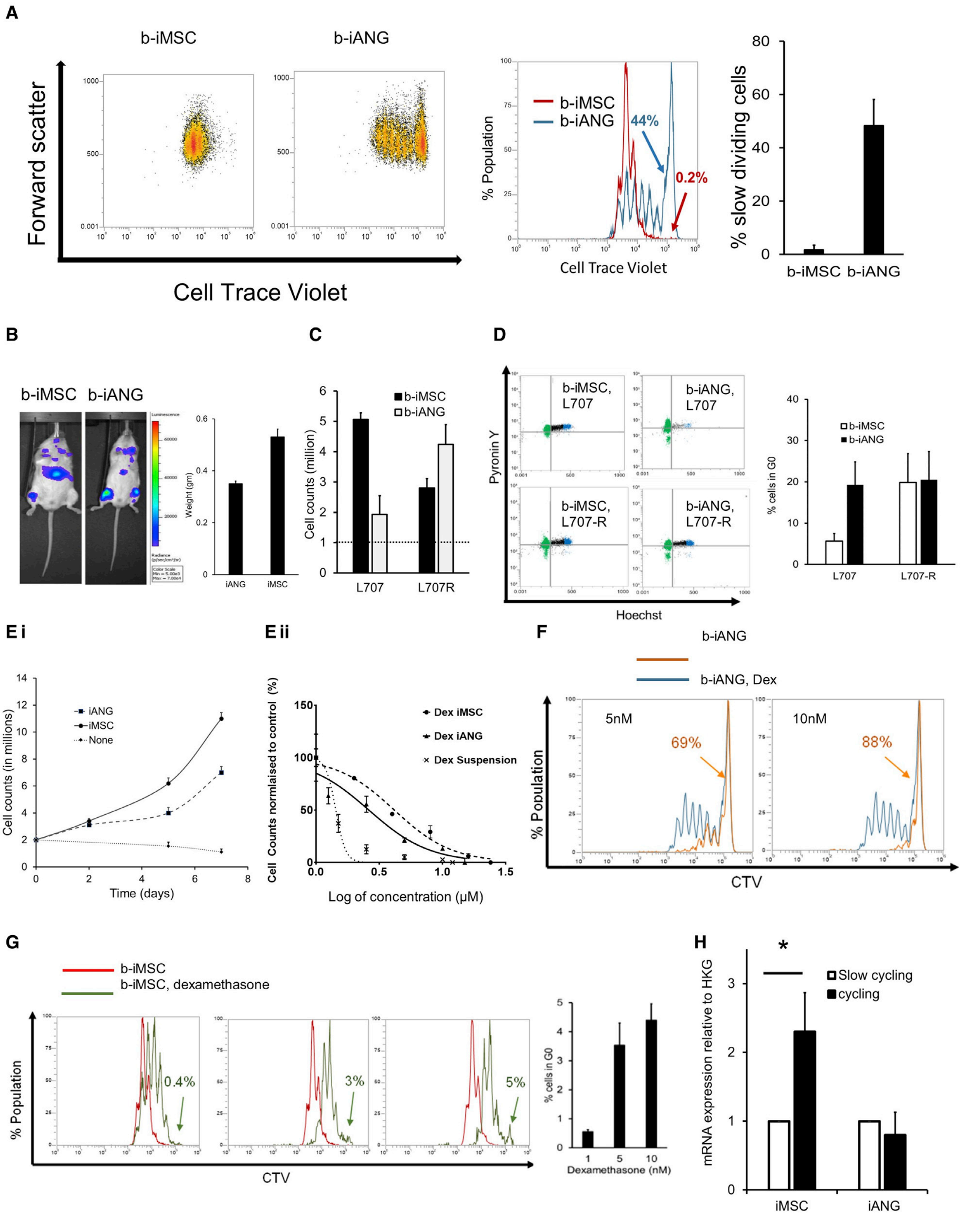


*(legend on next page)*

who initially presented with dexamethasone-sensitive leukemia but later relapsed with steroid-resistant disease (due to a homozygous deletion of the glucocorticoid receptor NR3C1) was used as a model to study the effects of dexamethasone in our i-niche system.

We detect distinct patterns of leukemia quiescence and proliferation on the two niche cell types (Figure 3A.). iMSC exclusively support fast-dividing blasts (label$^{low}$). In contrast, nearly 50% of the total patient-derived blasts on iANG cells are non-dividing cells (label$^{high}$). Both iMSC- and iANG-primed blasts engrafted immunocompromised mice, although iANG-primed blasts appear to preferentially home to the murine BM and to a lesser degree to the spleen (Figure 3B; Table S2). To further define the role of the different i-niche cells on leukemic quiescence and proliferation, we extend our analysis to include cells from the matched relapse sample (Figures 3C and 3D). We find that cells from the diagnostic sample proliferates faster on iMSC, while the relapse cells proliferate faster on iANG cells (Figure 3C). Hoechst/Pyronin Y cell-cycle staining experiments show a four-fold higher percentage of cells from the diagnostic sample in G0 on iANG niche cells (Figure 3D). We find no difference in the percentage of cells from the relapse sample in Gene Ontology (GO) or in the cell-cycling pattern when cultured on either iMSC or i-ANG (Figures 3D and S4B).

To study niche-mediated resistance, we repeat the cell generational tracing experiments under dexamethasone treatment pressure. Dose-response curves demonstrate reduced sensitivity against dexamethasone on both types of i-niche cells as compared with the niche-free suspension cultures (Figure 3E). On iANG cells, dexamethasone treatment actively kills the dividing blasts, mainly leaving a non-dividing label$^{high}$ population intact and in a non-proliferating state (Figures 3F and S4B). On iMSC, treatment causes the cell division curve to shift to the right, identifying cell populations that are dividing more slowly and with the emergence of only a small (5%) non-dividing population (label$^{high}$); the remaining 95% of cells are proliferating, although at a rate much lower than the untreated cells (Figures 3G and S4B). Unlike iANG cells, iMSC cells facilitate the survival of slower dividing ALL blasts under dexamethasone treatment, suggesting that treatment resistance is unlikely to be attributed to dormancy alone. To put these data into context, we validate clinically proven drug responses in our *ex vivo* model and confirm that in compliance with molecular and clinical data, cells from the diagnostic sample are sensitive to dexamethasone, while relapse blasts show no response (Figure S4C). Consequently, we re-visited the role of CDH2 in proliferation and treatment resistance. We detect that fast dividing, label$^{low}$ iMSC-primed blasts in four patient samples that survive under dexamethasone pressure express higher levels of CDH2 (Figures 3H and S4D). These data suggest that CDH2 plays a direct role in mediating niche-dependent leukemia proliferation in blasts that are resistant to treatment with dexamethasone.

## CDH2 drives leukemia proliferation and reduces sensitivity against dexamethasone

To validate the function of CDH2, we performed RNAi knockdown experiments on both cancer cells and i-niche cells. Small hairpin RNA (shRNA) CDH2 knockdown in four different leukemia cell lines (Figures 4A and S5A) result in reduced proliferation in niche-free suspension cultures (Figures 4B and 4C). Moreover, CDH2 knockdown results in the downregulation of a range of cancer-associated gene signatures (Figures S5B and S5C), including key oncogenic pathways, such as Janus kinase-signal transducer and activator of transcription (JAK-STAT), prolactin, chemokine, and ErbB signaling, as well as the modulation of several genes associated with leukemogenesis and transcription and chromatin remodeling factors (Figures S5C–S5E). We further validate these knockdown experiments by showing that leukemia cell proliferation is restored when CDH2 expression is rescued by overexpressing an exogenous optimized CDH2 sequence[54] in the knockdown cells (Figures S5F–S5H).

We find that CDH2 knockdown leukemia cells, co-cultured under modified culture conditions to facilitate niche dependence, fail to survive on iMSC cells and show reduced proliferation on iANG (Figure 4D). We conduct additional validation experiments to knockdown CDH2 in iMSC using a second shRNA independent of the one used above. CDH2 knockdown in iMSC (Figure 4E) reduce their ability to support the proliferation of patient-derived leukemia samples, three diagnostic and one relapse samples (Figure 4F). In addition, the leukemia cells

**Figure 3. Under dexamethasone treatment pressure, CDH2 is upregulated by iMSC-primed cycling cells**

(A) Dot plots show fast cycling and slow cycling iMSC primed blasts (b-iMSC, red) and iANG primed blasts (b-iANG, blue) at day 7 from a patient-leukemia (L707) sample at diagnosis. Histogram overlay and graph shows the percentage of slow cycling blasts on iMSC and iANG. Data shown from 2 technical replicates.

(B) Total fluorescence intensity of luciferase-tagged niche-primed patient leukemic blasts transplanted in immunocompromised mice. The column graph depicts spleen weights (harvest at 4.5 weeks following injection) in mice transplanted with patient blasts (L707) at diagnosis (control) and following a 7-day co-culture on iMSC (b-iMSC) and iANG (b-iANG). Intrafemoral transplants, n = 3 mice, 1 representative example shown.

(C) Cell counts of a diagnostic and matched relapse sample following co-culture on iMSC and iANG. Three technical replicates.

(D) Hoechst-pyronin Y analysis (dot plot) of patient leukemic blasts on iMSC (left panel) and iANG (right panel) in patient leukemic blasts at diagnosis (top panel) and relapse (bottom panel). Graph shows percentage cells in G0 on iMSC (b-iMSC) and on iANG (b-iANG) at diagnosis (L707) and relapse (L707-R). Two technical replicates.

(E) (i) Growth curve showing proliferation of patient leukemia cells (L707, 3 technical replicates) over a 7-day period on iMSC and iANG. (ii) Dexamethasone dose response (nM) curve of patient leukemia cells (L707, 3 technical replicates) treated for 7 days in niche-free suspension culture and on iMSC and iANG.

(F) Histogram shows cell generational curve of untreated (blue) and treated leukemia cells (orange) co-cultured on iANG over a 7-day period. Two technical replicates, 1 representative example shown.

(G) Cell generation curves of patient leukemic cells untreated (red) and treated (green) when co-cultured on iMSC over a 7-day period. Column graph shows percentage of slow cycling blasts on iMSC under dexamethasone treatment. Two technical replicates.

(H) CDH2 expression under dexamethasone pressure in slow cycling and cycling/fast cycling blasts relative to HKG (GAPDH). Blasts were sorted using flow cytometry following 7-day treatment with 5 nM dexamethasone. Three technical replicates for 1 patient sample, L707 shown here. *Unpaired t test shows $p < 0.05$. Data for 3 additional patient samples/biological replicates, with each containing 3 technical replicates, are included in Figure S4E.

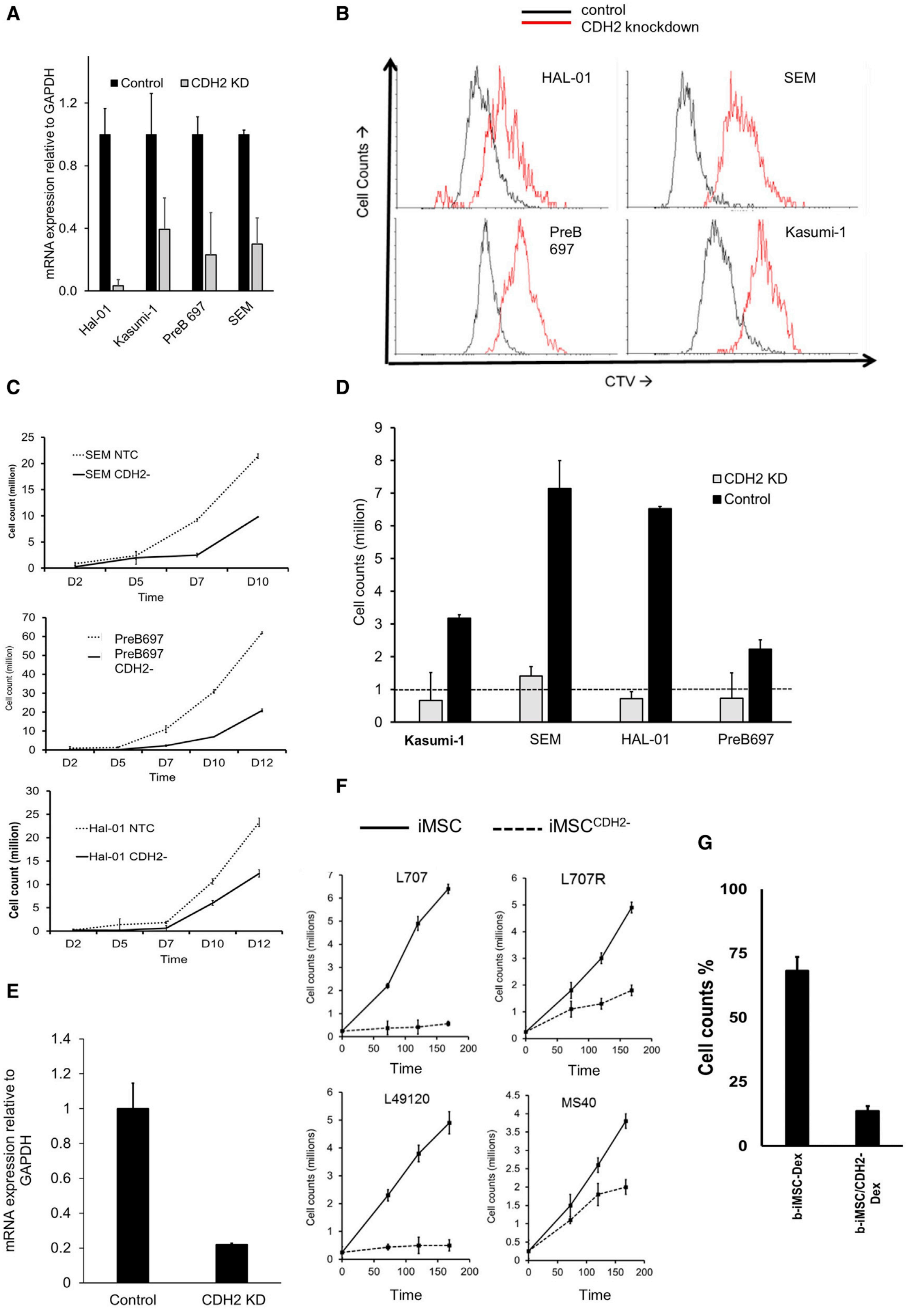


(legend on next page)

show three-fold higher sensitivity to Dexamethasone on iMSC$^{CDH2-}$ cells (Figure 4G). These data suggest that BM MSCs mediate their leukemia supportive effect via heterologous cancer-niche interactions through CDH2-CDH2 binding and signaling.

### CDH2 antagonist ADH-1 shows high *in vitro* efficacy in patient-derived leukemia cells

ADH-1 is a small, cyclic pentapeptide with the formula N-Ac-CHAVC-NH2 that competitively blocks the action of CDH2. In preclinical models it has antiangiogenic properties in disrupting tumor vasculature and inhibiting tumor growth. ADH-1 has been in Phase I/II trials for advanced solid malignancies[23–25] and received orphan drug status from the FDA in 2008, although its efficacy in blood cancers remains unknown. First, we show that ADH-1 fails to show a drug dose response in ALL and AML cells that have been transduced with shRNA to knockdown CDH2 (Figure 5A.). Second, we apply our i-niche co-culture platform and demonstrate sensitivity to ADH-1 in 15 different patient-derived leukemia samples (Figures 5B and S6; Key resources table). ADH-1 doses used throughout this study are consistent with plasma level concentrations that have been achieved in solid tumor trials.[55] We find that ADH-1 treatment shows maximum efficacy when the leukemia cells are in direct contact with the niche as opposed to transwell cultures (Figures 5C and 5D). Interestingly, it has been documented that CDH2 antagonist ADH-1 causes apoptosis in pancreatic cancer cells, even though cell adhesion in this case is mediated by E-cadherin (CDH1) and this CDH1-mediated cell adhesion is not disrupted by ADH-1.[56] We also observe ADH-1 induced leukemia cell toxicity both in suspension and on co-cultures, which further substantiates the role of the niche cells as well as homotypic leukemia cell-cell contact affecting leukemia cell survival and proliferation. In combination with our findings, this further suggests that CDH2-mediated cell survival may not be regulated exclusively by cell adhesion and additional mechanisms may be involved. In addition, we show ADH-1 treatment in cancer-niche co-cultures increases leukemia cell death as evidenced by increased annexin V and propidium iodide (PI) staining (Figure 5E).

To further investigate the effect of this compound on cells that survive under treatment pressure, we performed live cell-cycle and G0 analysis on patient-derived leukemia cells at relapse. We find that ADH-1 treatment slows down the proliferation of the leukemia cells as evidenced by the reduced number of blasts in S phase. Furthermore, there is no accumulation of cells in G0 (Figure 5F). Taken together, these data suggest that ADH-1 is potentially a promising candidate for the treatment of high-risk, treatment-resistant cases in which it would be unlikely to induce the emergence of resistant quiescent cells.

Despite the recent improvements in targeted therapies, single-agent treatment is associated with the emergence of treatment-resistant cancer clones.[57,58] Combinatorial drug treatment is a central principle in anticancer therapy not only to enhance efficacy through drug synergies but, most important, to prevent the emergence of treatment resistance. Drug combination assays with dexamethasone and ADH-1 using four different patient-derived leukemia samples (Figures 6A–6F) show synergistic interaction as analyzed by the Bliss independence model. Comprehensive drug matrix analyses (Figures 6G and 6H) demonstrate synergy for ADH-1 in combination with clinically relevant concentrations of dexamethasone achieving ZIP synergy scores of >10 on both iMSC and iANG. Taken together, these data reveal a means of clinically targeting niche-mediated leukemia treatment resistance using the CDH2 antagonist ADH-1.

### ADH-1 shows high *in vivo* efficacy in a very aggressive leukemia patient derived xenograft (PDX) model

To validate the function of CDH2 *in vivo*, we explored its role in leukemia initiation and propagation in our PDX model.[59–61] We transplanted luciferase-tagged leukemia blasts from a clinical sample of very-high-risk ALL (L707; Key resources table) directly into the BM of immunodeficient mice. We monitored leukemia engraftment into the mouse BM via bioluminescence and confirmed successful engraftment through immunohistochemistry staining of mouse BM with human CD19, a lymphoid cell marker (Figures S7A–S7C). In an initial pilot study, we treated mice with a combination of ADH-1 and dexamethasone to determine a non-toxic dose and schedule for further study (Figure S7D). We found that this small-scale dose-escalation pilot study indicated that ADH-1 and dexamethasone in combination significantly reduced leukemic engraftment (Figure S7E), justifying further *in vivo* investigation of the combination treatment. We also found that ADH-1/dexamethasone (ADH-1 200 mg/kg; dexamethasone 3 mg/kg) delivered via intraperitoneal injection is well tolerated when administered 5 times weekly for 3 weeks with minimal weight loss. We based ADH-1 dosing on previously published studies in mice[56,62] and we chose

**Figure 4. CDH2 drives leukemia proliferation and reduces sensitivity against dexamethasone**

(A) CDH2 levels in leukemia cell lines following lentiviral knockdown. Control = nonsense shRNA/non-targeting control. Four biological replicates, with each containing 3 technical replicates.

(B) Cell generational tracing curves using the dye cell trace violet (CTV) in 4 different leukemia cell lines following CDH2 knockdown. Black = empty vector control. Red = CDH2 knockdown. Four biological replicates, with each containing 3 technical replicates. One representative example is shown here.

(C) Leukemia cell proliferation in 3 different acute lymphoblastic leukemia cell lines following CDH2 knockdown (against empty vector control). Three biological replicates, with each containing 2 technical replicates.

(D) Cell counts of CDH2 knockdown and empty vector control cell lines on iANG over 5 days. Dashed line indicates a starting cell count of 1 million cells. Feeder dependence was achieved by conducting co-cultures in the absence of fetal bovine serum (FBS) and at a reduced leukemia cell density of 10,000 cells/mL. Under these altered culture conditions, the leukemia cells failed to survive on iMSC. Four biological replicates, with each containing 2 technical replicates.

(E) CDH2 mRNA levels in control iMSC and CDH2 knockdown iMSC (iMSC$^{CDH2-}$). Two technical replicates.

(F) Cell counts of 3 different patient leukemia samples. Three biological replicates and 1 matched relapse sample on iMSC (solid line) and iMSC$^{CDH2-}$ (dotted line).

(G) Percentage of cell counts (with respect to untreated control) of patient leukemia cells (L707) on iMSC$^{CDH2-}$ with and without 5 nM dexamethasone. One biological replicate, 3 technical replicates.

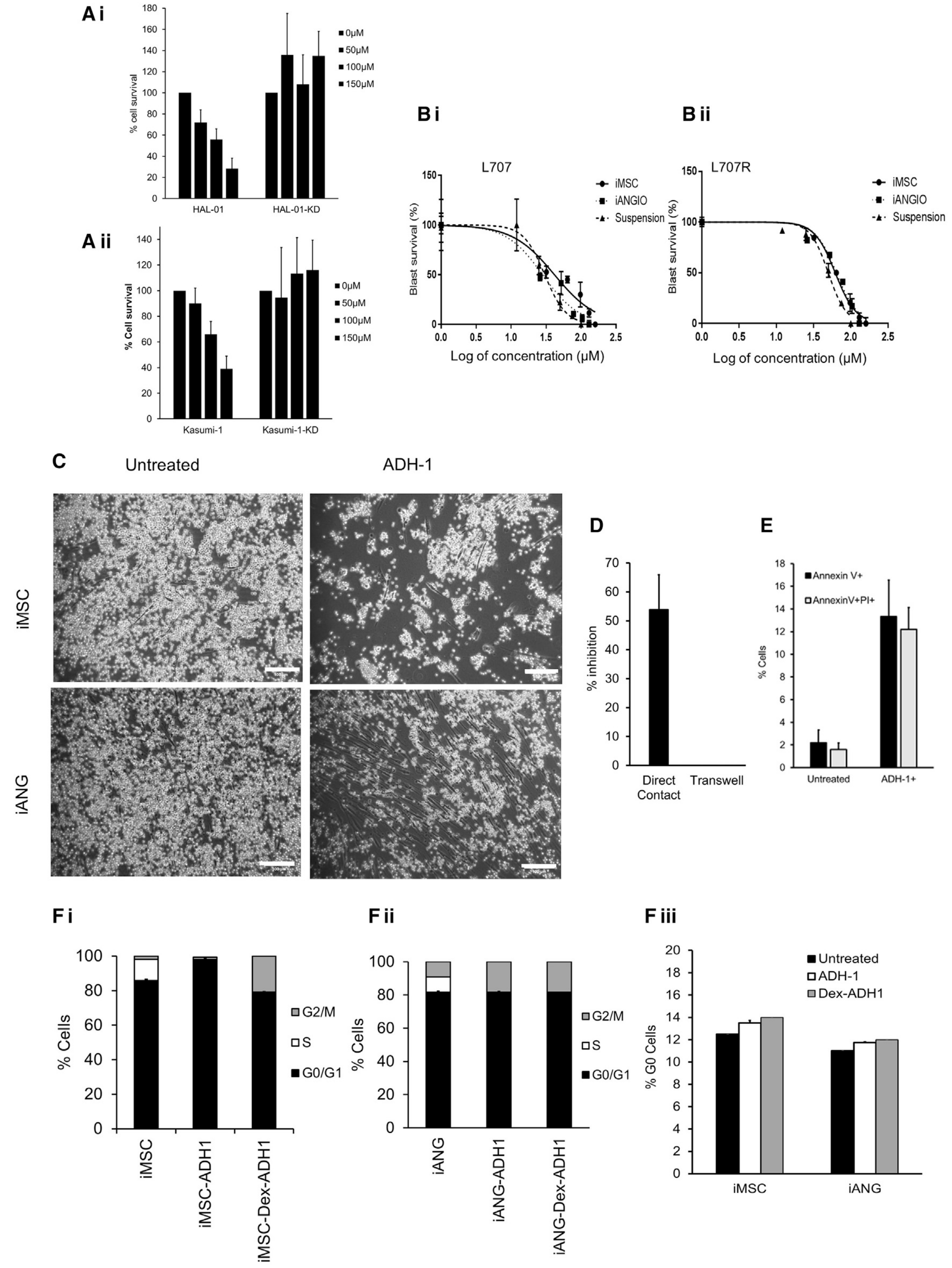


**Figure 5. CDH2 antagonist ADH-1, a repurposed compound, is identified to show high efficacy on a wide range of patient-derived leukemia cells**
(A) ADH-1 treatment on CDH2 knockdown and control (i) ALL and (ii) AML leukemia cells. Two biological replicates, with each containing 3 technical replicates.
(B) ADH-1 dose-response curves in patient leukemia samples from a patient at (i) diagnosis and (ii) relapse. Doses used are in the range 12–450 μM, which is consistent with $C_{max}$ levels achieved in solid tumor clinical trials. The x axis of the graphs shows log of concentration of ADH-1 used. One biological replicate shown here. A total of 14 additional biological replicates shown in Figure S6. Each biological replicate contains 2 technical replicates.

(legend continued on next page)

dexamethasone dose to replicate plasma concentrations achieved in ALL patients.[55,63,64] We repeated the *in vivo* transplantation experiments using bioluminescent-tagged patient-derived ALL blasts and started drug dosing on day 6 following transplantation (Figure 7A). By bioluminescence monitoring, ADH-1 alone shows a similar reduction in leukemia progression as observed in mice treated with dexamethasone. More important, the ADH-1/dexamethasone combination treatment profoundly reduces leukemia engraftment compared to controls and single-agent therapy. Through additional bioluminescent imaging (BLI), we demonstrate significantly lower overall signals compared to untreated controls at both weeks 2 and 3 of ADH-1/dexamethasone therapy (Figures 7B and 7C). Confirming the imaging data, spleen sizes are significantly smaller in the ADH-1/dexamethasone-treated mice at the end of the study. We further show that the proportion of leukemia blasts in BM and spleen is significantly less in ADH-1/dexamethasone-treated mice compared with mice from the dexamethasone and control groups (Figures 7D, 7E, and S7F). In keeping with our *in vitro* observations, the ADH-1/dexamethasone combination is most effective in the BM (Figures 7D and 7E), suggesting that a key mechanism of action for ADH-1 is to disrupt CDH2-mediated blast-BM niche interactions, consequently increasing sensitivity to dexamethasone.

In summary, we find that treatment with only 15 doses of dexamethasone in the presence of ADH-1 is more effective than dexamethasone alone at blocking leukemia growth *in vivo*. This *in vivo* efficacy validates the use of our engineered preclinical model for future identification of further clinically exploitable niche targets.

## DISCUSSION

Treatment resistance and treatment toxicity are major clinical challenges that urgently need resolving. Dynamism of the leukemic niche and its role in dormancy and treatment resistance is well known.[2,53,65–67] Standard chemotherapy primes cancer and its ambiance alike, endowing cell intrinsic and non-cell-autonomous adaptations toward treatment resistance.[2,68] Recent concepts such as non-oncogene addiction,[57] a phenomenon underpinning cancer cell survival through exaggerated functioning of non-mutated genes have emerged as a promising solution to prevent treatment resistance. Despite the significant impacts of the niche on cancer cell function, no druggable niche targets exist that can directly affect microenvironment-mediated leukemia biology. Present-day treatment largely disregards the influence of the oncogenic microenvironment on malignant proliferation, self-renewal, and treatment resistance.

To identify safer actionable targets against the leukemic niche, we require improved preclinical models. This is a major challenge in hematological malignancies since most primary leukemia cells do not proliferate once removed from the patient and his or her microenvironment. Consequently, there is a lack of models that allow scrutiny of the niche in a human cell-based setting.[15,69] Here, we show that hiPSC-engineered BM cells support *ex vivo* proliferation of patient-derived leukemia cells. Furthermore, different niche cell types can be derived that specify both proliferative and quiescent niches for leukemia.

Cadherins are cell adhesion molecules that enable cells to communicate with their environment, and cell-cell contact promotes cell survival.[70] The role of CDH2 in directing stem-cell fate, tumor-microenvironment interactions, and chemoresistance has been implicated in a wide variety of solid tumors and certain hematological malignancies such as CML.[71,72] However, clinically relevant data on the role of CDH2 in microenvironment-mediated cancer proliferation and therapy resistance in acute leukemia have until now been scarce, as has the means of clinically targeting this via low toxic agents. CDH2 binding and signaling is essential in mediating contact of the leukemia blasts with niche cells. CDH2 is upregulated upon heterologous cell contact of the leukemic cells with iMSC; in contrast, CDH2 knockdown in either leukemia blasts or iMSC disrupts this interaction, impairs blast survival and proliferation, and leads to the downregulation of key oncogenic pathways (e.g., JAK/STAT signaling).

We detect that CDH2 mediates niche-mediated therapy resistance. Heterologous contact of the leukemia cells in these co-culture conditions induces upregulated expression of CDH2, and knockdown of CDH2 in iMSC increases the sensitivity of patient-derived blasts. iMSC and vasculature niche-type iANG cells support leukemia survival under treatment in different ways. Dexamethasone treatment of patient-derived ALL cells on iANG primarily leads to the selection and survival of blasts in G0. On iMSC, while there is still the emergence of a small resting cancer cell population under treatment pressure with dexamethasone, the majority of surviving blasts continue to cycle. These cycling blasts express even higher levels of CDH2. Therefore, although a key feature of treatment resistance remains dormant, not all treatment-resistant cells are dormant, and other mechanisms warrant further attention. Specifically, emerging evidence has attributed a senescent-like albeit reversible state to emerge following chemotherapy and to be associated with treatment resistance and relapse.[73] Such senescent-like phenotypes have been further attributed to acquisition of "stemness" consequently leading to oncogenic transformation.[74]

Cadherin binding is mediated via a unique amino acid motif that flanks hepatitis A virus (HAV) sequences in the extracellular domain and is blocked by the small cyclic peptide, ADH-1, composed of the unique CDH2 amino acid-binding motif.[75] ADH-1 disrupts both CDH2 cell adhesion in a dose-dependent manner and neurite outgrowth with a half-maximal inhibitory concentration ($IC_{50}$) of 320 μM.[76] In preclinical mouse xenograft studies, a 50-mg/kg dose of ADH-1 significantly reduces

(C) Adherent patient blasts (L707) on iMSC and iANG following treatment with 50 μM ADH-1. Scale bar, 100 μm. Three technical replicates.
(D) Percentage of inhibition (cell counts) of blasts (L707) following 50 μM ADH-1 treatment on direct contact cultures (iMSC) and in transwell cultures. Three technical replicates.
(E) Annexin V PI flow cytometry analysis in patient blasts (L707) following treatment with 50 μM ADH-1. Two technical replicates.
(F) (i–iii) RNA and DNA content analysis using flow cytometry in primary blasts (L707) following treatment with 50 μM ADH-1 in (i) iMSC and (ii) iANG co-cultures. (iii) Percentage of G0 cells in co-cultures following treatment with ADH-1. Two technical replicates.

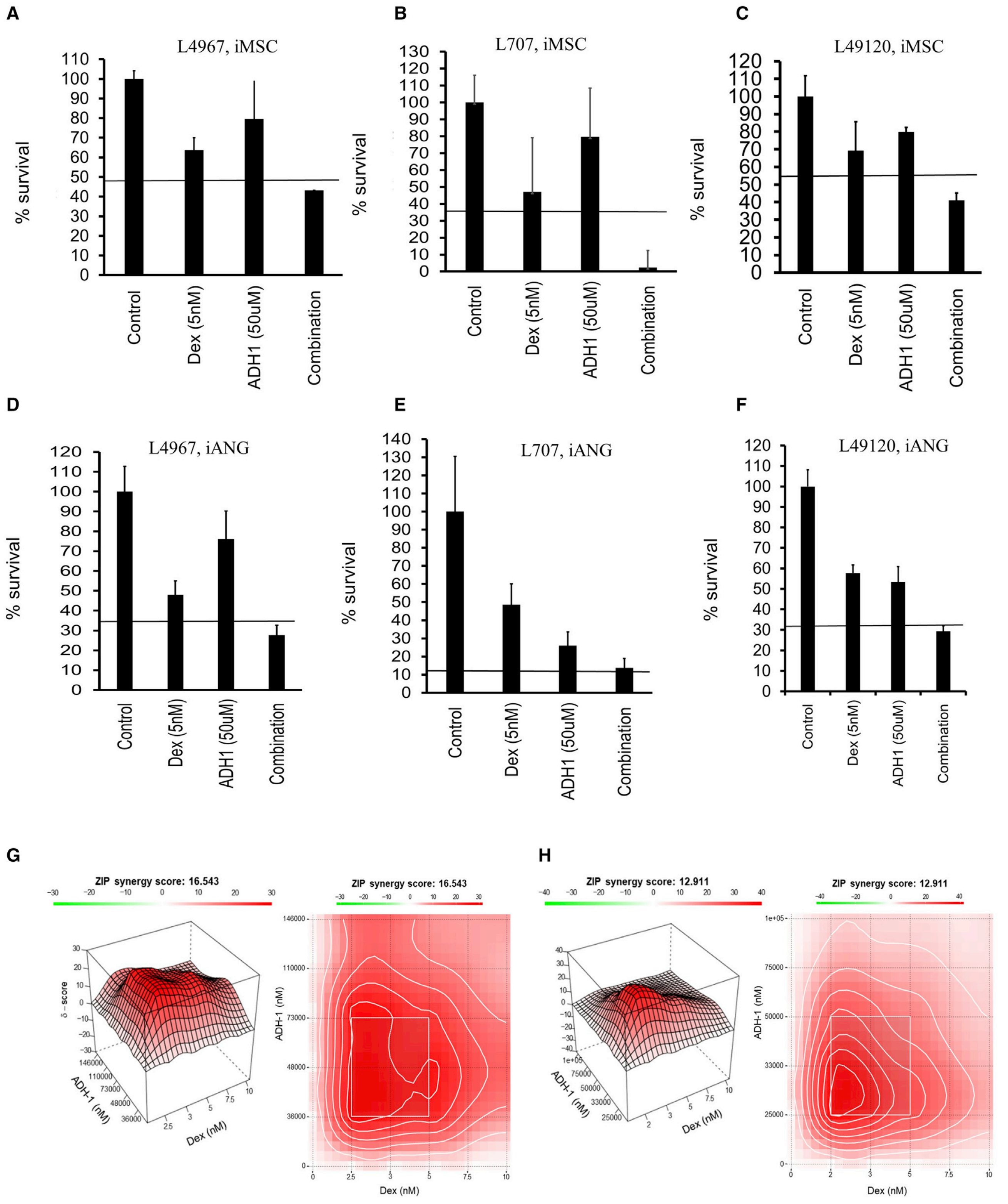


(legend on next page)

pancreatic cell survival and invasion.[56] In addition, 100 mg/kg/day ADH-1 *in vivo* enhances chemotherapeutic efficacy in CDH2-expressing melanoma xenografts but not in CDH2$^-$melanoma xenografts, demonstrating the specificity of ADH-1 to inhibit CDH2.[77] Specificity of ADH-1 to CDH2 has also been observed in clinical trials with increased efficacy in patients with CDH2-expressing tumors compared to CDH2$^-$ tumors.[55] Pharmacokinetic studies in humans indicate a relatively short half-life of ADH-1 in plasma and a low toxicity profile at all ADH-1 doses administered.[23–25] Here, we reveal that clinically relevant concentrations of ADH-1[24] show high efficacy against a panel of 15 patient-derived leukemia samples.

Using a xenograft mouse model of a highly aggressive incurable leukemia we find that ADH-1 efficacy is similar to that of dexamethasone alone. We show that combination treatment with ADH-1 and dexamethasone is more efficient than dexamethasone alone and that the addition of ADH-1 does not confer any additional toxicity. ADH-1 has been explored as an antiangiogenic drug in early phase clinical trials in solid tumors and published data indicate a tolerable clinical toxicity profile.[23–25,55] ADH-1 may, therefore, be a candidate for clinical repurposing or a good starting point for a drug-discovery program to meaningfully target the niche in blood cancers.

Using the complex hematopoietic BM niche as a paradigm, our proof-of-concept preclinical platform provides a prototype that can be adapted to investigate malignant niches in a wide variety of hematological cancers. As an example of proof of confidence in application, our findings highlight the role of N-cadherin signaling in microenvironment-mediated drug resistance in leukemia. This provides a starting point for the development of safer and more efficacious therapies to clinically target the tumor microenvironment.

## Limitations of the study

Here, we develop a simplified prototype model to detect cancer-niche cellular interactions mediating treatment resistance. We use the BM as a paradigm to study leukemia. Further development of the BM model is warranted to first decode the cellular complexity of the BM in health, aging, and disease and consequently replicate the diverse cellular components with spatial mimicry in an organoid format. Furthermore, such multicellular culture systems require advanced technologies to aid in characterization and validation; these include but are not limited to *in situ* transcriptomic profiling.

We observe that ADH-1 treatment is not effective in transwell cultures highlighting the requirement for direct cell-niche contact for ADH-1 to be effective. However, we also note effective ADH-1 action when cells are in suspension by themselves. These findings may be due to an intricate interplay between cell adhesion molecules and secreted factors. Such crosstalk between cell adhesion molecules, growth factors, and cell surface receptors is expected in multicellular environments due to autocrine and paracrine cell-cell signaling.[78,79] Given the immunogenic role of MSCs,[80] such intercellular communications could also be immune regulated. Mechanisms underpinning interaction between cell contact molecules and cell-secreted factors, including immune regulation of cancer-niche interactions, warrant further investigation in future studies.

## STAR★METHODS

Detailed methods are provided in the online version of this paper and include the following:

- KEY RESOURCES TABLE
- RESOURCE AVAILABILITY
  - Lead contact
  - Materials availability
  - Data and code availability
- EXPERIMENTAL MODEL AND SUBJECT DETAILS
  - Ethical approval
- METHOD DETAILS
  - Study design
  - Functional *in vitro* knockdown, iMSC
  - Mouse transplantation studies
- QUANTIFICATION AND STATISTICAL ANALYSIS
  - Statistics

## SUPPLEMENTAL INFORMATION

Supplemental information can be found online at https://doi.org/10.1016/j.xcrm.2022.100717.

## ACKNOWLEDGMENTS

This study was funded by NC3Rs grants to D.P.; a CCLG project grant to D.P., J.V., and O.H.; and a Wellcome Trust NUSCU award to D.P. We would like to further thank CRUK for program grant no. C27943/A12788 to J.V. and O.H. and NECCR for funding core infrastructure at Wolfson Childhood Cancer Research Centre, Newcastle University. C.J.H. and A.V.M. were funded by Blood Cancer UK (grant no. 15036). The IVIS Spectrum was funded by grant 087961 from the Wellcome Trust. We thank Dr. Malgorzata Firczuk, Medical University of Warsaw, for the PDX samples. We thank Sophie Boyd, Asmida Isa, and Shalini Shankar for technical assistance. We thank the BSU, Newcastle University, for performing the bioinformatics analysis; and Andrew Filby, Andrew Fuller, and Carly Knill at the Flow Cytometry Core Facility, Newcastle University, for assistance with flow cytometry. The study was also funded by the National Centre for the Replacement Refinement and Reduction of Animals in Research (NC3Rs) (NC/P002412/1 and NC/V001639/1, to D.P.), the Children's Cancer and Leukaemia Group (CCLG) (CCLGA 2016 05 BH160568, to D.P., J.V., and O.H.), Wellcome Trust NUSCU award (OSR/0190/DPAL/NUSC [to D.P.]), Cancer Research UK (CRUK) grant no. C27943/A12788 (to J.V. and O.H.), and Blood Cancer UK grant no. 15036 (to C.J.H. and A.V.M.).

**Figure 6. ADH-1 demonstrates *in vitro* synergy in combination with dexamethasone**

(A–C) Percentage of survival following treatment with dexamethasone, ADH-1, and combination in 3 different patient samples over 7 days. (A) L4967, (B) L707, and (C) L49120 on iMSC, and (D–F) on iANG. Horizontal line depicts the expected combined effect as per the Bliss independence model. Three technical replicates.

(G) Synergy landscapes (3-dimensional [3D] and 2D synergy maps) and ZIP synergy scores of Dex/ADH-1 on patient-derived blasts (L707) on iMSC.

(H) iANG co-cultures over a 7-day period. Two technical replicates.

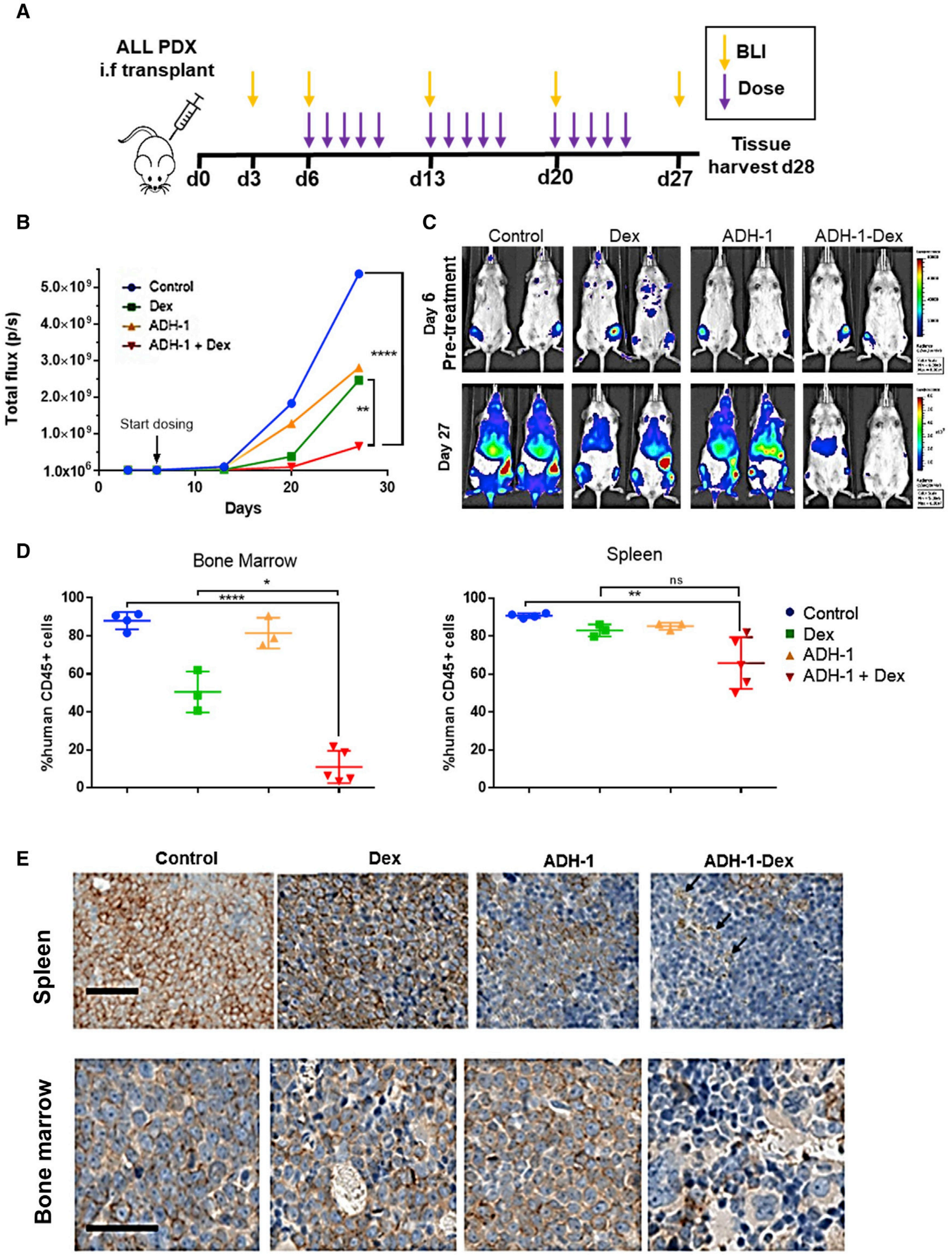


**Figure 7. ADH-1 potentiates dexamethasone sensitivity *in vivo***

(A) The PDX *in vivo* efficacy study design. Mice were dosed interperitoneally with either saline vehicle (control), 3 mg/kg dexamethasone (Dex), 200 mg/kg ADH-1, or ADH-1-Dex combined, 1× daily, 5× weekly for 3 weeks (15 doses), 5 mice per treatment group.

(B) Mean whole-body total flux measurements from bioluminescent imaging of each treatment group.

(C) Representative luminescence images of mice before and after treatment. Mice at each time point are shown with identical luminescence scale for comparison. Leukemic blasts are present in the femurs of all of the mice at the start of treatment. Signal spreads to BM sites, liver, and spleen in control mice, whereas signal is barely visible in ADH-1-Dex controls.

*(legend continued on next page)*

## AUTHOR CONTRIBUTIONS

Conceptualization, D.P. Methodology, D.P. and H.B. Investigation, D.P., H.B., J.P., S.H., M.B., M.S., R.T., R.N., H.M., S.H.A., A.W., S.L.N., S.N., P.Z., C.S., P.S., L.J.R., and J.C. Visualization, D.P. and H.B. Validation, D.P. and H.B. Resources, D.P., H.B., J.V., and O.H. Funding acquisition, D.P., J.V., and O.H. Project administration, D.P. and H.B. Supervision, D.P., H.B., J.V., and O.H. Writing – original draft, D.P. and H.B. Writing – review & editing, D.P., J.V., O.H., A.V.M., C.J.H., J.M.A., and C.H.

## DECLARATION OF INTERESTS

The authors declare no competing interests.

Received: November 23, 2021
Revised: May 18, 2022
Accepted: July 19, 2022
Published: August 16, 2022

## REFERENCES

1. Agarwal, P., Isringhausen, S., Li, H., Paterson, A.J., He, J., Gomariz, Á., Nagasawa, T., Nombela-Arrieta, C., and Bhatia, R. (2019). Mesenchymal niche-specific expression of Cxcl12 controls quiescence of treatment-resistant leukemia stem cells. Cell Stem Cell *24*, 769–784.e6. https://doi.org/10.1016/j.stem.2019.02.018.
2. Duan, C.W., Shi, J., Chen, J., Wang, B., Yu, Y.H., Qin, X., Zhou, X.C., Cai, Y.J., Li, Z.Q., Zhang, F., et al. (2014). Leukemia propagating cells rebuild an evolving niche in response to therapy. Cancer Cell *25*, 778–793. https://doi.org/10.1016/j.ccr.2014.04.015.
3. Guarnerio, J., Mendez, L.M., Asada, N., Menon, A.V., Fung, J., Berry, K., Frenette, P.S., Ito, K., and Pandolfi, P.P. (2018). A non-cell-autonomous role for Pml in the maintenance of leukemia from the niche. Nat. Commun. *9*, 66. https://doi.org/10.1038/s41467-017-02427-x.
4. Zhang, B., Nguyen, L.X.T., Li, L., Zhao, D., Kumar, B., Wu, H., Lin, A., Pellicano, F., Hopcroft, L., Su, Y.L., et al. (2018). Bone marrow niche trafficking of miR-126 controls the self-renewal of leukemia stem cells in chronic myelogenous leukemia. Nat. Med. *24*, 450–462. https://doi.org/10.1038/nm.4499.
5. Batlle, E., and Clevers, H. (2017). Cancer stem cells revisited. Nat. Med. *23*, 1124–1134. https://doi.org/10.1038/nm.4409.
6. Doupé, D.P., Klein, A.M., Simons, B.D., and Jones, P.H. (2010). The ordered architecture of murine ear epidermis is maintained by progenitor cells with random fate. Dev. Cell *18*, 317–323. https://doi.org/10.1016/j.devcel.2009.12.016.
7. Bonnet, D., and Dick, J.E. (1997). Human acute myeloid leukemia is organized as a hierarchy that originates from a primitive hematopoietic cell. Nat. Med. *3*, 730–737. https://doi.org/10.1038/nm0797-730.
8. Chiarini, F., Lonetti, A., Evangelisti, C., Buontempo, F., Orsini, E., Evangelisti, C., Cappellini, A., Neri, L.M., McCubrey, J.A., and Martelli, A.M. (2016). Advances in understanding the acute lymphoblastic leukemia bone marrow microenvironment: from biology to therapeutic targeting. Biochim. Biophys. Acta *1863*, 449–463. https://doi.org/10.1016/j.bbamcr.2015.08.015.
9. Dander, E., Palmi, C., D'Amico, G., and Cazzaniga, G. (2021). The bone marrow niche in B-cell acute lymphoblastic leukemia: the role of microenvironment from pre-leukemia to overt leukemia. Int. J. Mol. Sci. *22*, 4426. https://doi.org/10.3390/ijms22094426.
10. Delahaye, M.C., Salem, K.I., Pelletier, J., Aurrand-Lions, M., and Mancini, S.J.C. (2020). Toward therapeutic targeting of bone marrow leukemic niche protective signals in B-cell acute lymphoblastic leukemia. Front. Oncol. *10*, 606540. https://doi.org/10.3389/fonc.2020.606540.
11. Akinduro, O., Weber, T.S., Ang, H., Haltalli, M.L.R., Ruivo, N., Duarte, D., Rashidi, N.M., Hawkins, E.D., Duffy, K.R., and Lo Celso, C. (2018). Proliferation dynamics of acute myeloid leukaemia and haematopoietic progenitors competing for bone marrow space. Nat. Commun. *9*, 519. https://doi.org/10.1038/s41467-017-02376-5.
12. Duarte, D., Hawkins, E.D., Akinduro, O., Ang, H., De Filippo, K., Kong, I.Y., Haltalli, M., Ruivo, N., Straszkowski, L., Vervoort, S.J., et al. (2018). Inhibition of endosteal vascular niche remodeling rescues hematopoietic stem cell loss in AML. Cell Stem Cell *22*, 64–77.e6. https://doi.org/10.1016/j.stem.2017.11.006.
13. Churchman, M.L., and Mullighan, C.G. (2017). Ikaros: exploiting and targeting the hematopoietic stem cell niche in B-progenitor acute lymphoblastic leukemia. Exp. Hematol. *46*, 1–8. https://doi.org/10.1016/j.exphem.2016.11.002.
14. Joshi, I., Yoshida, T., Jena, N., Qi, X., Zhang, J., Van Etten, R.A., and Georgopoulos, K. (2014). Loss of Ikaros DNA-binding function confers integrin-dependent survival on pre-B cells and progression to acute lymphoblastic leukemia. Nat. Immunol. *15*, 294–304. https://doi.org/10.1038/ni.2821.
15. Pal, D., Blair, H.J., Elder, A., Dormon, K., Rennie, K.J., Coleman, D.J.L., Weiland, J., Rankin, K.S., Filby, A., Heidenreich, O., and Vormoor, J. (2016). Long-term in vitro maintenance of clonal abundance and leukaemia-initiating potential in acute lymphoblastic leukaemia. Leukemia *30*, 1691–1700. https://doi.org/10.1038/leu.2016.79.
16. Manabe, A., Coustan-Smith, E., Behm, F.G., Raimondi, S.C., and Campana, D. (1992). Bone marrow-derived stromal cells prevent apoptotic cell death in B-lineage acute lymphoblastic leukemia. Blood *79*, 2370–2377.
17. Mihara, K., Imai, C., Coustan-Smith, E., Dome, J.S., Dominici, M., Vanin, E., and Campana, D. (2003). Development and functional characterization of human bone marrow mesenchymal cells immortalized by enforced expression of telomerase. Br. J. Haematol. *120*, 846–849. https://doi.org/10.1046/j.1365-2141.2003.04217.x.
18. Alimperti, S., and Andreadis, S.T. (2015). CDH2 and CDH11 act as regulators of stem cell fate decisions. Stem Cell Res. *14*, 270–282. https://doi.org/10.1016/j.scr.2015.02.002.
19. Nelson, W.J., and Nusse, R. (2004). Convergence of Wnt, beta-catenin, and cadherin pathways. Science *303*, 1483–1487. https://doi.org/10.1126/science.1094291.
20. Zhang, B., Li, M., McDonald, T., Holyoake, T.L., Moon, R.T., Campana, D., Shultz, L., and Bhatia, R. (2013). Microenvironmental protection of CML stem and progenitor cells from tyrosine kinase inhibitors through N-cadherin and Wnt-beta-catenin signaling. Blood *121*, 1824–1838. https://doi.org/10.1182/blood-2012-02-412890.
21. Liang, S.M., Lu, Y.J., Ko, B.S., Jan, Y.J., Shyue, S.K., Yet, S.F., and Liou, J.Y. (2017). Cordycepin disrupts leukemia association with mesenchymal stromal cells and eliminates leukemia stem cell activity. Sci. Rep. *7*, 43930. https://doi.org/10.1038/srep43930.
22. Nygren, M.K., Døsen-Dahl, G., Stubberud, H., Wälchli, S., Munthe, E., and Rian, E. (2009). beta-catenin is involved in N-cadherin-dependent adhesion, but not in canonical Wnt signaling in E2A-PBX1-positive B acute lymphoblastic leukemia cells. Exp. Hematol. *37*, 225–233. https://doi.org/10.1016/j.exphem.2008.10.007.

(D) Leukemic engraftment in harvested BM and spleen measured by flow cytometry of labeled harvested cells. Human $CD45^+$ cells are shown as a percentage of total $CD45^+$ cells (mouse + human cells). Lines indicate means and SEs, symbols for individual mice. ANOVA (GraphPad Prism), ns not significant, $*p < 0.05$, $**p < 0.005$, $****p < 0.00005$.
(E) Human CD19 immunohistochemistry on sections of spleen and bone harvested from mice. Mice treated with ADH-1-Dex combination have few CD19-stained cells (brown staining at the cell membranes) and have areas of punctate staining indicative of cell debris (arrows). Scale bar, 50 $\mu$m.

23. Beasley, G.M., McMahon, N., Sanders, G., Augustine, C.K., Selim, M.A., Peterson, B., Norris, R., Peters, W.P., Ross, M.I., and Tyler, D.S. (2009). A phase 1 study of systemic ADH-1 in combination with melphalan via isolated limb infusion in patients with locally advanced in-transit malignant melanoma. Cancer *115*, 4766–4774. https://doi.org/10.1002/cncr.24509.

24. Yarom, N., Stewart, D., Malik, R., Wells, J., Avruch, L., and Jonker, D.J. (2013). Phase I clinical trial of Exherin (ADH-1) in patients with advanced solid tumors. Curr. Clin. Pharmacol. *8*, 81–88.

25. Beasley, G.M., Riboh, J.C., Augustine, C.K., Zager, J.S., Hochwald, S.N., Grobmyer, S.R., Peterson, B., Royal, R., Ross, M.I., and Tyler, D.S. (2011). Prospective multicenter phase II trial of systemic ADH-1 in combination with melphalan via isolated limb infusion in patients with advanced extremity melanoma. J. Clin. Oncol. *29*, 1210–1215. https://doi.org/10.1200/JCO.2010.32.1224.

26. Yoshioka, N., Gros, E., Li, H.R., Kumar, S., Deacon, D.C., Maron, C., Muotri, A.R., Chi, N.C., Fu, X.D., Yu, B.D., and Dowdy, S.F. (2013). Efficient generation of human iPSCs by a synthetic self-replicative RNA. Cell Stem Cell *13*, 246–254. https://doi.org/10.1016/j.stem.2013.06.001.

27. Warren, L., Manos, P.D., Ahfeldt, T., Loh, Y.H., Li, H., Lau, F., Ebina, W., Mandal, P.K., Smith, Z.D., Meissner, A., et al. (2010). Highly efficient reprogramming to pluripotency and directed differentiation of human cells with synthetic modified mRNA. Cell Stem Cell *7*, 618–630. https://doi.org/10.1016/j.stem.2010.08.012.

28. Evseenko, D., Zhu, Y., Schenke-Layland, K., Kuo, J., Latour, B., Ge, S., Scholes, J., Dravid, G., Li, X., MacLellan, W.R., and Crooks, G.M. (2010). Mapping the first stages of mesoderm commitment during differentiation of human embryonic stem cells. Proc. Natl. Acad. Sci. USA *107*, 13742–13747. https://doi.org/10.1073/pnas.1002077107.

29. Galipeau, J., and Sensébé, L. (2018). Mesenchymal stromal cells: clinical challenges and therapeutic opportunities. Cell Stem Cell *22*, 824–833. https://doi.org/10.1016/j.stem.2018.05.004.

30. Brachvogel, B., Moch, H., Pausch, F., Schlötzer-Schrehardt, U., Hofmann, C., Hallmann, R., von der Mark, K., Winkler, T., and Pöschl, E. (2005). Perivascular cells expressing annexin A5 define a novel mesenchymal stem cell-like population with the capacity to differentiate into multiple mesenchymal lineages. Development *132*, 2657–2668. https://doi.org/10.1242/dev.01846.

31. Turlo, K.A., Scapa, J., Bagher, P., Jones, A.W., Feil, R., Korthuis, R.J., Segal, S.S., and Iruela-Arispe, M.L. (2013). beta1-integrin is essential for vasoregulation and smooth muscle survival in vivo. Arterioscler. Thromb. Vasc. Biol. *33*, 2325–2335. https://doi.org/10.1161/ATVBAHA.112.300648.

32. Qi, D., Wei, M., Jiao, S., Song, Y., Wang, X., Xie, G., Taranto, J., Liu, Y., Duan, Y., Yu, B., et al. (2019). Hypoxia inducible factor 1alpha in vascular smooth muscle cells promotes angiotensin II-induced vascular remodeling via activation of CCL7-mediated macrophage recruitment. Cell Death Dis. *10*, 544. https://doi.org/10.1038/s41419-019-1757-0.

33. Yue, L., Bian, J.T., Grizelj, I., Cavka, A., Phillips, S.A., Makino, A., and Mazzone, T. (2012). Apolipoprotein E enhances endothelial-NO production by modulating caveolin 1 interaction with endothelial NO synthase. Hypertension *60*, 1040–1046. https://doi.org/10.1161/HYPERTENSIONAHA.112.196667.

34. Osborn, L., Hession, C., Tizard, R., Vassallo, C., Luhowskyj, S., Chi-Rosso, G., and Lobb, R. (1989). Direct expression cloning of vascular cell adhesion molecule 1, a cytokine-induced endothelial protein that binds to lymphocytes. Cell *59*, 1203–1211. https://doi.org/10.1016/0092-8674(89)90775-7.

35. Rice, G.E., and Bevilacqua, M.P. (1989). An inducible endothelial cell surface glycoprotein mediates melanoma adhesion. Science *246*, 1303–1306. https://doi.org/10.1126/science.2588007.

36. Imaizumi, T., Yoshida, H., and Satoh, K. (2004). Regulation of CX3CL1/fractalkine expression in endothelial cells. J. Atheroscler. Thromb. *11*, 15–21. https://doi.org/10.5551/jat.11.15.

37. Szukiewicz, D., Kochanowski, J., Pyzlak, M., Szewczyk, G., Stangret, A., and Mittal, T.K. (2013). Fractalkine (CX3CL1) and its receptor CX3CR1 may contribute to increased angiogenesis in diabetic placenta. Mediat. Inflamm. *2013*, 437576. https://doi.org/10.1155/2013/437576.

38. Kanayasu-Toyoda, T., Ishii-Watabe, A., Kikuchi, Y., Kitagawa, H., Suzuki, H., Tamura, H., Tada, M., Suzuki, T., Mizuguchi, H., and Yamaguchi, T. (2018). Occludin as a functional marker of vascular endothelial cells on tube-forming activity. J. Cell. Physiol. *233*, 1700–1711. https://doi.org/10.1002/jcp.26082.

39. Raghunandan, S., Ramachandran, S., Ke, E., Miao, Y., Lal, R., Chen, Z.B., and Subramaniam, S. (2021). Heme oxygenase-1 at the nexus of endothelial cell fate decision under oxidative stress. Front. Cell Dev. Biol. *9*, 702974. https://doi.org/10.3389/fcell.2021.702974.

40. Belkhiri, A., Richards, C., Whaley, M., McQueen, S.A., and Orr, F.W. (1997). Increased expression of activated matrix metalloproteinase-2 by human endothelial cells after sublethal H2O2 exposure. Lab. Invest. *77*, 533–539.

41. Davenport, A.P., Hyndman, K.A., Dhaun, N., Southan, C., Kohan, D.E., Pollock, J.S., Pollock, D.M., Webb, D.J., and Maguire, J.J. (2016). Endothelin. Pharmacol. Rev. *68*, 357–418. https://doi.org/10.1124/pr.115.011833.

42. Goncharov, N.V., Nadeev, A.D., Jenkins, R.O., and Avdonin, P.V. (2017). Markers and biomarkers of endothelium: when something is rotten in the state. Oxid. Med. Cell. Longev. *2017*, 9759735. https://doi.org/10.1155/2017/9759735.

43. Suri, C., Jones, P.F., Patan, S., Bartunkova, S., Maisonpierre, P.C., Davis, S., Sato, T.N., and Yancopoulos, G.D. (1996). Requisite role of angiopoietin-1, a ligand for the TIE2 receptor, during embryonic angiogenesis. Cell *87*, 1171–1180. https://doi.org/10.1016/s0092-8674(00)81813-9.

44. Rossi, E., Bernabeu, C., and Smadja, D.M. (2019). Endoglin as an adhesion molecule in mature and progenitor endothelial cells: a function beyond TGF-beta. Front. Med. *6*, 10. https://doi.org/10.3389/fmed.2019.00010.

45. Dmitrieva, N.I., and Burg, M.B. (2014). Secretion of von Willebrand factor by endothelial cells links sodium to hypercoagulability and thrombosis. Proc. Natl. Acad. Sci. USA *111*, 6485–6490. https://doi.org/10.1073/pnas.1404809111.

46. Aghajanian, H., Cho, Y.K., Rizer, N.W., Wang, Q., Li, L., Degenhardt, K., and Jain, R. (2017). Pdgfralpha functions in endothelial-derived cells to regulate neural crest cells and the development of the great arteries. Dis. Model. Mech. *10*, 1101–1108. https://doi.org/10.1242/dmm.029710.

47. Göoz, P., Göoz, M., Baldys, A., and Hoffman, S. (2009). ADAM-17 regulates endothelial cell morphology, proliferation, and in vitro angiogenesis. Biochem. Biophys. Res. Commun. *380*, 33–38. https://doi.org/10.1016/j.bbrc.2009.01.013.

48. Bender, H.R., Campbell, G.E., Aytoda, P., Mathiesen, A.H., and Duffy, D.M. (2019). Thrombospondin 1 (THBS1) promotes follicular angiogenesis, luteinization, and ovulation in primates. Front. Endocrinol. *10*, 727. https://doi.org/10.3389/fendo.2019.00727.

49. Bender, H.R., Trau, H.A., and Duffy, D.M. (2018). Placental growth factor is required for ovulation, luteinization, and angiogenesis in primate ovulatory follicles. Endocrinology *159*, 710–722. https://doi.org/10.1210/en.2017-00739.

50. Deng, C., Zhang, D., Shan, S., Wu, J., Yang, H., and Yu, Y. (2007). Angiogenic effect of intercellular adhesion molecule-1. J Huazhong Univ Sci Technolog Med Sci *27*, 9–12. https://doi.org/10.1007/s11596-007-0103-4.

51. Haferlach, T., Kohlmann, A., Wieczorek, L., Basso, G., Kronnie, G.T., Béné, M.C., De Vos, J., Hernández, J.M., Hofmann, W.K., Mills, K.I., et al. (2010). Clinical utility of microarray-based gene expression profiling in the diagnosis and subclassification of leukemia: report from the International Microarray Innovations in Leukemia Study Group. J. Clin. Oncol. *28*, 2529–2537. https://doi.org/10.1200/JCO.2009.23.4732.

52. Bagger, F.O., Kinalis, S., and Rapin, N. (2019). BloodSpot: a database of healthy and malignant haematopoiesis updated with purified and single cell mRNA sequencing profiles. Nucleic Acids Res. *47*, D881–D885. https://doi.org/10.1093/nar/gky1076.

53. Ebinger, S., Özdemir, E.Z., Ziegenhain, C., Tiedt, S., Castro Alves, C., Grunert, M., Dworzak, M., Lutz, C., Turati, V.A., Enver, T., et al. (2016). Characterization of rare, dormant, and therapy-resistant cells in acute lymphoblastic leukemia. Cancer Cell *30*, 849–862. https://doi.org/10.1016/j.ccell.2016.11.002.

54. Fath, S., Bauer, A.P., Liss, M., Spriestersbach, A., Maertens, B., Hahn, P., Ludwig, C., Schäfer, F., Graf, M., and Wagner, R. (2011). Multiparameter RNA and codon optimization: a standardized tool to assess and enhance autologous mammalian gene expression. PLoS One *6*, e17596. https://doi.org/10.1371/journal.pone.0017596.

55. Perotti, A., Sessa, C., Mancuso, A., Noberasco, C., Cresta, S., Locatelli, A., Carcangiu, M.L., Passera, K., Braghetti, A., Scaramuzza, D., et al. (2009). Clinical and pharmacological phase I evaluation of Exherin (ADH-1), a selective anti-N-cadherin peptide in patients with N-cadherin-expressing solid tumours. Ann. Oncol. *20*, 741–745. https://doi.org/10.1093/annonc/mdn695.

56. Shintani, Y., Fukumoto, Y., Chaika, N., Grandgenett, P.M., Hollingsworth, M.A., Wheelock, M.J., and Johnson, K.R. (2008). ADH-1 suppresses N-cadherin-dependent pancreatic cancer progression. Int. J. Cancer *122*, 71–77. https://doi.org/10.1002/ijc.23027.

57. Nagel, R., Semenova, E.A., and Berns, A. (2016). Drugging the addict: non-oncogene addiction as a target for cancer therapy. EMBO Rep. *17*, 1516–1531. https://doi.org/10.15252/embr.201643030.

58. Workman, P., Al-Lazikani, B., and Clarke, P.A. (2013). Genome-based cancer therapeutics: targets, kinase drug resistance and future strategies for precision oncology. Curr. Opin. Pharmacol. *13*, 486–496. https://doi.org/10.1016/j.coph.2013.06.004.

59. Bomken, S., Buechler, L., Rehe, K., Ponthan, F., Elder, A., Blair, H., Bacon, C.M., Vormoor, J., and Heidenreich, O. (2013). Lentiviral marking of patient-derived acute lymphoblastic leukaemic cells allows in vivo tracking of disease progression. Leukemia *27*, 718–721. https://doi.org/10.1038/leu.2012.206.

60. Elder, A., Bomken, S., Wilson, I., Blair, H.J., Cockell, S., Ponthan, F., Dormon, K., Pal, D., Heidenreich, O., and Vormoor, J. (2017). Abundant and equipotent founder cells establish and maintain acute lymphoblastic leukaemia. Leukemia *31*, 2577–2586. https://doi.org/10.1038/leu.2017.140.

61. le Viseur, C., Hotfilder, M., Bomken, S., Wilson, K., Röttgers, S., Schrauder, A., Rosemann, A., Irving, J., Stam, R.W., Shultz, L.D., et al. (2008). In childhood acute lymphoblastic leukemia, blasts at different stages of immunophenotypic maturation have stem cell properties. Cancer Cell *14*, 47–58. https://doi.org/10.1016/j.ccr.2008.05.015.

62. Turley, R.S., Tokuhisa, Y., Toshimitsu, H., Lidsky, M.E., Padussis, J.C., Fontanella, A., Deng, W., Augustine, C.K., Beasley, G.M., Davies, M.A., et al. (2015). Targeting N-cadherin increases vascular permeability and differentially activates AKT in melanoma. Ann. Surg. *261*, 368–377. https://doi.org/10.1097/SLA.0000000000000635.

63. Matheson, E.C., Thomas, H., Case, M., Blair, H., Jackson, R.K., Masic, D., Veal, G., Halsey, C., Newell, D.R., Vormoor, J., and Irving, J.A.E. (2019). Glucocorticoids and selumetinib are highly synergistic in RAS pathway-mutated childhood acute lymphoblastic leukemia through upregulation of BIM. Haematologica *104*, 1804–1811. https://doi.org/10.3324/haematol.2017.185975.

64. Jackson, R.K., Liebich, M., Berry, P., Errington, J., Liu, J., Parker, C., Moppett, J., Samarasinghe, S., Hough, R., Rowntree, C., et al. (2019). Impact of dose and duration of therapy on dexamethasone pharmacokinetics in childhood acute lymphoblastic leukaemia-a report from the UKALL 2011 trial. Eur. J. Cancer *120*, 75–85. https://doi.org/10.1016/j.ejca.2019.07.026.

65. Pal, D., Heidenreich, O., and Vormoor, J. (2016). Dormancy stems the tide of chemotherapy. Cancer Cell *30*, 825–826. https://doi.org/10.1016/j.ccell.2016.11.014.

66. Kumar, B., Garcia, M., Weng, L., Jung, X., Murakami, J.L., Hu, X., McDonald, T., Lin, A., Kumar, A.R., DiGiusto, D.L., et al. (2018). Acute myeloid leukemia transforms the bone marrow niche into a leukemia-permissive microenvironment through exosome secretion. Leukemia *32*, 575–587. https://doi.org/10.1038/leu.2017.259.

67. Baryawno, N., Przybylski, D., Kowalczyk, M.S., Kfoury, Y., Severe, N., Gustafsson, K., Kokkaliaris, K.D., Mercier, F., Tabaka, M., Hofree, M., et al. (2019). A cellular taxonomy of the bone marrow stroma in homeostasis and leukemia. Cell *177*, 1915–1932.e16. https://doi.org/10.1016/j.cell.2019.04.040.

68. Boyd, A.L., Aslostovar, L., Reid, J., Ye, W., Tanasijevic, B., Porras, D.P., Shapovalova, Z., Almakadi, M., Foley, R., Leber, B., et al. (2018). Identification of chemotherapy-induced leukemic-regenerating cells reveals a transient vulnerability of human AML recurrence. Cancer Cell *34*, 483–498.e5. https://doi.org/10.1016/j.ccell.2018.08.007.

69. Martinez-Soria, N., McKenzie, L., Draper, J., Ptasinska, A., Issa, H., Potluri, S., Blair, H.J., Pickin, A., Isa, A., Chin, P.S., et al. (2018). The oncogenic transcription factor RUNX1/ETO corrupts cell cycle regulation to drive leukemic transformation. Cancer Cell *34*, 626–642.e8. https://doi.org/10.1016/j.ccell.2018.08.015.

70. Wheelock, M.J., and Johnson, K.R. (2003). Cadherin-mediated cellular signaling. Curr. Opin. Cell Biol. *15*, 509–514. https://doi.org/10.1016/s0955-0674(03)00101-7.

71. Mangolini, M., Götte, F., Moore, A., Ammon, T., Oelsner, M., Lutzny-Geier, G., Klein-Hitpass, L., Williamson, J.C., Lehner, P.J., Dürig, J., et al. (2018). Notch2 controls non-autonomous Wnt-signalling in chronic lymphocytic leukaemia. Nat. Commun. *9*, 3839. https://doi.org/10.1038/s41467-018-06069-5.

72. Mrozik, K.M., Blaschuk, O.W., Cheong, C.M., Zannettino, A.C.W., and Vandyke, K. (2018). N-cadherin in cancer metastasis, its emerging role in haematological malignancies and potential as a therapeutic target in cancer. BMC Cancer *18*, 939. https://doi.org/10.1186/s12885-018-4845-0.

73. Duy, C., Li, M., Teater, M., Meydan, C., Garrett-Bakelman, F.E., Lee, T.C., Chin, C.R., Durmaz, C., Kawabata, K.C., Dhimolea, E., et al. (2021). Chemotherapy induces senescence-like resilient cells capable of initiating AML recurrence. Cancer Discov. *11*, 1542–1561. https://doi.org/10.1158/2159-8290.CD-20-1375.

74. Banerjee, P., Kotla, S., Reddy Velatooru, L., Abe, R.J., Davis, E.A., Cooke, J.P., Schadler, K., Deswal, A., Herrmann, J., Lin, S.H., et al. (2021). Senescence-associated secretory phenotype as a hinge between cardiovascular diseases and cancer. Front. Cardiovasc. Med. *8*, 763930. https://doi.org/10.3389/fcvm.2021.763930.

75. Williams, E., Williams, G., Gour, B.J., Blaschuk, O.W., and Doherty, P. (2000). A novel family of cyclic peptide antagonists suggests that N-cadherin specificity is determined by amino acids that flank the HAV motif. J. Biol. Chem. *275*, 4007–4012. https://doi.org/10.1074/jbc.275.6.4007.

76. Wanner, I.B., and Wood, P.M. (2002). N-cadherin mediates axon-aligned process growth and cell-cell interaction in rat Schwann cells. J. Neurosci. 22, 4066–4079.

77. Augustine, C.K., Yoshimoto, Y., Gupta, M., Zipfel, P.A., Selim, M.A., Febbo, P., Pendergast, A.M., Peters, W.P., and Tyler, D.S. (2008). Targeting N-cadherin enhances antitumor activity of cytotoxic therapies in melanoma treatment. Cancer Res. *68*, 3777–3784. https://doi.org/10.1158/0008-5472.CAN-07-5949.

78. Ivaska, J., and Heino, J. (2010). Interplay between cell adhesion and growth factor receptors: from the plasma membrane to the endosomes. Cell Tissue Res. *339*, 111–120. https://doi.org/10.1007/s00441-009-0857-z.

79. Altan-Bonnet, G., and Mukherjee, R. (2019). Cytokine-mediated communication: a quantitative appraisal of immune complexity. Nat. Rev. Immunol. *19*, 205–217. https://doi.org/10.1038/s41577-019-0131-x.

80. Weiss, A.R.R., and Dahlke, M.H. (2019). Immunomodulation by mesenchymal stem cells (MSCs): mechanisms of action of living, apoptotic, and dead MSCs. Front. Immunol. *10*, 1191. https://doi.org/10.3389/fimmu.2019.01191.

81. Vodyanik, M.A., Yu, J., Zhang, X., Tian, S., Stewart, R., Thomson, J.A., and Slukvin, I.I. (2010). A mesoderm-derived precursor for mesenchymal stem and endothelial cells. Cell Stem Cell *7*, 718–729. https://doi.org/10.1016/j.stem.2010.11.011.

82. Sinclair, P.B., Blair, H.H., Ryan, S.L., Buechler, L., Cheng, J., Clayton, J., Hanna, R., Hollern, S., Hawking, Z., Bashton, M., et al. (2018). Dynamic clonal progression in xenografts of acute lymphoblastic leukemia with intrachromosomal amplification of chromosome 21. Haematologica *103*, 634–644. https://doi.org/10.3324/haematol.2017.172304.

83. Wingett, S.W., and Andrews, S. (2018). FastQ Screen: a tool for multi-genome mapping and quality control. F1000Res. *7*, 1338. https://doi.org/10.12688/f1000research.15931.2.

84. Patro, R., Duggal, G., Love, M.I., Irizarry, R.A., and Kingsford, C. (2017). Salmon provides fast and bias-aware quantification of transcript expression. Nat. Methods *14*, 417–419. https://doi.org/10.1038/nmeth.4197.

85. Soneson, C., Love, M.I., and Robinson, M.D. (2015). Differential analyses for RNA-seq: transcript-level estimates improve gene-level inferences. F1000Res. *4*, 1521. https://doi.org/10.12688/f1000research.7563.2.

86. Love, M.I., Huber, W., and Anders, S. (2014). Moderated estimation of fold change and dispersion for RNA-seq data with DESeq2. Genome Biol. *15*, 550. https://doi.org/10.1186/s13059-014-0550-8.

87. Li, H., and Durbin, R. (2009). Fast and accurate short read alignment with Burrows-Wheeler transform. Bioinformatics *25*, 1754–1760. https://doi.org/10.1093/bioinformatics/btp324.

88. DePristo, M.A., Banks, E., Poplin, R., Garimella, K.V., Maguire, J.R., Hartl, C., Philippakis, A.A., del Angel, G., Rivas, M.A., Hanna, M., et al. (2011). A framework for variation discovery and genotyping using next-generation DNA sequencing data. Nat. Genet. *43*, 491–498. https://doi.org/10.1038/ng.806.

89. McKenna, A., Hanna, M., Banks, E., Sivachenko, A., Cibulskis, K., Kernytsky, A., Garimella, K., Altshuler, D., Gabriel, S., Daly, M., and DePristo, M.A. (2010). The Genome Analysis Toolkit: a MapReduce framework for analyzing next-generation DNA sequencing data. Genome Res. *20*, 1297–1303. https://doi.org/10.1101/gr.107524.110.

90. Van der Auwera, G.A., Carneiro, M.O., Hartl, C., Poplin, R., Del Angel, G., Levy-Moonshine, A., Jordan, T., Shakir, K., Roazen, D., Thibault, J., et al. (2013). From FastQ data to high confidence variant calls: the Genome Analysis Toolkit best practices pipeline. Curr. Protoc. Bioinformatics *43*, 11.10.1–11.10.33. https://doi.org/10.1002/0471250953.bi1110s43.

91. Cibulskis, K., Lawrence, M.S., Carter, S.L., Sivachenko, A., Jaffe, D., Sougnez, C., Gabriel, S., Meyerson, M., Lander, E.S., and Getz, G. (2013). Sensitive detection of somatic point mutations in impure and heterogeneous cancer samples. Nat. Biotechnol. *31*, 213–219. https://doi.org/10.1038/nbt.2514.

92. McLaren, W., Gil, L., Hunt, S.E., Riat, H.S., Ritchie, G.R.S., Thormann, A., Flicek, P., and Cunningham, F. (2016). The Ensembl variant effect predictor. Genome Biol. *17*, 122. https://doi.org/10.1186/s13059-016-0974-4.

93. Krzywinski, M., Schein, J., Birol, I., Connors, J., Gascoyne, R., Horsman, D., Jones, S.J., and Marra, M.A. (2009). Circos: an information aesthetic for comparative genomics. Genome Res. *19*, 1639–1645. https://doi.org/10.1101/gr.092759.109.

94. Adams, F.F., Heckl, D., Hoffmann, T., Talbot, S.R., Kloos, A., Thol, F., Heuser, M., Zuber, J., Schambach, A., and Schwarzer, A. (2017). An optimized lentiviral vector system for conditional RNAi and efficient cloning of microRNA embedded short hairpin RNA libraries. Biomaterials *139*, 102–115. https://doi.org/10.1016/j.biomaterials.2017.05.032.

CellPress
OPEN ACCESS

## STAR★METHODS

### KEY RESOURCES TABLE

| REAGENT or RESOURCE | SOURCE | IDENTIFIER |
|---|---|---|
| **Antibodies** | | |
| StemCell Pluripotency Surface Marker Kit | CellSignaling Technology | 9656S |
| Anti-mouse IgG Alexaflour 488 secondary antibody | CellSignalling Technology | 4408S |
| Anti-mouse IgG Alexafluor 594 goat secondary antibody | Life Technologies, Paisley, UK | A-11032 |
| Normal goat serum | Dako, Cambridgeshire UK | X090710-8 |
| Anti-OCT4 Alexafluor 488 antibody | Merck Millipore, Hertfordshire, UK | MA1-104-D488 |
| Anti-SOX2 Alexafluor 647 antibody | Biolegend, London, UK | 656108 |
| ProLong gold antifade reagent with DAPI | Molecular Probes, Life Technologies, Paisley, UK | P36931 |
| **Chemicals, peptides, and recombinant proteins** | | |
| ADH-1, Exherin™ | AdooQ® Bioscience LLC, CA, US | Catalog No.: A13689 |
| Dexamethasone | Sigma-Aldrich, Dorset, UK | |
| **Biological samples** | | |
| E2A/HLF ; Sample at diagnosis | In house | L707D |
| E2A/HLF; Sample at relapse | In house | L707R |
| BCR/ABL | In house | L49120 |
| BCR/ABL | In house | L4951 |
| BCR/ABL | In house | L4967 |
| E2A/PBX1 | In house | UCL E2A/PBX1 |
| MLL/AF9 | In house | UCL MLL/AF9 |
| HYPODIPLOID | In house | UCL Hypodiploid |
| MLL/AF4 | In house | L826 |
| MLL/AF4 | In house | LK124 |
| iAMP21 | In house | G5004 |
| iAMP21 | In house | G3131 |
| iAMP21 | In house | G1062 |
| iAMP21 | In house | H7205 |
| iAMP21 | In house | G7578 |
| HIGH HYPERDIPLOID | In house | L914 |
| Karyotype unknown, ALL | In house | L897 |
| E2A/PBX1 | In house | L910 |
| BCR/ABL | In house | L590R |
| Hypodiploid adult | In house | OBL-15 |
| Karyotype unknown, T-ALL | In house | T-ALL 26 |
| Biphenotypic/MLL rearrangement | In house | MS40 |
| Human bone marrow mesenchymal stroma | In house | 6255 |
| Human bone marrow mesenchymal stroma | In house | 6257 |

(*Continued on next page*)

| REAGENT or RESOURCE | SOURCE | IDENTIFIER |
|---|---|---|
| *Continued* | | |
| Critical commercial assays | | |
| RNeasy mini kit | Qiagen, Manchester, UK | 74134 |
| RNase-free DNase kit | Qiagen, Manchester, UK | 79254 |
| RevertAid first strand cDNA synthesis kit | ThermoFisher Scientific, Hetfordshire, UK | K1631 |
| Alkaline Phosphatase Detection Kit | Merck Millipore, Hertfordshire, UK | SCR004 |
| $RT^2$ Profiler$^{TM}$ PCR Array Human Mesenchymal Stem Cells RT2 Profiler PCR Array | Qiagen, Manchester, UK | GeneGlobe ID - PAHS-082Z |
| $RT^2$ Profiler$^{TM}$ PCR Array Human Endothelial Cell Biology RT2 Profiler PCR Array | Qiagen, Manchester, UK | GeneGlobe ID - PAHS-015Z |
| $RT^2$ Profiler$^{TM}$ PCR Array Human Extracellular Matrix & Adhesion Molecules RT2 Profiler PCR Array | Qiagen, Manchester, UK | GeneGlobe ID - PAHS-013Z |
| $RT^2$ Profiler$^{TM}$ PCR Array Human Leukemia RT2 Profiler PCR Array | Qiagen, Manchester, UK | GeneGlobe ID - PAHS-137Z |
| $RT^2$ Profiler$^{TM}$ PCR Array Human Transcription Factors RT2 Profiler PCR Array | Qiagen, Manchester, UK | GeneGlobe ID - PAHS-075Z |
| Deposited data | | |
| Raw RNA sequencing data | This paper | GEO: GSE208060 |
| Whole exome sequencing raw data | This paper | Pal, Deepali (2022), "hiPSC -derived bone marrow milieu identifies a clinically actionable driver of niche-mediated treatment resistance in leukaemia 1", Mendeley Data, V1, https://doi.org/10.17632/dn3pvps68y.1 AND Pal, Deepali (2022), "hiPSC-derived bone marrow milieu identifies a clinically actionable driver of niche-mediated treatment resistance in leukaemia 2", Mendeley Data, V1, https://doi.org/10.17632/zmgy6sgxbh. |
| Experimental models: Cell lines | | |
| SEM | DSMZ | RRID:CVCL_0095, Cat# ACC 546 |
| HAL-01 | DSMZ | RRID:CVCL_1242, Cat# ACC 610 |
| PreB 697 | DSMZ | RRID:CVCL_0079, Cat # ACC 42 |
| Nalm6 | DSMZ | RRID:CVCL_0092, Cat # ACC 128 |
| SKNO | DSMZ | RRID: CVCL_2196, Cat # ACC 690 |
| Kasumi-1 | DSMZ | RRID: CVCL_0589, Cat # ACC 220 |
| REH | DSMZ | RRID:CVCL_1650, Cat # ACC 22 |
| HEK293T | DSMZ | RRID: CVCL_0063, Cat# ACC 305 |
| Oligonucleotides | | |
| CDH2 forward primer | Sigma-Aldrich, Dorset, UK | GGTGGAGGAGAAGAAGACCAG |
| CDH2 reverse primer | Sigma-Aldrich, Dorset, UK | GGCATCAGGCTCCACAGT |
| POU5F1 forward primer | Sigma-Aldrich, Dorset, UK | GCGATCAAGC AGCGACTA |
| POU5F1 reverse primer | Sigma-Aldrich, Dorset, UK | TTCACCTTCCC TCCAACC |
| NANOG forward primer | Sigma-Aldrich, Dorset, UK | CCAAATTCTC CTGCCAGTGA C |
| NANOG reverse primer | Sigma-Aldrich, Dorset, UK | CACGTGGTTT CCAAACAAGA AA |

(*Continued on next page*)

*Continued*

| REAGENT or RESOURCE | SOURCE | IDENTIFIER |
|---|---|---|
| GDF3 forward primer | Sigma-Aldrich, Dorset, UK | CTTATGCTAC GTAAAGGAGC TGGG |
| GDF3 reverse primer | Sigma-Aldrich, Dorset, UK | GTGCCAACCC AGGTCCCGGA AGTT |
| ZFP42 forward primer | Sigma-Aldrich, Dorset, UK | CGTACGCAAA TTAAAGTCCA GA |
| ZFP42 reverse primer | Sigma-Aldrich, Dorset, UK | CAGCATCCTA AACAGCTCGC AGAAT |
| DNMT3B forward primer | Sigma-Aldrich, Dorset, UK | TGCTGCTCAC AGGGCCCGAT ACTTC |
| DNMT3B reverse primer | Sigma-Aldrich, Dorset, UK | TCCTTTCGAG CTCAGTGCAC CACAAAAC |
| SNAI1 forward primer | Sigma-Aldrich, Dorset, UK | ACCACTATGC CGCGCTCTT |
| SNAI1 reverse primer | Sigma-Aldrich, Dorset, UK | GGTCGTAGGG CTGCTGGAA |
| SNAI2 forward primer | Sigma-Aldrich, Dorset, UK | TGTTGCAGTG AGGGCAAGAA |
| SNAI2 reverse primer | Sigma-Aldrich, Dorset, UK | GACCCTGGTT GCTTCAAGGA |
| CD90 forward primer | Sigma-Aldrich, Dorset, UK | CACACATACC GCTCCCGAAC C |
| CD90 reverse primer | Sigma-Aldrich, Dorset, UK | GCTGATGCCC TCACACTT |
| GAPDH forward primer | Sigma-Aldrich, Dorset, UK | GAAGGTGAAGGTCGGAGTC |
| GAPDH reverse primer | Sigma-Aldrich, Dorset, UK | GAAGATGGTGATGGGATTTC |
| Other | | |
| Low glucose DMEM | Sigma-Aldrich, Dorset, UK | D5546 |
| FGF-basic recombinant human protein (10µg) | Life Technologies, Paisley, UK | PHG0024 |
| Gentle Cell Dissociation Reagent | Stem Cell Technologies, Stem Cell, UK | N/A |
| Mesoderm Induction Media | Stem Cell Technologies, Stem Cell, UK | N/A |
| Vitronectin XF™ | Stem Cell Technologies, Stem Cell, UK | Catalog # 100-0763 |
| Cell Adhere Dilution Buffer | Stem Cell Technologies, Stem Cell, UK | N/A |
| MesenCult ACF Basal Medium | Stem Cell Technologies, Stem Cell, UK | N/A |
| MesenCult ACF 5X Supplement | Stem Cell Technologies, Stem Cell, UK | N/A |
| StemPro™ Adipogenesis Differentiation Kit | Thermo Fisher Scientific, Hertfordshire, UK | A1007001 |
| Matrigel hESC-Qualified Matrix | Thermo Fisher Scientific, Hertfordshire, UK | N/A |
| Low-glucose Dulbecco's Modified Eagle's Medium | Sigma-Aldrich, Dorset, UK | N/A |
| Oil Red O Dye | Sigma-Aldrich, Dorset, UK | N/A |
| StemPro™ Osteogenesis Differentiation Kit | Thermo Fisher Scientific, Hertfordshire, UK | A1007201 |
| Matrigel hESC-Qualified Matrix | Thermo Fisher Scientific, Hertfordshire, UK | N/A |
| Low-glucose Dulbecco's Modified Eagle's Medium | Sigma-Aldrich, Dorset, UK | D5546-500ML |
| Alizarin Red Dye | Sigma-Aldrich, Dorset, UK | N/A |
| StemPro™ Chondrogenesis Differentiation Kit | Thermo Fisher Scientific, Hertfordshire, UK | A1007101 |
| Safranin O Dye | Sigma-Aldrich, Dorset, UK | N/A |
| Mesoderm Induction Media | Stem Cell Technologies, Stem Cell, UK | N/A |
| Human Recombinant VEGF-165 | Stem Cell Technologies, Stem Cell, UK | N/A |

(*Continued on next page*)

*Continued*

| REAGENT or RESOURCE | SOURCE | IDENTIFIER |
|---|---|---|
| SB431542 | Stem Cell Technologies, Stem Cell, UK | N/A |
| Microvascular Endothelial Cell Growth Medium | Sigma-Aldrich, Dorset, UK | N/A |
| Lymphoprep$^{TM}$ | Stem Cell Technologies, Stem Cell, UK | N/A |
| Serum-Free Medium for Culture and Expansion of Hematopoietic Cells (SFEM II) | Stem Cell Technologies, Stem Cell, UK | Catalog # 09655 |
| CellTrace$^{TM}$ Violet Cell Proliferation Kit | Thermo Fisher Scientific, Hertfordshire, UK | N/A |
| Propidium Iodide (PI) | Stem Cell Technologies, Cambridge, UK | N/A |
| Hoechst33342 | Sigma-Aldrich, Dorset, UK | N/A |
| Pyronin Y | Sigma-Aldrich, Dorset, UK | N/A |
| APC Annexin V Apoptosis Detection Kit with PI | Biolegend | 640932 |

## RESOURCE AVAILABILITY

### Lead contact
Further information and requests for reagents and resources should be directed to and will be fulfilled by the lead contact, Deepali Pal (deepali.pal@northumbria.ac.uk).

### Materials availability
Requests for new materials generated in this paper are to be directed to and will be fulfilled (pending MTA and associated restrictions) by the lead contact.

### Data and code availability
- De-identified dataset generated here have been deposited at Mendeley Data and are publicly available as of the date of publication. Mendeley Data, https://doi.org/10.17632/dn3pvps68y.1 and Mendeley Data, https://doi.org/10.17632/zmgy6sgxbh.1 DOI is listed in the key resources table. RNA-seq raw dataset generated here has been deposited in the GEO repository and can be accessed using the Series Record accession: GSE208060.
- This paper does not report original code.
- Any additional information required to reanalyze the data reported in this paper is available from the lead contact upon request.

## EXPERIMENTAL MODEL AND SUBJECT DETAILS

### Ethical approval
Patient-derived leukemia blasts were obtained from the Newcastle Biobank (REC reference number 07/H0906/109 + 5). Samples obtained from UCL were made under Research Ethics Committee reference 14/EM/0134. All samples were obtained following written informed consent. All animal studies were carried out in accordance with UK Animals (Scientific Procedures) Act, 1986 under project licence P74687DB5 following approval of Newcastle University animal ethical review body (AWERB).

### *Cell lines*
Leukaemia cell lines used include: SEM (RRID:CVCL_0095, female), HAL-01 (RRID:CVCL_1242, female), PreB 697 (RRID:CVCL_0079, male), Nalm6 (RRID:CVCL_0092, male), SKNO-1 (RRID: CVCL_2196; male), Kasumi-1 (RRID: CVCL_0589; male) and REH (RRID:CVCL_1650, female). Cells were cultured in RPMI1640 media supplemented with 20% FBS, 4mM L-glutamine at 37°C in a humidified 5% $CO_2$ incubator. Cell lines were confirmed free from mycoplasma infection at regular intervals using a MycoAlert kit (Lonza, Slough, UK).

HEK293T cell line (RRID: CVCL_0063; female) was also used for purposes of lentivirus production. These were cultured in HEPES-modified DMEM medium supplemented with 10% FBS, 4mM L-glutamine and 1mM sodium pyruvate, incubated at 37°C in a humidified 5% $CO_2$ incubator.

Mesenchymal stroma cells isolated from non cancerous bone marrow of patients undergoing total hip replacements were isolated and cultured as described in a previous study.[15] These cells were cultured in Low Glucose DMEM media supplemented with 20% FBS, 1% Penicillin/Streptomycin and 4mM L-glutamine (GIBCO) at 37°C in a humidified 5% $CO_2$ incubator. Cell were subcultured in a 1:4 ratio once they reached 70% confluence.

***BM-iPSC reprogramming and culture***

iPSC reprogramming was performed on mesenchymal stroma cells isolated from bone marrow of hip replacement surgeries. Low passage stroma cells seeded at a density of 18,500 cells/cm2 were transfected using Simplicon$^{TM}$ RNA Reprogramming Kit, OKSG (Merck Millipore) following pre-treatment with B18R protein. Following puromycin selection over an 8 day period, cells were subjected to 10 μg/mL of bFGF (GIBCO), 1 μL/mL of Human iPS Reprogramming Boost Supplement II, 1000× (Merck Millipore) and mouse embryonic fibroblast conditioned media (R&D Systems). iPSC colonies appeared between Day 28–30 post transfection and were picked under a stem cell microdissection cabinet for subsequent cultures. iPSC cultures were maintained on Vitronectin XF$^{TM}$(Stemcell Technologies) coated plates in TeSR$^{TM}$2 media (Stemcell Technologies). BM-iPSC were subsequently differentiated to generate iMSC and iANG cells.

***BM-iPSC differentiation***

BM-iPSC lines were differentiated into mesenchymal stem cells, endothelial and perivascular cells through an intermediate early mesoderm route using protocols adapted from existing studies.[81] Briefly, mesoderm induction was carried in Mesoderm Induction Media (Stemcell Technologies) for 72 hours following which the cells were subjected to either mesenchymal or vascular specification steps. Mesenchymal differentiation was achieved by treating the early mesoderm cells with Low-glucose Dulbecco's Modified Eagle's Medium (SIGMA), 20% Heat Inactivated Foetal Bovine Serum (GIBCO) and 10 μg/mL of bFGF (GIBCO). Vascular specification was achieved by treating cells with Mesoderm Induction Media, 1μM Human Recombination VEGF-165 (Stemcell Technologies) and 1μM SB431542 (Stemcell Technologies) for 12 days following which $CD31^+$ cells were sorted using flow cytometry for the purposes of characterisation. All cells following vascular specification were maintained in Microvascular Endothelial Cell Growth Medium (Sigma-Aldrich) for subsequent co-cultures.

**In vivo *animal studies***

All mouse studies were carried out in accordance with UK Animals (Scientific Procedures) Act, 1986 under project license P74687DB5 following approval of Newcastle University animal ethical review body (AWERB). Mice were housed in specific pathogen free conditions in individually ventilated cages with sterile bedding, water and diet (Irradiated No. 3 breeding diet, SDS). Mice were checked daily to ensure good health. All procedures were performed in a laminar flow hood except bioluminescent imaging (BLI).

NSG mice (NOD.Cg-Prkdc$^{scid}$ Il2rg $^{tm1Wjl}$/SzJ) aged between 12 and 16 weeks, both male and female, from in-house colonies were used for transplantions. Mice were checked daily, weighed and examined at least once weekly during studies to ensure good health.

## METHOD DETAILS

### Study design

The aim of this study was to use leukaemia as a paradigm and detect a way to clinically target microenvironment mediated treatment resistance. In order to do this the study design had two goals: 1. To develop a tractable human cell based *ex vivo* BM milieu that would facilitate niche-mediated survival and proliferation of patient-derived cancer cells 2. To reveal microenvironment-dependent leukaemia biology including proliferation, dormancy and treatment resistance. We developed synthetic human BM niche cell types from BM mesenchymal stroma reprogrammed iPSC. *In vitro*, BM-iPSC were differentiated into mesenchymal stem cells, perivascular and endothelial – like cells. We conducted *in vitro* co-culture experiments that BM-iPSC-derived niche cells could support human BM-derived haematopoietic cells (3 donors) and patient-derived leukaemia samples (14 samples, 13 diagnostic and 1 relapse). We conducted gene expression profiling in niche primed blasts to assay niche mediated changes in adherens junction molecules (2 samples) and confirmed upregulation of CDH2 via qRT-PCR in 7 samples (6 diagnostic and 1 relapse). Consequent functional validation experiments included CDH2 knockdown in leukaemia cells (4 cell lines) and patient-derived leukaemia cell co-culture on iMSC$^{CDH2-}$ (3 diagnostic, 1 relapse sample). To validate if CDH2 could be therapeutically targeted in the clinics we performed *in vitro* drug dose response assays with CDH2-antagonist ADH-1 (single drug assay: 15 diagnostic, 1 relapse samples, combination: 3 samples). Unless stated all experiments were conducted with a minimum of two independent experimental repeats. All graphical plots show standard deviation as error bars. All other imaging or flow cytometry data show a representative example of the total number of experiments.

***Immunofluorescent staining***

Immunofluorescent staining was conducted on Vitronectin$^{TM}$ coated EZ chamber slides. BM-iPSC colonies were fixed using 4% formaldehyde solution for 20 minutes at room temperature. The cells were then washed twice with 1× PBS for 10 minutes. For nuclear staining, cells were permeabilised using 0.1% triton X- 100/1xPBS for 10 minutes at room temperature, then washed twice with 1× PBS for 10 minutes. Blocking solution (4% normal goat serum) was added for 30 minutes at room temperature. The cells were then incubated with the primary antibody (Table S4) using a 1:250 dilution overnight and then washed three times with 1× PBS for 10 minutes. Subsequently cells were incubated with the secondary antibody in 1:500 dilution for 60 minutes at room temperature before being washed three times with 1xPBS for 10 minutes. Nuclear counterstain DAPI was added to each well in 1:500 dilution

and incubated at room temperature for 10 minutes. Finally, the coverslip was mounted onto a slide using gold antifade reagent and slides were visualized using the Nikon A1 confocal fluorescent microscope.

### Alkaline phosphatase detection

BM-iPSC were cultured for a minimum of 5 days, when alkaline phosphatase (AP) signal is optimal. On day 6, the cells were washed three times in PBS for 10 minutes and fixed using 4% paraformaldehyde for 2 minutes. The cells were washed with 1X rinse buffer (TBST- 20mM Tris-HCL, pH 7.4, 0.15 NaCl, 0.05% Tween-20). Alkaline phosphatase staining solution was prepared fresh by mixing Fast Red Violet (FRV) with Naphthol AS-BI phosphate solution and sterilised distilled water in a 2:1:1 ratio and added to cover the base of the well for a 15 minute incubation in the dark. Subsequently cells were washed with 1× PBS and stored in PBS until analysis. Positively stained iPSC colonies could be seen by eye, a microscope was used to visualise the colonies in greater detail.

### Ex vivo co-cultures of patient derived leukaemia cells

Patient derived leukaemia samples and non malignant $CD45^+$ hematopoietic cells derived from human bone marrow were seeded on iMSC or iANG cultures at a seeding density of 0.5-1 million cells/mL in SFEMII media (Stemcell Technologies) using protocols adapted from existing studies.[15,69] I-niche cells were seeded onto Vitronectin XF™(Stemcell Technologies) coated plates. 24 hours later, the i-niche cells were seeded onto the coated plastic in their respective media at a seeding density of 10,000 cells/$cm^2$. Following another 24 hours leukaemia cells were seeded onto the i-niche cells with drug treatment starting on the following day and lasting for 7 days. Leukaemia cells were harvested from the co-cultures at end of experiment by trypsinization. Following this leukaemia cells were separated from the feeder cells using a 10μM cell strainer. Consequently tagged leukaemia cells (eg: with CTV) were further purified using fluorescence-activated cell sorting (FACSAria, BD Biosciences).

### Drug dose response

Single agent and combinatorial drug dose response assays were set up as previously described.[15,69] Briefly, patient-derived leukaemia cells were seeded at 0.5-1 million/mL density onto iMSC or iANG cells. Clinically relevant concentration of different treatment compounds were added 24 hours later and cells were harvested for manual counting after a 5 day period.

### Cell generational tracing

10mM CellTrace™ Violet (Life Technologies), Excitation/Emission: 405nm/450nm was used to stain patient derived leukaemia cells at a cell density of 1 million/mL in 1X phosphate buffered saline for a total of 20 minutes at 37C, 5% CO2 following which excess stain was removed and cells were immediately put into co-culture in SFEMII media for subsequent cell fate tracking and/or sorting using flow cytometry.

### Cell cycle and G0 analysis

Following co-culture cells were harvested as per existing protocols[15] and subsequently stained with 10 μg/mL of Hoechst 33342 (Sigma-Aldrich), Excitation/Emission: 350nm/450nm for 45 minutes at 37C, 5% CO2 at a cell density of 1 million/mL in SFEMII media. Following this, 5μL of 100 μg/mL Pyronin Y (Sigma-aldrich), Excitation/Emission: 480nm/575nm was added to each 1 million/mL sample and stained for a further 15 minutes in the same conditions. Samples were then transferred onto ice and analysed by flow cytometry.

### FISH

5 million cells were pelleted through centrifugation for 3 minutes at 1,200 rpm, supernatant was subsequently discarded and 10mL 0.075M potassium chloride (pre-heated to 37 degrees) was added dropwise whilst mixing on a vortex. Samples were Incubated for 10 min at 37°C and further centrifuged at 1,200 rpm for 5 minutes. Supernatant was discarded and pellet vortexed. Following this 1mL of fresh fixative (3:1 methanol: acetic acid) was added dropwise with continuous vortexing which was then topped up to 5mL. Following another centrifugation step 1mL of fresh fixative was added for subsequent hybridisation procedure.

Briefly, 0.2ul of FISH probes (Dakocytomation TCF3 FISH DNA probe split signal, Agilent for E2A/HLF samples OR RP11-773I18 fluorescently labelled BAC probes[82] to detect RUNX1 amplification in iAMP21 samples) were mixed with 2.8ul hybridisation buffer (Cytocell, New York, USA) and denatured at 75°C for five minutes followed by hybridisation at 37°C overnight. Coverslips were removed in 2× SSC and slides washed in 0.02% SSC with 0.003% NP-40 at 72 °C for two minutes followed incubation in 0.1% SSC at room temperature for two minutes. Slides were mounted with 10 ul DAPI (Vector laboratories, California, USA). Scoring was performed on an automated Olympus BX-61 florescence microscope with a ×100 oil objective using CytoVision 7.2 software (Leica Microsystems, Newcastle-upon-Tyne, UK). Where possible, more than 100 nuclei were scored for each FISH test by two independent analysts. A cut-off threshold of >5% was established by counting the number of abnormal (false positive) signals generated when probes were hybridised to normal cells.

### mRNA-sequencing and analysis

Sequencing libraries were prepared using the TruSeq Stranded mRNA Sample Preparation Kit [Illumina] following manufacturer's instructions. Pooled libraries were sequenced at 40 Million (2 x 75 bp) reads per sample using a NextSeq 500 and High Output Kit (150 cycles) [Illumina]. The quality of sequenced reads was assessed using FastQC,[83] which suggests high quality data with all reads have Phread score >30 across all bases. For each sample, transcript abundance was quantified from raw reads with Salmon (version 0.8.2)[84] using the reference human transcriptome (hg38) defined by GENCODE release 27. An R package Tximport (version 1.6.0)[85] was used to estimate gene-level abundance from Salmon's transcript-level counts. DESeq2 (version 1.18.1)[86] was used to generate gene-level normalized counts and to perform differential expression analysis.

### *Whole-exome sequencing data analysis*

Sequencing libraries were prepared using the Nextera Rapid Capture Exome Kit [Illumina] following manufacturer's instructions. Pooled libraries were sequenced at >90X coverage (2 x 75 bp) per sample using a NextSeq 500 and High Output Kit (150 cycles) [Illumina].Raw reads were aligned to human reference genome (hg19) using Burrows-Wheeler Aligner (BWA) 0.7.12[87] and were processed using the Genome Analysis Toolkit (GATK, v3.8) best practices recommended workflow for variant discovery analysis.[88–90] MuTect (v1.1.7) and MuTect2[91] were used to identify somatic variants (SNPs and INDELs) in the iMSC and iANG primed patient derived blasts that were not present in the blasts prior to co-culture. Variants were annotated using Ensembl Variant Effect Predictor (VEP, version 90).[92] Circos plots of exonic mutations with allele frequency >25% were generated using Circos.[93]

### *Quantitative RT-PCR*

RNA was extracted using Qiagen RNEasy Micro Procedure as per manufacturer's protocol following an on column DNAse removal step. RevertAidTMH Minus First Strand cDNA Synthesis Kit (ThermoFisher Scientific) was used to synthesise cDNA. 500ng RNA was collected and added to RNase/DEPC free water to a final volume of 11μL. 1μL (dN)6 (200 mg/L) random hexamers was added, mixed gently by inverting the vial and briefly centrifuged. Using a GeneAmp PCR system 2700 the sample was incubated at 65 °C for 5 minutes, after which the sample was immediately placed on ice, 8μL of the master mix (5X Reaction Buffer, 20U/μl RNase Inhibitor, 10mM dNTP and 100 U/μl RevertAid H Minus MMLV RT) was added, the samples were vortexed and briefly centrifuged. The samples were placed back in the PCR machine to incubate at 25°C for 10 minutes, 42°C for 60 minutes and 75°C for 10 minutes to terminate the reaction.

Primers were reconstituted in RNase/DNase free water to a working solution of 10μM. The PCR master mix (reverse and forward primer, Applied Biosystems SYBR-Green PCR master mix and RNase free water) was mixed well by gently pipetting the solution, 8μL/well was added to a 384-well PCR plate, 2μL cDNA was then added to each well to a total of 10μL/well. The plate was sealed and centrifuged for 1 minute at 1000RPM and placed in an Applied Biosystems 7900HT Sequence Detection System for 40 cycles. This included a denaturation step at 95°C, an annealing step at 60°C and an elongation step at 90°C.

For RT-PCR arrays (see Key Resources Table) cDNA was synthesised using RT2 First Strand Kit (Qiagen) and subsequent PCR step were performed as above but using RT2 SYBR Green ROX qPCR Mastermix (Qiagen) and PAHS-086ZE-4 - RT$^2$ Profiler$^{TM}$ arrays (Qiagen) as per manufacturers protocol. Data was normalized with respect to an average of 5 House Keeping Genes (HKG): ACTB, B2M, GAPDH, HPRT1, RPLP0.

### *Functional* in vitro *knockdown, leukaemia cell lines*

In order to engineer a doxycycline conditional RNAi approach oligos (shRNA guide strand) designed against CDH2 were cloned into a pL40C.T3.dTomato.miRN.PGK.Venus.IRES.rtTA-V10.WPRE backbone as per published protocols.[59,94] In this approach a human miR-30 backbone retaining native flanking sequences is combined with a lentiviral vector system that allows for conditional RNAi downregulation of genes. This is characterised by improved doxycycline sensitivity and mitigated leakiness. Oligonucleotide design was performed using an online tool that included Sensor criteria along with additional ranking criteria as per published protocols.[94] Following bacterial transformation and plasmid amplification DNA sequence was confirmed through sanger sequencing. Plasmid DNA was obtained using Endofree® Plasmid Kit (Qiagen) and introduced into HEK293T cells for lentivirus production. The HEK293T cells were grown in 150 mm tissue culture dishes at a concentration of 3 x 106 cells in 30 mL DMEM media the day prior to the co-transfection. On the following day, 45 μg packaging plasmid pCMVΔR8.91, 15 μg envelope plasmid pMD2.G, and 60 μg shRNA expression vector were co-transfected using calcium phosphate precipitation method. The cells were incubated for 72 hours and the recombinant pseudotyped lentivirus-containing supernatant was collected for subsequent concentration of the thus engineered lentivirus. Lentiviral transduction was performed on leukaemia cell lines as described previously and transduced (Venus + ve), doxycycline induced cells (dTomato + ve) were selected by flow cytometry.

Through additional experiments we show that rescuing the knockdown via exogenous CDH2 cDNA transfection reverse the effects of the knockdown and restores proliferation. For these experiments we rescue the shRNA effect by expression of an shRNA-resistant form of CDH2. This exogene was optimised using GeneArt gene synthesis online portal. GeneArt gene synthesis portal is a multiparameter RNA and codon optimisation tool that generates optimised synthetic genes that have been reported to be sufficiently different from the wild type sequence without any alterations on gene functionality.[54]

## Functional *in vitro* knockdown, iMSC

iMSC were transduced with sc-29403-V N-cadherin shRNA lentiviral particles (Santacruz Biotechnology) as per manufacturer's protocols and stable cell lines expressing the shRNA were isolated using puromycin selection at 2 μg/mL.

## Mouse transplantation studies

### *Leukemia PDX cell production*

NSG mice were injected with 1x10E$^4$ -1x10$^6$ cells in 20–30μL/mouse in RPMI1640 (SIGMA), 10% FBS (Sigma) intrafemorally (i.f.) directly into the femur bone marrow. During the procedure mice were anesthetised by isoflurane inhalation and provided with analgesia (Carprofen, 5 mg/kg subcutaneously with 29G needle). Mice were humanely killed at a time point prior to adverse health effects as determined by previous studies and the presence of an enlarged spleen visible through the skin of the abdomen. Any mice

that displayed symptoms of leukaemia such as weight loss, anaemia, and hypotonia were immediately humanely killed. PDX cells were harvested from the spleen via cell disruption though a cell strainer (40μm, SLS Ltd.), washed twice in sterile PBS and stored frozen in 10%DMSO;90%FBS (Sigma).

### *Teratoma studies*

5 NSG mice (group size determined from previous studies) per i-niche sample were injected with 5x10$^5$ cells 1:1 in Matrigel (Standard formulation, Corning Inc.) subcutaneously in a volume of 100μL per mouse on the flank with a 29G needle. Tetratoma formation was assessed and measured using calipers at least once weekly. Mice were humanely killed when tumours reached 1.5cm diameter in any direction. Masses were dissected and fixed in formalin for H&E staining by standard methods.

### *Engraftment of i-niche cultured PDX cells*

3x10$^5$ L707D PDX Luc$^+$ GFP$^+$ cells following culture with either iMSC or iANG were injected i.f. into 3 mice/niche. Engraftment was assessed via BLI (see below) and spleen size.

### *ADH-1/Dex* in vivo *efficacy study*

A dose escalation toxicity test was performed in 2 female and 2 male NSG mice to determine a tolerated dose and schedule. 20 NSG mice were injected i.f with 1x10$^4$ L707D PDX Luc$^+$ GFP$^+$ 1x10$^5$ Luc$^+$ GFP$^+$ cells in 20μL media/mouse as described above. Mice injected with this PDX have an event free survival of 4–5 weeks. The study was designed to end 28 days after transplant to maximise the number of PDX cells harvested, to minimise any mouse ill health and to compare treatment effect by comparison of tissue engraftment. Six days after injection, mice were randomised into 4 treatment groups. Mice were housed in a least two cages per treatment group to minimise cage effects. Five mice per group was calculated to be the minimum number to identify a significant difference in BLI total flux between the groups after 3 rounds of dosing. Treatments were administered via intraperitoneal injection using a 29G needle and saline (0.9% w/v) vehicle in a volume of 5 μL/g mouse weight. ADH-1 (Adooq Bioscience) was dissolved in saline just before injection. Dexamethasone sulphate solution was diluted in saline and combined with ADH-1 for a single injection. Groups were given either saline (CV), 3 mg/kg dexamethasone (Dex), 200 mg/kg ADH-1 or Dex/ADH-1 combined (3mg and 200 mg/kg respectively), 1× daily, 5× weekly for 3 weeks. Engraftment was assessed via bioluminescent imaging (IVIS Spectrum, Caliper with Living Image Software). For imaging, mice were injected with 150 mg/kg d-luciferin interperitoneally (*In vivo* Glo, Promega) and anaesthetised with isoflurane. Mice were humanely killed, and spleen cells harvested as described above. A portion of spleen was fixed in formalin for immunohistochemistry. Muscle was removed from all leg and hip bones and bone marrow (BM) cells were isolated by crushing the bones in PBS in a pestle and mortar and washing the bone fragments with PBS.

### *Engraftment assessment of mouse spleen and BM*

Isolated cells were counted and suspended in 0.05%BSA (Roche) in PSB. Cells were stained with mouse CD45 PeCy7 and human CD45 FITC (BD Biosciences) following suppliers' instructions and analysed by flow cytomentry (Attune, Thermo)

Fixed tissues from the efficacy study mice were processed for immunochistochemistry by Cellullar Pathology, RVI Newcastle Hospitals NHS trust using standard methods as follows. Briefly, bones were decalcified using EDTA. Tissues were infiltrated with and embedded in parrafin wax. Sections on slides were staining with either, hematoxylin and eosin (H&E) or, human CD19 antibody using a Ventana BenchMark Ultra (Ventana, Roche), and Universal DAB Detection kit (Ultraview) to produce a brown chromogen at the site of human CD19. Slides were scanned using an Aperio ScanScope (Leica) and images analysised using Leica eSlide manager software.

## QUANTIFICATION AND STATISTICAL ANALYSIS

### Statistics

A two-way analysis of variance, multi comparison with Tukey test was used to compare *in vivo* efficacy group total flux measurements from bioluminescent imaging. One way analysis of variance was used to compare engraftment of BM and spleen and, spleen weight for *in vivo* efficacy treatments. All statistical tests were performed using GraphPad Prism 6.

Cell Reports Medicine, Volume *3*

# Supplemental information

## hiPSC-derived bone marrow milieu identifies a clinically actionable driver of niche-mediated treatment resistance in leukemia

**Deepali Pal, Helen Blair, Jessica Parker, Sean Hockney, Melanie Beckett, Mankaran Singh, Ricky Tirtakusuma, Ryan Nelson, Hesta McNeill, Sharon H. Angel, Aaron Wilson, Salem Nizami, Sirintra Nakjang, Peixun Zhou, Claire Schwab, Paul Sinclair, Lisa J. Russell, Jonathan Coxhead, Christina Halsey, James M. Allan, Christine J. Harrison, Anthony V. Moorman, Olaf Heidenreich, and Josef Vormoor**

## Supplemental information:

**hiPSC-derived bone marrow milieu identifies a clinically actionable driver of niche-mediated treatment resistance in leukemia**

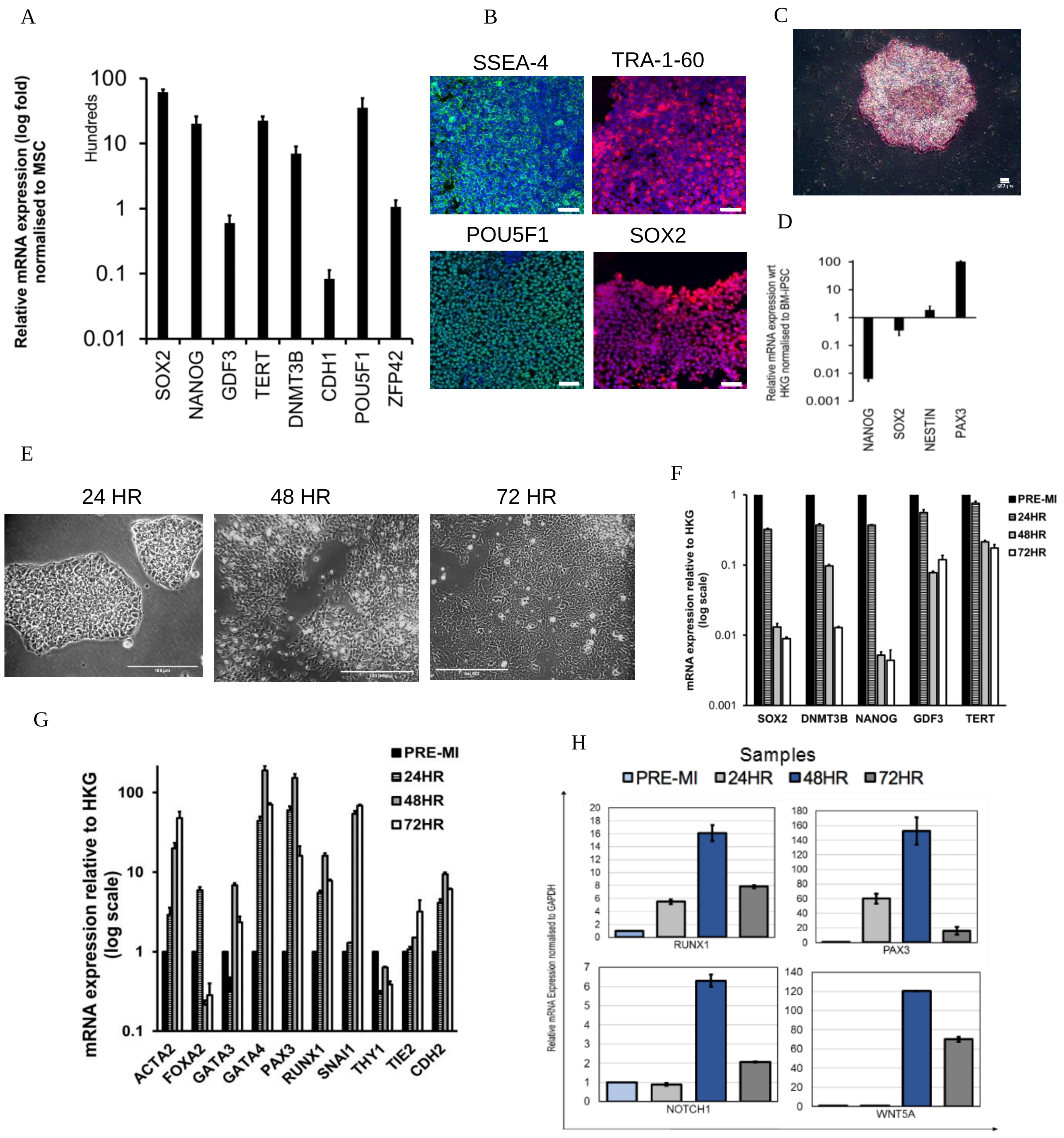


S1. Related to Fig.1. Development and characterization of MYC and virus free BM-iPSC. A. Relative mRNA expression of pluripotent transcripts in BM-iPSC wrt HKG (GAPDH) B. Cell surface marker SSEA4, TRA-1-60 staining and nuclear POU5F1 and SOX2, in feeder-free and xeno-free cultures of BM-iPSC. Scale bar = 100µM C. Image showing BM-iPSC stained for Alkaline phosphatase. Scale bar = 100µM D. mRNA expression wrt HKG (GAPDH) of in vitro differentiation of BM-iPSC derived embryoid bodies E. Photomicrograph of BM-iPSC differentiation into early mesoderm cells over 72 hours. Scale bar = 100µM F. mRNA expression of pluripotent transcripts (HKG = GAPDH) during mesodermal differentiation of BM-iPSC: pre mesoderm induction and at 24, 48 and 72 hours after mesoderm induction. G. mRNA expression (HKG = GAPDH) of mesodermal genes during mesodermal differentiation H. Relative mRNA expression of mesoderm genes RUNX1, PAX3 and NOTCH1 and WNT5A during mesodermal differentiation of BM-iPSC

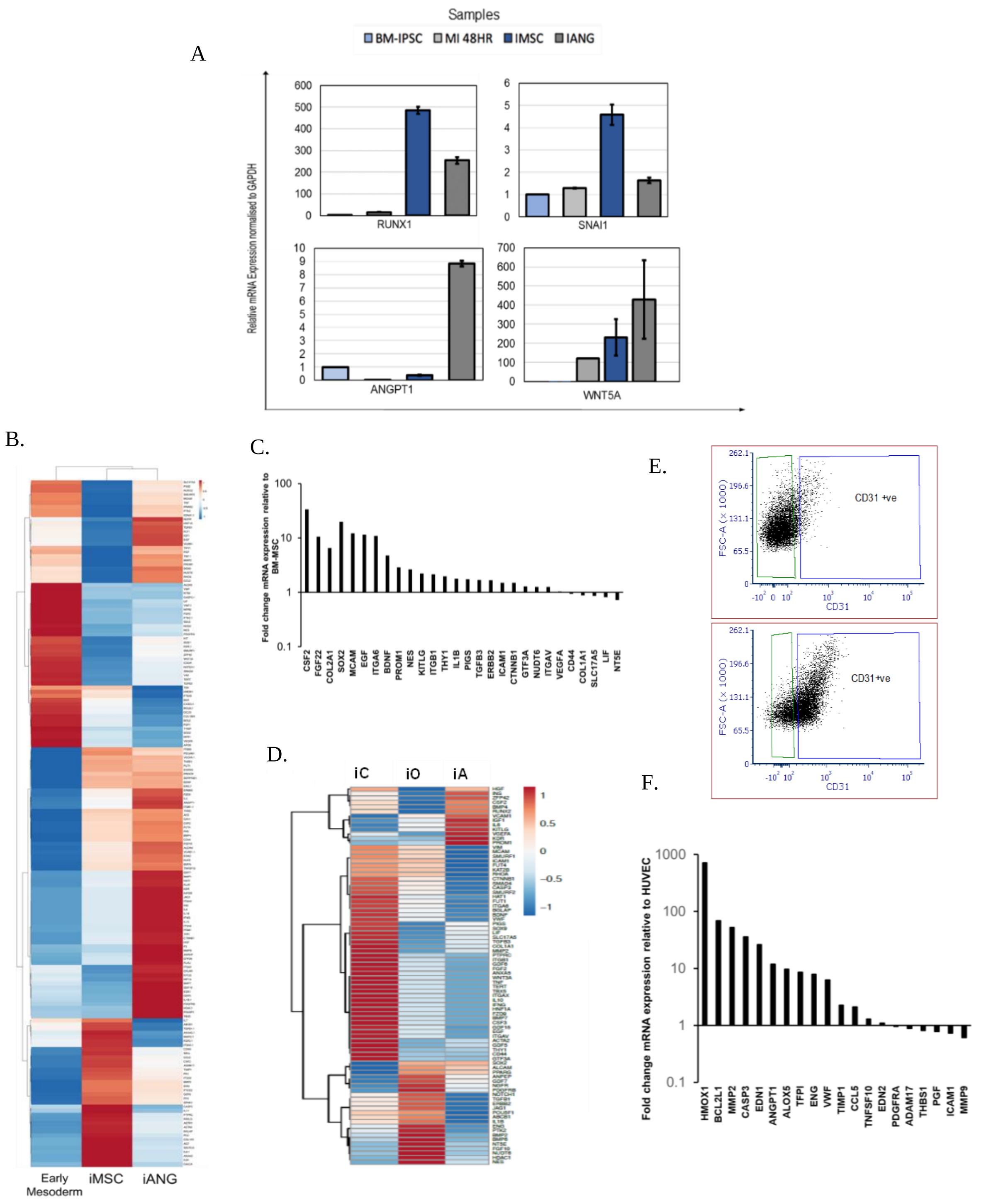


S2. Related to Fig.1. BM-iPSC generate mesenchymal (iMSC) and angiogenic (iANG) niche cells. A. Relative mRNA expression of messenchymal genes RUNX1, SNAI1 and ANGPT1 and WNT5A during in early mesoderm (mesoderm induction, MI at 48 hrs), iMSC and iANG B. BM-iPSC derived early mesoderm, mesenchymal [iMSC] and vascular [iANG] cells demonstrate distinct transcriptomic profiles as evaluated by high throughput qPCR gene expression arrays. C. mRNA expression in iMSC normalised to BM-MSC. D. iMSC differentiate into chondrocytes [iC], osteocytes [iO] and adipocytes [iA] with distinct gene expression profiles E. CD31 expression in iANG cells stained with CD31 antibody (bottom dot plot) versus when stained with isotype control (top dot plot). 54% cells are CD31+ve and 46% are CD31-ve in the stained sample versus 1% within the CD31+ve gate for the relevant isotype control. F. mRNA expression in iPSC-derived endothelia normalised to HUVEC cells.

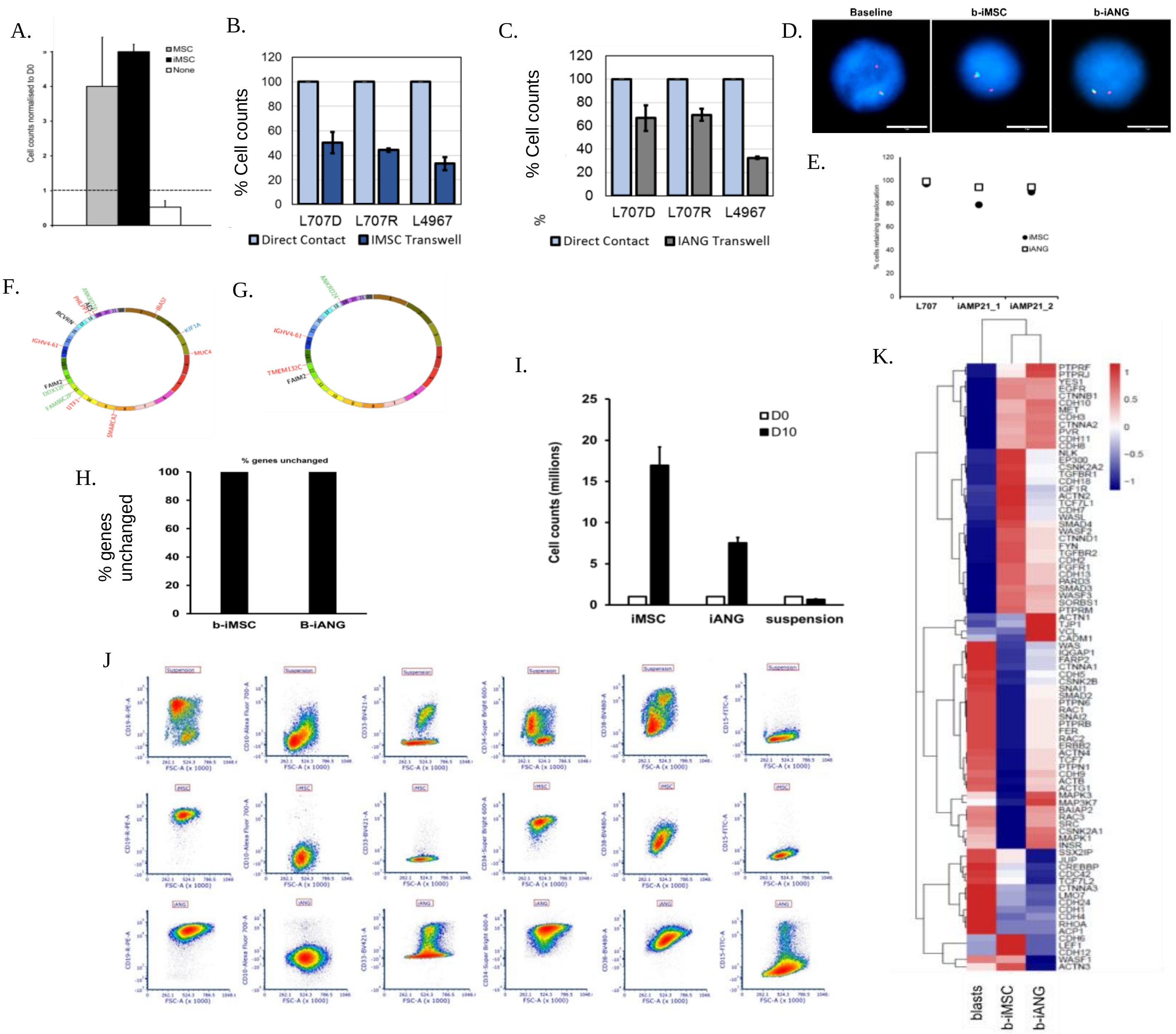

S3. Related to Fig.2. Characterisation of niche primed leukemia cells. A. Counts of patient ALL cells on primary MSC, iMSC and in feeder-free suspension(None) over 7 days. Dotted line = starting cell count. B-C. Cell counts of ALL cells on direct contact and transwell co-cultures with B. iMSC and C. iANG over 7 days with cell counts in direct contact cultures standardized to 100%. D. FISH images of E2A-HLF break-apart probe showing 1 breakpoint and 1 intact loci in baseline and retention of this staining in b-iMSC, b-iANG scale bar = 10 µm. E. Scoring data confirms retention of initial cytogenetic translocation in niche primed ALL. L707 = E2A breakpoint; iAMP21 = additional copies of RUNX1 which is a feature of iAMP21 (abnormal amplification of chromosome 21) samples. F-G. Circos plots showing whole exome changes in patient blasts following co-culture on F. iMSC and G. iANG over 4 weeks. Green=silent:synonymous_variant/non_coding_transcript_exon_variant, black= UTR_variant, red = missense variant, blue = inframe deletion H. % exomes that are unchanged in patient blasts following i-niche co-culture. I. Cell counts from a patient with biphenotypic MLLre leukaemia [MS40] on iMSC, iANG and in niche-free suspension cultures over 7 days. J. Immunophenotyping of MS40 blasts in niche-free suspension culture [top panel] and MS40 blasts primed by iMSC and iANG [middle, bottom panel] after 7 days.K. RNA Sequencing data showing adhesion molecules expression in patient ALL sample L707D blasts before co-culture are compared to blasts following a 7-day co-culture on iMSC (b-iMSC) and iANG (b-iANG).

A.

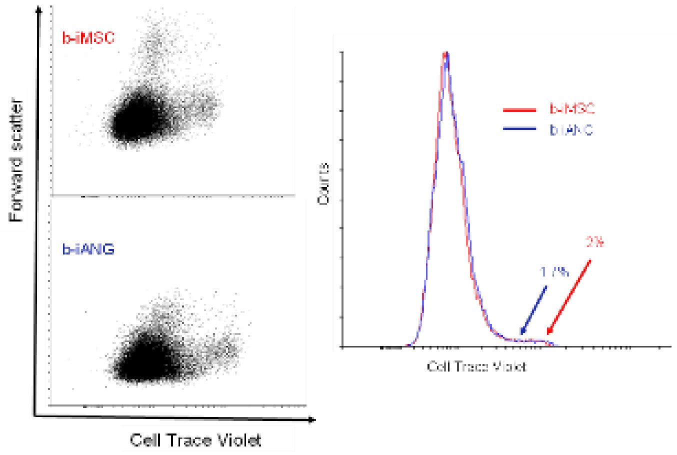


B.

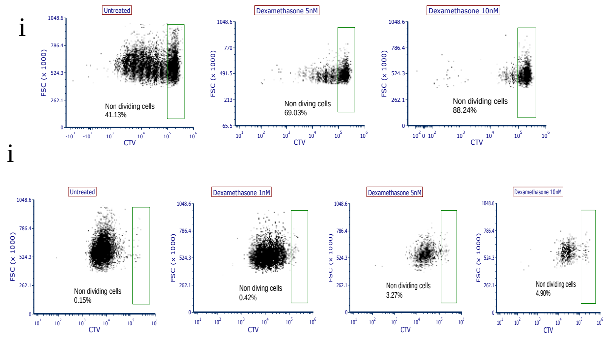


C.

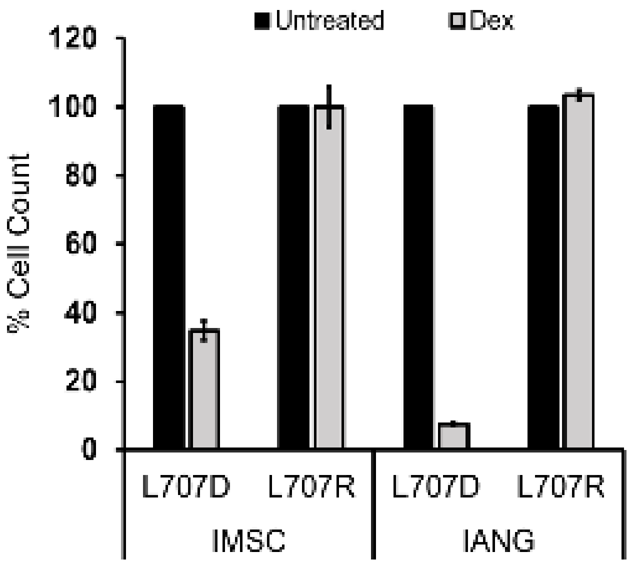


D.

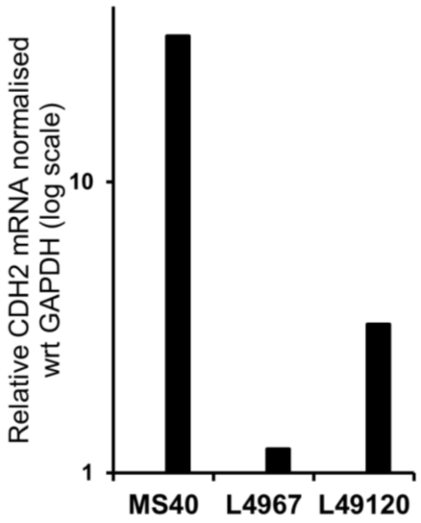


S4. Related to Fig.3. Under Dexamethasone treatment pressure CDH2 is upregulated by iMSC-primed cycling cells. A. Fast and slow dividing niche primed blasts from relapse sample (L707R) on iMSC [red] and iANG [blue] at Day 7. CTV = Cell trace violet dye B. Cell generational tracing dot plots showing slow cycling leukaemia cells when co-cultured on i. iANG and ii. iMSC without and with dexamethasone treatment over a seven day period. C. Cell counts following 10nM dexamethasone treatment on patient leukaemia cells at diagnosis [L707D] and relapse[L707R]. Counts taken at day 7. D. CDH2 mRNA levels in fast cycling iMSC-primed patient leukaemia cells (samples MS40, L4967, L49120) standardised against slow-cycling cells under dexamethasone pressure.

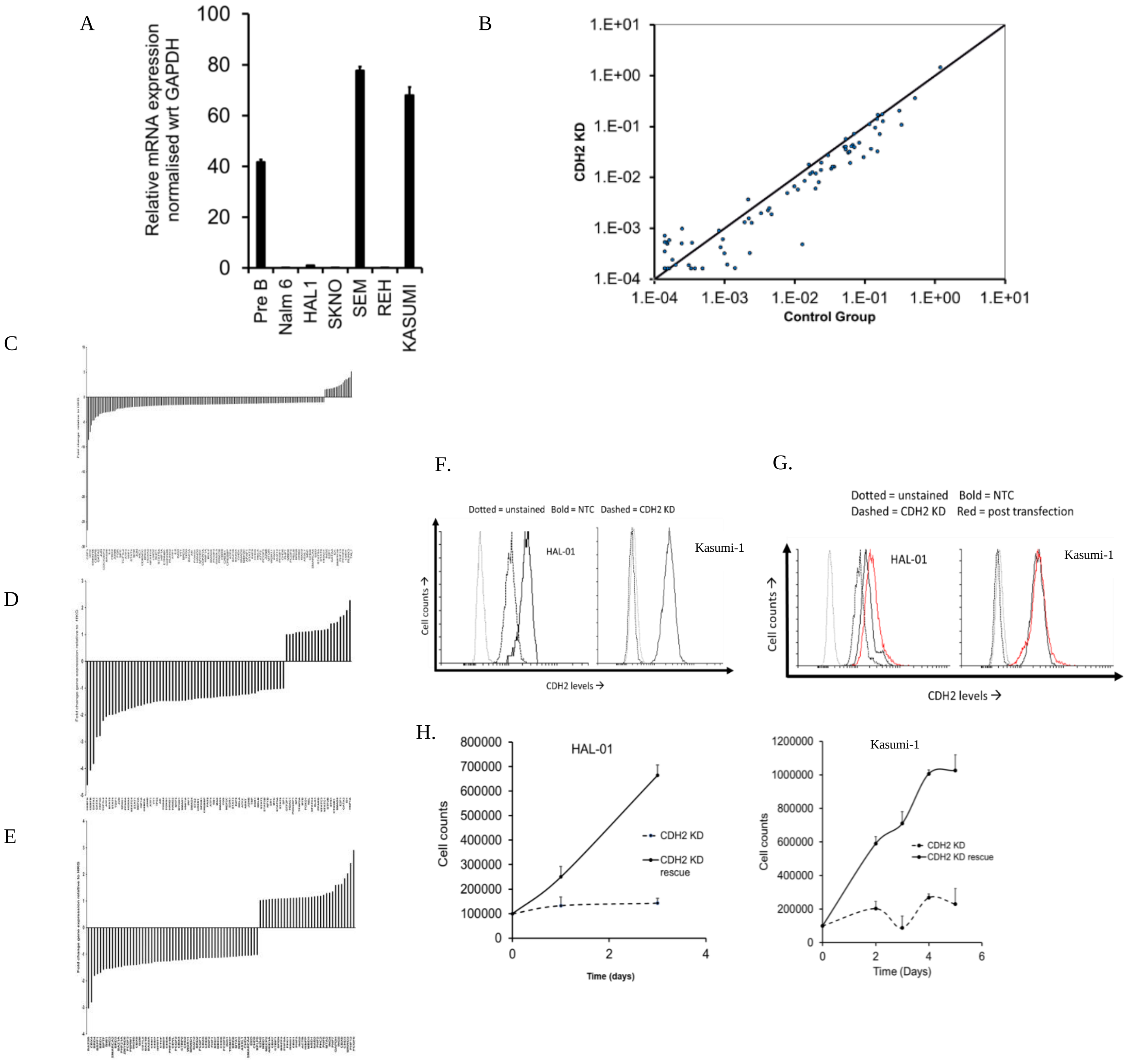


S5. Related to Fig.4. CDH2 drives leukemia proliferation and reduces sensitivity against Dexamethasone. A. CDH2 mRNA levels in cell lines B. Scatter plot showing gene expression in control and CDH2 knockdown in SEM leukaemia cells. C-E. Fold change in qPCR gene expression following CDH2 shRNA knockdown in leukaemic cell line SEM. Genes profiled include C. those that play a role in human leukaemogenesis. D. Transcription factor and E. Chromatin remodelling factors F. Flow cytometry data showing CDH2 expression in ALL (HAL-01) and AML (Kasumi-1) cells following CDH2 knockdown experiments. Bold line represents non targeting control, NTC and dashed line represents CDH2 knockdown cells. G. Rescue of CDH2 protein expression in knockdown cells following transfection with an exogenous optimised CDH2 sequence H. Cell counts in CDH2 shRNA knockdown cells versus CDH2 knockdown cells that have been transfected with the exogenous optimised CDH2 sequence. All experiments performed 48 hours following transfection

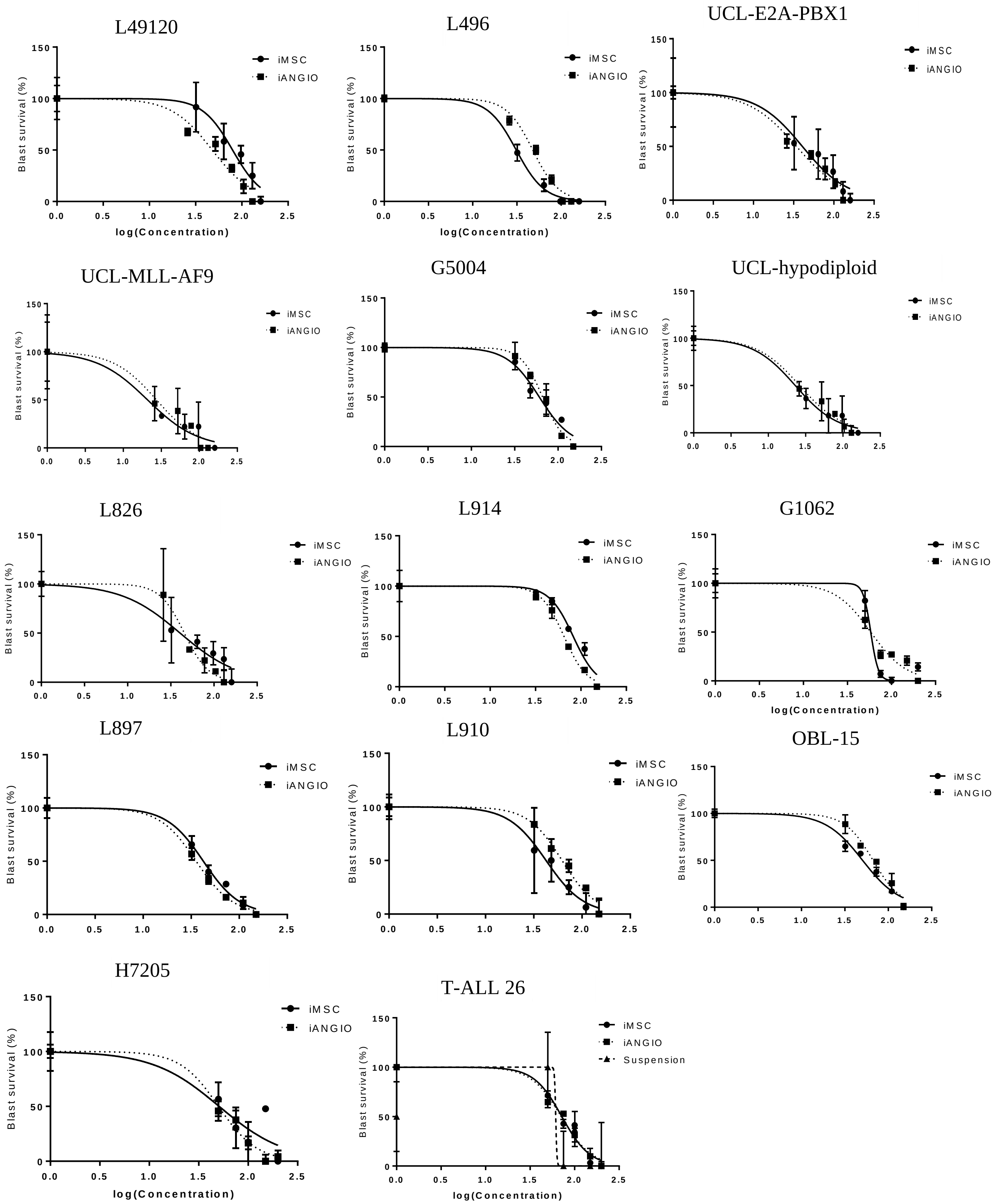


S6. Related to Fig.5. CDH2 antagonist ADH-1 a repurposed compound is identified to show high efficacy on a wide range of patient derived leukemia cells Drug dose response curves with ADH-1 on patient leukaemia samples. Data is from 2 technical repeats

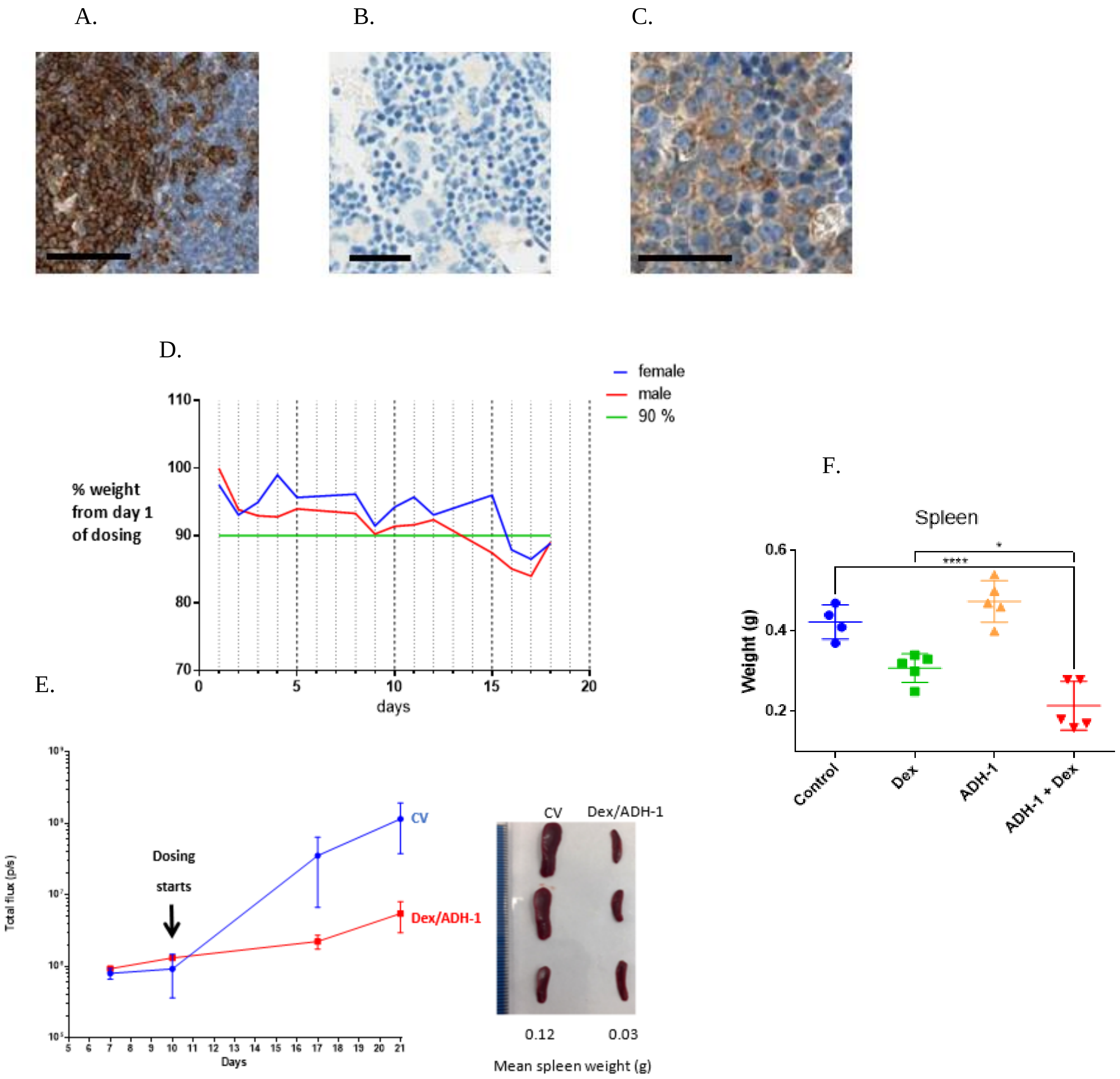


S7. Related to Fig.7. ADH-1 potentiates dexamethasone sensitivity in vivo. CD19 immunohistochemistry staining of sections from A. Human tonsil, positive control; B. Naïve, non-leukaemic mouse bone marrow, negative control, and C. Mouse bone marrow from a L707 PDX transplanted mouse at pre-treatment stage, with BLI total flux equivalent to efficacy study day6 pre-transplant. Scale bar = 50µm. D. Weights of mice (2mice/sex) administered with 3mg/kg Dexamethasone, 200mg/kg ADH-1 (Dex/ADH-1) via intraperitoneal injection, 1x daily, 5x weekly for 3 weeks. Mice appeared healthy with no clinical signs of ill health. Slight weight loss was observed consistent with single drug dexamethasone dosing. E. Dex/ADH-1 feasibility efficacy study. Mean bioluminescent imaging total flux (left) and spleens at 21 days (right) from L707D Luc+ ALL PDX mice, 3 mice/group treated with control vehicle (CV) or Dex/ADH-1 for 9 doses as for D. At 21 days the mean total flux is significantly different between CV and Dex/ADH-1 groups t-test, p=0.002.and spleen at 21 days are smaller. F. Spleen weights of mice treated as indicated. Lines indicate mean and SE, symbols are individual mice. 1 way ANOVA, * p<0.05, ****p<0.00005

## Supplementary tables

| DNA Marker | 6255, BM-MSC | 6255, BM-iPSC | 6257, BM-MSC | 6257, BM-iPSC |
|---|---|---|---|---|
| Amelogenin | X-X | X-X | X-X | X-X |
| D3S1358 | 16-16 | 16-16 | 14-15 | 14-15 |
| THO1 | 9.3-9.3 | 9.3-9.3 | 07-08 | 07-08 |
| D21S11 | 30-30 | 30-30 | 28-29 | 28-29 |
| D18S51 | 16-16 | 16-16 | 16-18 | 16-18 |
| PentaE | 10-15 | 10-15 | 08-13 | 08-13 |
| D5S818 | 12-12 | 12-12 | 11-13 | 11-13 |
| D13S317 | 11-13 | 11-13 | 12-12 | 12-12 |
| D7S820 | 09-11 | 09-11 | 10-10 | 10-10 |
| D16S539 | 11-11 | 11-11 | 10-11 | 10-11 |
| CSF1PO | 10-10 | 10-10 | 10-12 | 10-12 |
| PentaD | 09-13 | 09-13 | 10-12 | 10-12 |
| vWA | 16-18 | 16-18 | 16-17 | 16-17 |
| D8S1179 | 11-12 | 11-12 | 11-15 | 11-15 |
| TPOX | 08-11 | 08-11 | 08-11 | 08-11 |
| FGA | 19-20 | 19-20 | 19-25 | 19-25 |

Table S1. Related to Fig.1. Microsatellite profiling confirms the BM-iPSC are an identical match to the parental primary bone marrow mesenchymal stroma cells for the 16 microsatellites tested including amelogenin, a sex marker

| Mouse number | from culture with | IVIS total flux (p/s) | | Killed | spleen wt (g) | Liver wt (g) |
|---|---|---|---|---|---|---|
| | | 2 weeks | 3 weeks | | | |
| 1 | iMSC | 1.14E+06 | 2.26E+06 | 4.5 weeks | 0.57 | 1.97 |
| 2 | iMSC | 1.17E+06 | 1.03E+06 | 4.5 weeks | 0.52 | 2.18 |
| 3 | iMSC | 1.24E+06 | 1.82E+06 | 4.5 weeks | 0.51 | 2.12 |
| | | Av | 1.71E+06 | | 0.53 | 2.09 |
| 4 | iANG | 1.33E+06 | 1.71E+06 | 4.5 weeks | 0.35 | 1.81 |
| 5 | iANG | 6.95E+05 | 2.10E+06 | 4.5 weeks | 0.32 | 1.86 |
| 6 | iANG | 1.01E+06 | 1.78E+06 | 4.5 weeks | 0.37 | 2.09 |
| | | Av | 1.86E+06 | | 0.35 | 1.92 |

Table S2. Related to Fig. 3. B. Bioluminescent imaging total flux and organ weights of NSG mice transplanted intrafemorally with 300,000 L707D PDX cells following their culture with i-niche. Total body engraftment (measured by total flux) did not differ significantly between the two niche grown PDX however the spleens of mice transplanted with iANG grown cells were significantly smaller (measured by weight) suggesting a reduction in system engraftment of cells to the BM. The liver in this PDX model contains very few engrafted cells so is a control for mouse size.
